# Dissecting the Genetic Architecture of Shoot Growth in Carrot (*Daucus carota* L.) Using a Diallel Mating Design

**DOI:** 10.1534/g3.117.300235

**Published:** 2017-11-29

**Authors:** Sarah D. Turner, Paul L. Maurizio, William Valdar, Brian S. Yandell, Philipp W. Simon

**Affiliations:** * Department of Horticulture, University of Wisconsin-Madison, Wisconsin 53706; † Curriculum in Bioinformatics and Computational Biology, University of North Carolina, Chapel Hill, North Carolina 27599; ‡ Department of Genetics, University of North Carolina, Chapel Hill, North Carolina 27599; § Department of Statistics, University of Wisconsin-Madison, Wisconsin 53706; ** United States Department of Agriculture–Agricultural Research Service, Vegetable Crops Research Unit, Madison, Wisconsin 53706

**Keywords:** diallel, Bayesian mixed model, genetic architecture, multiple imputation, heterosis, degree of dominance, multiparental populations, MPP

## Abstract

Crop establishment in carrot (*Daucus carota* L.) is limited by slow seedling growth and delayed canopy closure, resulting in high management costs for weed control. Varieties with improved growth habit (*i.e.*, larger canopy and increased shoot biomass) may help mitigate weed control, but the underlying genetics of these traits in carrot is unknown. This project used a diallel mating design coupled with recent Bayesian analytical methods to determine the genetic basis of carrot shoot growth. Six diverse carrot inbred lines with variable shoot size were crossed in WI in 2014. F1 hybrids, reciprocal crosses, and parental selfs were grown in a randomized complete block design with two blocks in WI (2015) and CA (2015, 2016). Measurements included canopy height, canopy width, shoot biomass, and root biomass. General and specific combining abilities were estimated using Griffing’s Model I, which is a common analysis for plant breeding experiments. In parallel, additive, inbred, cross-specific, and maternal effects were estimated from a Bayesian mixed model, which is robust to dealing with data imbalance and outliers. Both additive and nonadditive effects significantly influenced shoot traits, with nonadditive effects playing a larger role early in the growing season, when weed control is most critical. Results suggest the presence of heritable variation and thus potential for improvement of these phenotypes in carrot. In addition, results present evidence of heterosis for root biomass, which is a major component of carrot yield.

Carrots are the seventh most consumed fresh vegetable in the United States, with an annual per capita consumption of 3.9 kg [USDA Economic Research Service (ERS) 2016] and a production value of $762 million USD in 2015 (USDA National Agricultural Statistics Service (NASS) 2016). In the US, the high α- and β-carotene content in carrots is a leading source of dietary provitamin A (Block 1994; Simon *et al.* 2009), which is essential for healthy immune function, vision, reproduction, and cellular communication (Institute of Medicine, Food and Nutrition Board 2001; Johnson and Russel 2004; Solomons 2012). Despite the economic and dietary importance of carrots, crop establishment and productivity remain limited by erratic germination, slow seedling growth, and delayed canopy closure (Rubatzky *et al.* 1999). This growth habit, coupled with thin, highly segmented leaf laminae, competes ineffectively with weeds for nutrients, water, and light, resulting in yield losses caused by reductions in root size and marketability (Bellinder *et al.* 1997; Bell *et al.* 2000). Furthermore, in a survey of weed competitiveness in 25 crops, carrot had the largest reduction in yield under weed pressure (van Heemst 1985).

To limit yield loss, carrots have an extended critical weed-free period ranging from 3 to 6 wk, during which chemical and hand weeding are necessary (Swanton *et al.* 2010). Hand weeding, while effective, is disruptive to plant growth and requires intensive labor, with estimated costs exceeding 4000 USD/ha (Bell *et al.* 2000). For organic systems, which constitute 14.4% of carrot acreage in the US [USDA National Agricultural Statistics Service (NASS) 2016], hand weeding is typically the primary method of weed management. Even in conventional systems, few herbicides are labeled for carrots and can only be applied when plants reach a threshold height (*e.g.*, linuron) or have five to six true leaves (*e.g.*, metribuzin), by which point weeds have exceeded control stages (Bellinder *et al.* 1997).

Cultivars with increased weed competitiveness offer a low cost, nonchemical, and sustainable addition to an integrated weed management program (Pérez de Vida *et al.* 2006; McDonald and Gill 2009). Improved competitive ability has been linked to traits that increase resource allocation, such as height and biomass accumulation, in other densely planted crops such as maize (Mohammadi 2007; Zystro *et al.* 2012), rice (Ni *et al.* 2000; Fischer *et al.* 2001; Pérez de Vida *et al.* 2006), wheat (Lemerle *et al.* 1996; Murphy *et al.* 2008), and soybean (Bennett and Shaw 2000; Jannink *et al.* 2000). While improvement of these traits offers a potential solution for weed management, it is unknown how these traits are inherited in carrot or how they influence marketability (*e.g.*, root biomass accumulation).

Many carrot breeding programs employ the inbred-hybrid method, which is enabled by the widespread availability of cytoplasmic male sterility (CMS) in carrot germplasm (Peterson and Simon 1986; Simon 2000). Accordingly, heterosis is considered a promising mechanism to increase yield in carrot (Rubatzky *et al.* 1999), and hybrid vigor has been documented for important traits such as carrot root yield (Duan *et al.* 1996; Suh *et al.* 1999; Simon and Strandberg 1998; Jagosz 2011, 2012; Simon *et al.* 1982), carotenoid content (Santos and Simon 2006), and resistance to Alternaria leaf blight (Simon and Strandberg 1998). However, the development and adoption of heterotic groups in carrot remains limited, and has not been described in detail. Thus, carrot breeding efforts for unimproved traits with economic importance, such as weed competitive ability, will benefit from an understanding of combining ability and reciprocal cross effects in diverse genetic backgrounds.

The diallel mating design, which consists of pairwise combinations among a group of inbred parents, is a natural first step to identify informative testers, to develop heterotic groups, and to determine the primary genetic control for complex traits (Hayman 1954a,b; Gardner and Eberhart 1966). The diallel was first introduced to plant breeding in 1942 by Sprague and Tatum, who defined general combining ability (GCA) and specific combining ability (SCA) as the relative proportions of additive and nonadditive (*i.e.*, dominance and epistatic) genetic variation for a trait, respectively (Sprague and Tatum 1942; Hayman 1954a,b; Griffing 1956a,b). Although it is a valuable tool in plant breeding, application of the diallel mating design in practice remains challenging due to resource constraints and the difficulty of traditional, usually frequentist, analyses. Choice among these methods depends on many factors, such as the goals of the researcher, the type of diallel mating design (*e.g.*, full, half, or sparse), and the selection of parental lines from either fixed or random mating populations. The challenges of applying these traditional approaches can be attributed to the complexity of the models, controversy over the proper interpretation of results, and to the task of choosing among the numerous methods described in the literature, which include ANOVA-based approaches (Hayman 1954a,b; Griffing 1956a; Gardner and Eberhart 1966; Wu and Matheson 2000), mixed effects modeling (Xiang and Li 2001), the use of minimum norm quadratic unbiased estimators (Zhu and Weir 1996), and REML (Möhring *et al.* 2011).

Of the available methods for diallel analysis, the general linear model approach proposed by Griffing (1956b) remains among the most prevalent in plant breeding for its relative simplicity and relevance to crop improvement (Hallauer and Miranda Filho 1981; Christie and Shattuck 1992; Zhu and Weir 1996; Viana 2000; Wu and Matheson 2000). When applied appropriately, Griffing’s analysis provides reliable estimates of GCA, SCA, and reciprocal cross effects (Christie and Shattuck 1992). The base model can also be modified to include interactions of these main effects across environments, which is important for assessing the stability of hybrid performance (Singh 1973; Lin *et al.* 1977; Zhang and Kang 1997). However, when parental lines are fixed, Griffing’s method is not robust in addressing common issues encountered in field experiments, such as missing data, imbalance, and outliers (Wu and Matheson 2000; Xiang and Li 2001). For fixed effects models, imbalance is typically addressed by either list- or pair-wise deletion, or by implementing a design matrix to specify the missing crosses (*e.g.*, Wu and Matheson 2000), both of which may reduce the number of observations and power of the analysis. Alternatively, mixed effects models, being more robust to imbalance and outliers, can provide reliable estimates for the parameters of interest (*e.g.*, Robinson 1991), but many of these implementations for diallel analysis have been attached to restrictive assumptions, and may require that parents are selected from a random mating population (*e.g.*, Zhu and Weir 1996; Xiang and Li 2001; Möhring *et al.* 2011).

In this context, a number of conceptual and practical limitations are overcome by the use of computationally intensive Bayesian methods (Greenberg *et al.* 2010; Lenarcic *et al.* 2012). Although the modeling is more complex, these approaches offer numerous advantages. Practically, the use of Markov Chain Monte Carlo (MCMC) sampling can provide great flexibility regarding model complexity (Greenberg *et al.* 2010; Lenarcic *et al.* 2012). Conceptually, the Bayesian approach provides a natural justification for the random effects formulation, as a hierarchical prior on the effects distribution, that may be independent of how units were actually sampled (Lenarcic *et al.* 2012; and, *e.g.*, Gelman and Hill 2007). The application of a Bayesian approach for diallel analysis also improves the biological interpretability of results, and expands the types of questions that can be addressed by researchers.

In this study, we used two complementary approaches to elucidate the relative importance of genetic parameters for shoot growth in carrot: the frequentist, fixed effects methodology developed by Griffing (1956b), which is a standard method of analysis for plant breeders, and, as such, serves as a familiar reference point; and the more recent Bayesian mixed model (BayesDiallel) developed by Lenarcic *et al.* (2012), which is not currently used in plant breeding. As part of Griffing’s analysis, we also describe the use of multiple imputation to fill in missing values. Multiple imputation allows the use of a fixed model by providing plausible, unbiased estimates that are informed by all available data, avoiding unnecessary reductions in sample size and statistical power. Imputation and interpretation of imputed data are straightforward, and, thus, provide an updated means for breeders to use Griffing’s or other models sensitive to imbalance. Alternatively, the BayesDiallel model provides a more robust and detailed analysis of diallel data, with a more direct interpretation of genetic effects, but the complexity of the model may inhibit its adoption by plant breeders. By presenting the results from BayesDiallel alongside the results from the more traditional Griffing’s analysis, we aim to facilitate the adoption of the BayesDiallel approach by the plant breeding community. Thus, the primary goals of this work were (1) to estimate the inheritance of shoot growth in carrots as a resource to inform selection strategies, identify useful testers, and assess hybrid performance; and (2) to present an applied framework for diallel analysis of multiple environment data using Bayesian modeling.

## Materials and Methods

### Plant material and measurements

**Figure 1:**
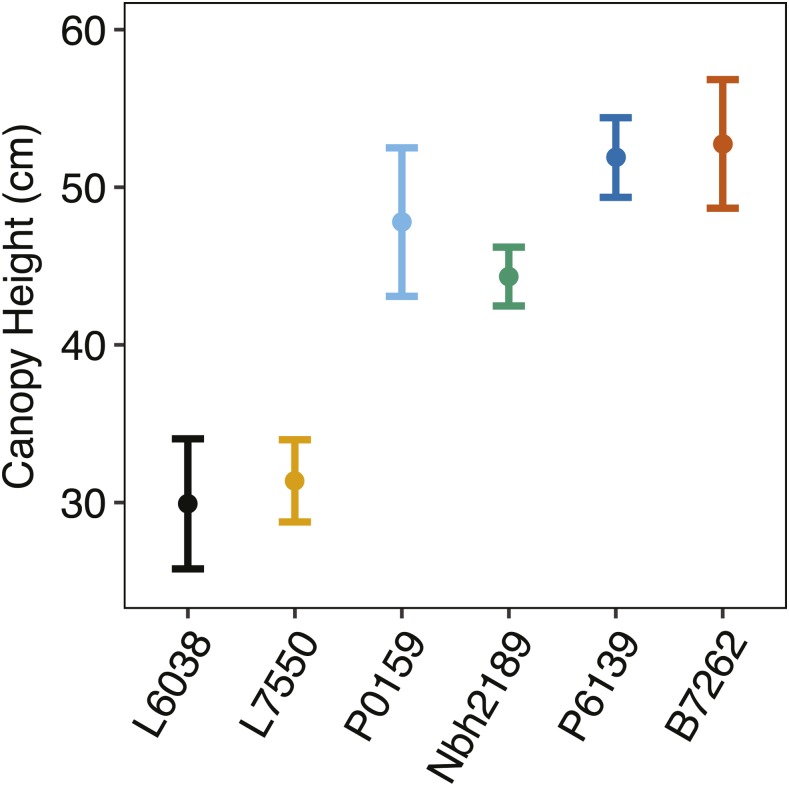
Variation in means and 95% confidence intervals for canopy height (130DAP) among carrot inbred lines used in this study.

Six inbred lines, with canopy heights ranging from short (29.9 cm) to tall (52.8 cm), were selected from the USDA-VCRU carrot breeding program and included both male sterile (A-line) and male fertile (B-line) breeding stocks for inbred lines L6038, L7550, P0159, Nbh2189, P6139, and B7262 (Figure 1, Supplemental Material, Figures S1 and S2 in File S1, and Table 1). Male sterile A-lines expressed the petaloid CMS system, which is widely used in North American breeding programs due to its environmental stability (Simon *et al.* 2008). Inbred parents were combined in all pairwise combinations for a total of 36 combinations at the West Madison Agricultural Research Station (Madison, WI) in 2014. The resulting F1 progenies, reciprocals, and parental selfs were grown in a randomized complete block design (RCBD) with two blocks. Field sites included the Hancock Agricultural Research Station (Hancock, WI; 2015) and the University of California Desert Research and Extension Center (Holtville, CA; 2015 and 2016). Carrots were grown on 1.5-m plots with 1 m between-row spacing.

**Table 1:** Carrot traits evaluated, their range among parents, and number of complete observations for each environment in this study

Phenotype	Measurement	Unit	Parental Range		Number of Complete Observations*^a^*		
				Data Transformation	WI2015	CA2015	CA2016
Canopy height	Three points within the plot	Centimeters (cm)	29.9–52.8	None	50	72	69
Canopy width	Three points within the plot	Centimeters (cm)	41.5–61.3	None	50	72	69
Shoot biomass	Fresh and dry	Grams (g)	6.43–21.3 (fresh)	ln(x)	49	72	68
			1.02–3.39 (dry)				
Root biomass	Fresh and dry	Grams (g)	29.0–64.9 (fresh)	ln(x)	49	72	68
			4.22–8.64 (dry)				
Shoot to root ratio	Shoot biomass/root biomass (dry)	Grams (g)	0.23–0.64	ln(x)	49	72	68

a 72 observations possible per environment (36 entries × 2 replications).

Measurements of each trait were recorded for three subsamples per block, which were averaged prior to analysis, and are summarized in Table 1. Canopy height and width were measured at midseason, 80 d after planting (DAP), and at harvest, 130 DAP. At harvest, fresh and dry biomass were recorded separately for both shoot and root tissue. For dry biomass, samples were dried at 60° in a forced-draft oven until reaching a constant weight. A natural log transformation, ln(x), was applied to biomass measurements to make the data distribution symmetric and stabilize the variance. Planting density was recorded on a discrete 0–3 scale, with 0 indicating no plants, 1 = <25 plants m^−1^, 2 = between 25 and 50 plants m^−1^, and 3 = >50 plants m^−1^.

### Statistical analyses

Diallel data for each phenotype was analyzed using two complementary approaches: a traditional fixed effects frequentist analysis after Griffing (1956b), which, owing to its requirement that data are complete and balanced, was combined here with a multiple imputation procedure; and the recent Bayesian mixed model decomposition of Lenarcic *et al.* (2012), performed on the raw (unimputed) data. These are described in detail below. All analyses were performed in R. 3.3.2 (R Core Team 2016).

#### Multiple imputation of missing data for Griffing’s analysis:

To compensate for imbalance, missing data (Figure S3 in File S1 and Table 1) was imputed using the Multivariate Imputation by Chained Equations package (R package mice; R/mice) (van Buuren and Groothuis-Oudshoorn 2011), and specifically using that package’s predictive mean matching method (PMM), which is a general purpose, stochastic regression technique that is suitable for numeric data (Little 1988). The predictors used for PMM were chosen based on recommendations in the R/mice documentation, and included female parent, male parent, cross, location, replication, planting density, and numeric measurements with complete data. The values imputed by the PMM were generated by running its associated MCMC sampler until it reached a stationary distribution (usually at ∼40 iterations; Figure S4 in File S1), and then recording sampled values from a later iteration (*e.g.*, iteration 70). This was repeated *m* = 50 times to generate *m* imputed data sets.

#### Griffing’s analysis:

Each of the *m* imputed data sets was analyzed using Griffing’s Method I, Model I (Griffing 1956b), which treats genotypes and blocks as fixed effects and has the base model:

$$y_{ijk}=\mu +g_{j}+g_{k}+s_{jk}+r_{jk}+\varepsilon_{ijk},$$

where μ is the population mean, $g_{j}$ and $g_{k}$ are the GCA effects for the *j*th and *k*th parents, respectively, $s_{jk}$ is the SCA effect for the cross of the *j*th and *k*th parents $\left(s_{jk}=\;s_{kj}\right),$ $r_{jk}$ is the reciprocal effect for the cross of the *j*th and *k*th parents ($r_{jk}=-r_{kj}$), and $\varepsilon_{ijk}\;$ is the environmental effect for the *ijk*th observation. Griffing’s analysis was run using the diallel1 function in the R package plantbreeding (Rosyara 2014), which we modified to include environmental effects and genotype × environment interactions (G × E) as previously described by Singh (1973), Lin *et al.* (1977), and Zhang and Kang (1997). Mean squares and approximate *F*-tests were pooled following the method proposed by Raghunathan and Dong (2011, unpublished data) (Table S1 in File S1). Estimates for GCA, SCA, and reciprocal effects were combined according to Rubin’s rules (Rubin 1987) and as implemented in R/mice (van Buuren and Groothuis-Oudshoorn 2011).

The proportion of additive to nonadditive genetic variation was estimated from the fixed model using Baker’s ratio of $2MS_{GCA}:2MS_{GCA}+MS_{SCA},$ with values close to unity suggesting higher predictability based solely on GCA (Baker 1978). Because the inclusion of parental lines in Method I can cause an upward bias in estimates of combining ability variances, we also report Baker’s ratio using Method III, which includes F1 hybrids and reciprocal F1s, but excludes parental lines (Hayes and Paroda 1974).

#### Identification of tester lines:

Informative tester lines were identified for each phenotype using the GGE biplot method as specified by Yan and Hunt (2002). Biplots were constructed from a two-way data matrix of means for each phenotype, with parental lines treated as both entries and testers. The R package GGEBiplotGUI (Frutos *et al.* 2014) was used to generate biplots with symmetrical scaling, tester centering, and the “discriminativeness against representativeness” view, which establishes an axis representative of the average tester. Useful testers were identified as parental lines which were both discriminating (*i.e.*, able to rank the combining abilities of other parental lines; represented on the biplot by the longest vector), and the most representative, reflecting the average of all parental lines (*i.e.*, zero or minimal SCA effects; represented by the vector with the least projection onto the average tester axis) (Yan and Hunt 2002). Additional details and biplots for each trait are provided in File S1.

#### Bayesian mixed model for diallel analysis:

A brief overview of the BayesDiallel model is provided below. Additional details are available in the original BayesDiallel manuscript by Lenarcic *et al.* (2012), which provides comprehensive explanations of the BayesDiallel model, including the theoretical and practical justifications, and a thorough comparison with Griffing’s method. Conceptually, the BayesDiallel model can be broken down into three components: (1) a mixed model with >5 variance components, (2) a set of priors on the variance components which make the model Bayesian, and (3) a MCMC algorithm that fits the model.

For this study, raw data for each phenotype ($y_{i}$), measured for individuals i∈{1,…,n} with female parent j and male parent k, were decomposed into additive effects (a), effects of being inbred both as an overall effect ($\beta_{\text{inbred}}$) and a parent-specific deviation (b), maternal effects (m), cross-specific symmetric effects (v; *i.e.*, common across reciprocals), and cross-specific asymmetric effects (w; *i.e.*, reciprocal-specific), as described by Lenarcic *et al.* (2012) and Crowley *et al.* (2014), and implemented as a Gibbs sampler in the R package BayesDiallel. Specifically, we used BayesDiallel’s full unsexed model (“fullu”):

$$\begin{array}{c} y_{i}=\mu +\underset{\text{user}\;\text{fixed}}{\underset{︸}{x_{i}^{T}\beta }}+\underset{\text{user}\;\text{random}}{\underset{︸}{\sum_{r=1}^{R}u_{i}^{\left(r\right)}}}\;+\underset{\text{additive}}{\underset{︸}{a_{j\left[i\right]}+a_{k\left[i\right]}}}+\underset{\text{inbred}}{\underset{︸}{I_{\left\{j\left[i\right]=k\left[i\right]\right\}}\left(\beta_{\text{inbred}}+b_{j\left[i\right]}\right)}}+\underset{\text{maternal}}{\underset{︸}{m_{j\left[i\right]}-m_{k\left[i\right]}}}\\ +\underset{\text{symmetric}}{\underset{︸}{I_{\left\{j\left[i\right]\neq k\left[i\right]\right\}}v_{\left(jk\right)\left[i\right]}}}+\underset{\text{asymmetric}}{\underset{︸}{I_{\left\{j\left[i\right]<k\left[i\right]\right\}}w_{\left(jk\right)\left[i\right]}}}+\varepsilon_{i}, \end{array}$$

where j[i], k[i] and (jk)[i], respectively, denote the female, male, and female-male combination relevant to individual i, and each group of effects parameters is modeled from its own random effects distribution, *e.g.*, $a_{j}∼N\left(0,\tau_{a}^{2}\right)$ with $\tau_{a}^{2}∼Inverse\;\chi^{2}$ (d.f. = 0.5, mean = 1). Fixed effects for covariates $x_{i}$ and fixed effects μ and $\beta_{\text{inbred}}$ are modeled as having vague priors of $N\left(0,10^{3}\right),$ while R additional random-effect components are included as $u_{i}^{\left(r\right)}∼N\left(0,\;\tau_{r}^{2}\right)$ for each r∈{1,…,R}.

The above model was fitted in BayesDiallel using a MCMC Gibbs sampler with five chains, 10,000 iterations, and a burn-in of 1000. Planting density (0–3 scale, 0 = no plants, 1 = low, 3 = high) was included as a fixed covariate to capture linear trends and as a random effect to estimate deviations from linearity. Location (WI2015, CA2015, and CA2016) was included as a random effect. To compare rankings for hybrids across growing environments as a measure of genotype by environment (G × E) interaction, the BayesDiallel model was also applied to data stratified by location.

The model parameters in BayesDiallel were previously described by Lenarcic *et al.* (2012) and are summarized as follows. In the “fullu” model described above, additive effects ($a_{j}$) are modeled as random effects (as in, for example, Zhu and Weir 1996), and provide estimates of the dosage effect for a given parent *j* in combination with another parent *k*. If a strictly additive model is run in BayesDiallel (the “a” model), $a_{j}\;$corresponds to GCA as defined by Sprague and Tatum (1942); but this comparison becomes less constructive when other effects are incorporated into the BayesDiallel model. Building upon the “a” model, the “Bab” model incorporates parent-specific inbred deviations, modeled as an inbred penalty random effect, $b_{j},$ with a common distribution centered at a fixed effect ($\beta_{\text{inbred}}$). This differs from conventional models of dominance in that heterosis is modeled as inbred-specific deviations from heterozygote-based predictions; that is, homozygotes (*i.e.*, parental selfs), which are a minority in the diallel, are treated as a special class. Parent-of-origin effects ($m_{j}$) are then modeled as symmetric (random effect) deviations around the “Bab” model to generate the “Babm” model. Finally, statistical interactions between pairs of parents are modeled as two types of random effect departures from the “Babm” model: cross-specific symmetric effects (*v*), which model differences specific to a given cross, regardless of parental inheritance and independent of reciprocal effects (*i.e.*, crosses *jk* and *kj* have the same effect); and cross-specific asymmetric effects (*w*), which model deviations from cross-specific symmetric effects due to differences between reciprocal crosses (*i.e.*, *jk* and *kj* have different effects).

#### Estimating the degree of dominance:

Although BayesDiallel does not model an explicit term for dominance, it is straightforward to define dominance as a function of existing parameters, and, by applying that function to repeated MCMC samples, estimate its full posterior. Following Maurizio *et al.* (2017), we define an aggregate signal of dominance within each pairwise cross using an adaptation of the degree of dominance ($a_{CR}$), as defined by Comstock and Robinson (1948) and applied to maize by Gardner and Lonnquist (1959). In this case, the degree of dominance of parent A when combined with a second parent B is:

$$a_{CR}=1-2\times \left(\frac{y_{BB}-y_{Bb}}{y_{BB}-y_{bb}}\right),$$

where $y_{BB},$ $y_{Bb},$ and $y_{bb}$ are the posterior expectations of predicted phenotype values for the parental contributions of two alleles at a given locus (the dominant *B* allele and the recessive *b* allele), which are considered in this case as contributions from a given parental genome instead of individual loci. Values for $a_{CR}$ can be interpreted as follows: that parent A is pseudo-under-recessive to parent B ($a_{CR}$ << −1); that A is recessive to B ($a_{CR}$ = −1); that A is additive (*i.e.*, codominant) with B ($a_{CR}\;$ = 0); that A is dominant (or completely dominant) to B ($a_{CR}$ = 1); that A is pseudo-overdominant to B ($a_{CR}$ >> 1). This estimate only captures pseudo-overdominance, as we are unable to distinguish between true overdominance and the repulsion-phase linkage of loci with complete or partial dominance (Gardner and Lonnquist 1959). Appendix A describes the calculation of the Comstock-Robinson degree of dominance, and its relation to the dominance index, D , originally presented by Kacser and Burns (1981) and Wright (1934) and as recently applied by Maurizio *et al.* (2017).

#### Diallel variance projection as a repeatability-like measure:

In order to report the relative contribution of each diallel inheritance class to a given phenotype, Crowley *et al.* (2014) proposed the diallel variance projection (VarP). This approach uses the posterior predictive distribution of effects from BayesDiallel to simulate future, complete, perfectly balanced diallels of the same parental lines. In each simulated dataset, the contribution of each inheritance class (additive, inbred, *etc*.) is then calculated as its sum of squares (SS) divided by the total phenotype SS. The resulting proportion, the VarP, is similar to the traditional estimates of repeatability described by Mather and Jinks (1982) and Lynch and Walsh (1998), but with two important differences: (1) it is explicitly prospective, in that it seeks to describe how much additive effects, say, would impact a future experiment; and (2) its estimation is more precise, since it is calculated as a function primarily of the effects parameters (*e.g.*, $a_{1},\;a_{2},\ldots ,a_{6}$), which are well informed by the data, rather than of the variance components ($\;\tau_{a}^{2},\;\;\tau_{m}^{2},$ *etc*.), which are typically not (Sorensen and Gianola 2002; Crowley *et al.* 2014; Furlotte *et al.* 2014). VarPs are calculated from multiple posterior draws leading to a complete posterior distribution of the VarP for each inheritance class, summarized here as highest posterior credibility intervals. Credibility intervals that include zero are interpreted as not contributing positive, nonzero information to the prediction of $y_{i},$ whereas credibility intervals excluding zero provide strong evidence that an effect is important to the model.

### Data availability

All data and code used in this study are available on GitHub at https://github.com/mishaploid/carrot-diallel. Supporting information for each trait [parental phenotypes, biplot analysis, GCA, SCA, highest posterior density (HPD) intervals, degree of dominance, and hybrid ranks] is provided in File S1.

## Results

### Imputation of missing data

There was a high incidence of missing data due to variation in seed production and disease pressure (Figure S3 in File S1 and Table 1). A large proportion of missing data occurred in the WI2015 environment, which was subject to severe infestation by Alternaria leaf blight, a fungal pathogen that causes leaf necrosis and plant death in carrots (Pryor and Strandberg 2001). Distributions of imputed data matched those expected from observed data when accounting for environmental variation (Figures S5 and S6 in File S1).

### Additive and nonadditive gene action contributed to observed phenotypes

**Figure 2:**
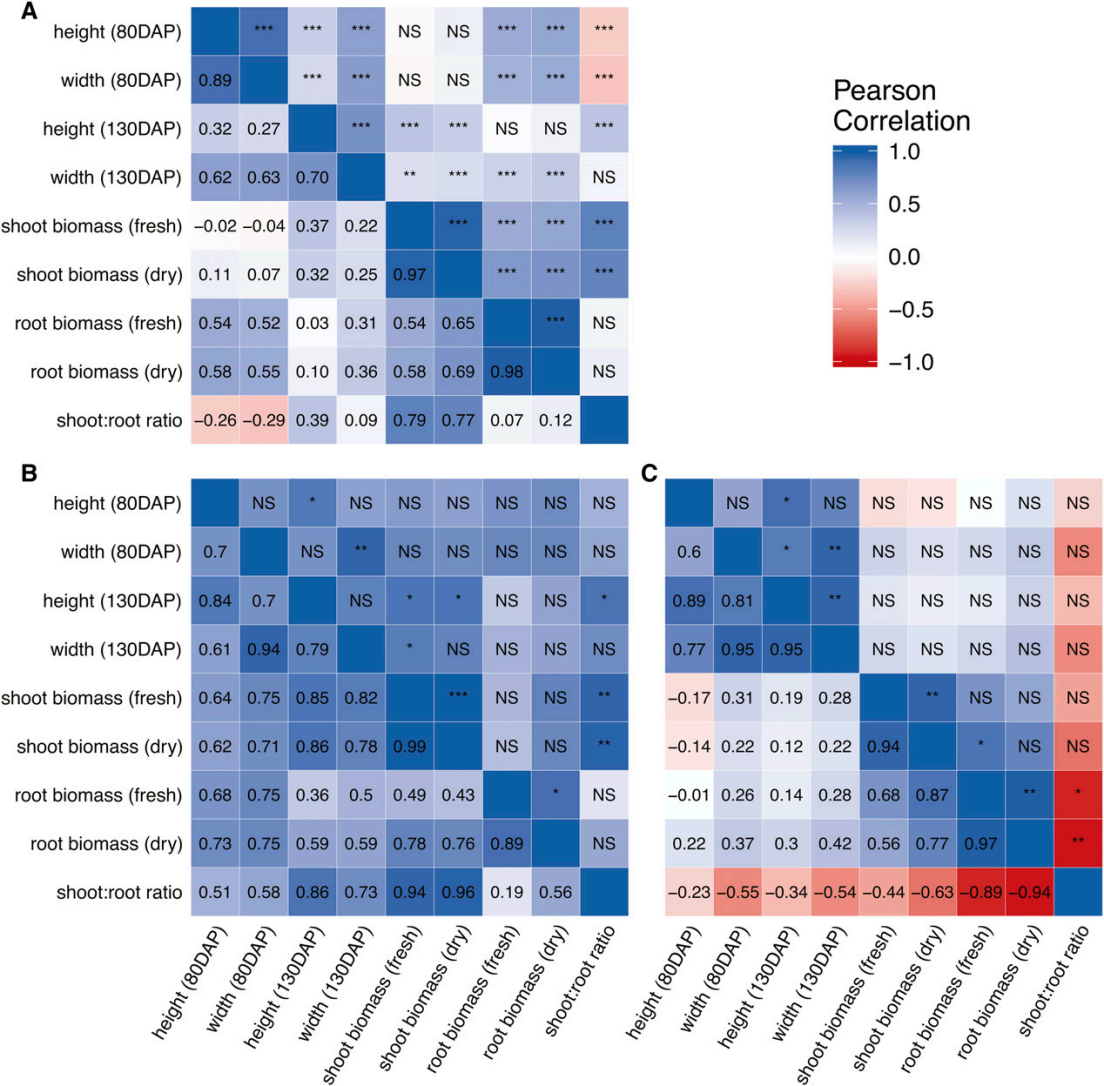
Pearson's correlation coefficients (lower diagonal) and significance (upper diagonal) among carrot growth traits measured in this study for raw phenotypic observations in (A), estimates of additive effects from BayesDiallel in (B) and estimates of inbred deviations from BayesDiallel in (C). Significance codes: ***P ≤ 0.001, **P ≤ 0.01, *P ≤ 0.05. NS, not significant.

**Figure 3:**
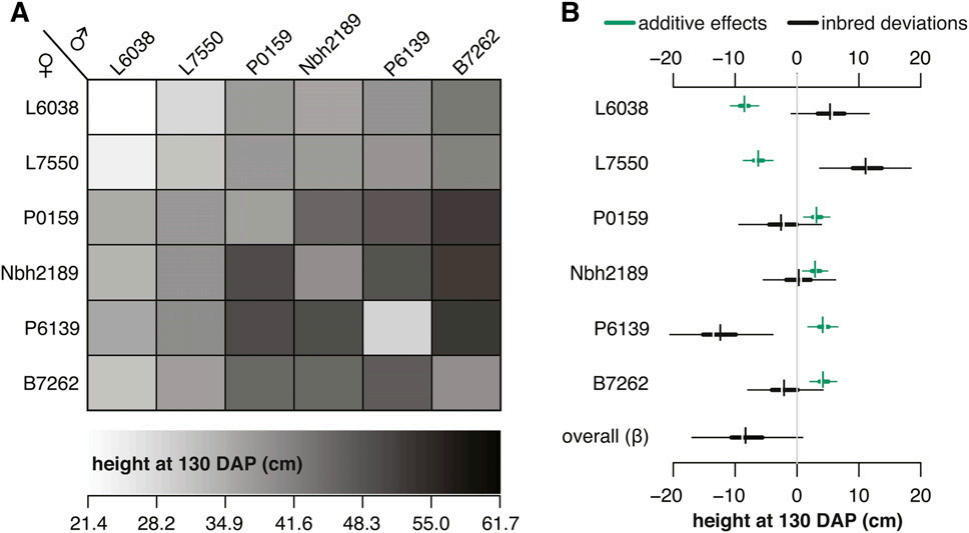
Diallel effects for carrot canopy height at 130d after planting. (A) Predicted means from BayesDiallel. Shading indicates height on a scale from 21.4cm (lighter) to 61.7cm (darker). (B) HPD intervals of parent-specific additive effects, inbred deviations, and overall inbred penalty (β). For each effect, thin and thick horizontal lines show the 95 and 50% HPD intervals, respectively, with breaks indicating the posterior median and short vertical bars the posterior mean.

Most phenotypes were positively correlated based on Pearson’s *r* and significant at *α* = 0.001, with the exception of the ratio for shoot:root biomass with both canopy height and width at 80 DAP (Figure 2A). Griffing’s analysis revealed significant genotypic differences for all phenotypes (Table 2), which are also reflected in the posterior predicted means from BayesDiallel (Figure 3A and File S1). For all traits, both GCA and SCA contributed significantly to the observed genotypic variation, suggesting that both additive and nonadditive effects are important (Table 2). Additionally, Baker’s ratio suggested a larger influence of GCA variance (*i.e.*, additive effects) compared to SCA variance (*i.e.*, dominance and/or epistatic effects) for the phenotypes measured (Table 2). This is reaffirmed by the results from BayesDiallel, in which the highest posterior density intervals for parent-specific inbred effects were more dispersed than for additive effects (Figure 3B and File S1).

**Table 2:** Griffing’s method I, model I ANOVA mean square values for carrot growth traits, including canopy height and width, shoot biomass, root biomass, and the ratio of shoot:root biomass

		Height		Width		Shoot Biomass*^a^*		Root Biomass*^a^*		Shoot:Root Ratio*^a^*
Source	df	80 DAP	130 DAP	80 DAP	130 DAP	Fresh	Dry	Fresh	Dry	
Genotype (G)	35	331.34***	489.3***	353.57***	433.77***	1.8***	2.13***	0.76***	0.91***	0.24***
GCA	5	516.06***	1488.95***	363.35***	1024.37***	7.27***	8.55***	1.13***	1.74***	1.27***
SCA	15	393.69***	376.33***	362.43***	435.66***	0.95***	1.21***	1.11***	1.3***	0.06***
Reciprocal	15	207.42***	269.07***	341.46***	235.02***	0.83***	0.91***	0.28***	0.24**	0.07***
Location (E)	2	10794.6***	268.7**	11668.22***	944.08***	0.11	2.06***	17.8***	19.71***	0.27***
G × E	70	25.91*	68.48***	55.6	64.74	0.26***	0.28**	0.12	0.12	0.04***
GCA × E	10	39.72*	134.49***	55.63	110.75*	0.45***	0.4**	0.21*	0.2	0.08***
SCA × E	30	25.26*	43.07	36.82	50.95	0.26**	0.31**	0.14	0.13	0.04***
Reciprocal × E	30	21.96	71.88**	74.37	63.2	0.19*	0.2	0.09	0.09	0.02
rep(E)	3	24	12.49	56.44	106.03	0.1	0.12	0.14	0.15	0.01
Error	105	15.38	32.14	45.39	53.17	0.12	0.14	0.09	0.1	0.01
Baker’s ratio*^b^*										
Method I		0.72	0.89	0.67	0.82	0.94	0.93	0.67	0.73	0.98
Method III		0.95	0.99	0.90	0.97	0.96	0.96	0.88	0.93	0.96

*** *P* ≤ 0.001, ** *P* ≤ 0.01, * *P* ≤ 0.05.

a Natural log transformation; measured at harvest (130 DAP).

b Baker's ratio of GCA to SCA variance, calculated as 2MSGCA : 2MSGCA + MSSCA, is reported for both Method I (F1s, reciprocal F1s, and parents) and Method III (F1s and reciprocal F1s only).

For effect estimates from BayesDiallel, significant correlations were observed among additive effects for similar phenotypes, *e.g.*, between canopy height at 80 and 130 DAP (*r* = 0.84, *P* ≤ 0.05) (Figure 2B). Additive effects for canopy height at 130 DAP were also correlated with those for shoot biomass (*r* = 0.86, *P* ≤ 0.05) and for shoot:root ratio (*r* = 0.86, *P* ≤ 0.05) (Figure 2B). Although not always significant, inbred effects had high correlations (*r* > 0.5) among canopy height and width, both 80 and 130 DAP, and among shoot and root biomass (Figure 2C). Interestingly, inbred effects for all phenotypes were negatively correlated with shoot:root ratio, of which only the correlation with root biomass was significant (*r* = −0.94, *P* ≤ 0.01) (Figure 2C).

### Inbred deviations differed across genetic backgrounds

Results from Griffing’s analysis indicated that the observed phenotypes were largely under additive genetic control (Table 2), which is also reflected in the posterior predicted means and highest posterior density intervals from BayesDiallel (File S1). However, the BayesDiallel model also captured notable parent-specific inbred deviations. These effects are illustrated by the BayesDiallel results for canopy height at 130 DAP, for which inbred lines were an average of 8.3 cm shorter than their hybrid counterparts (overall inbred effect, $\beta_{\text{inbred}},$ in Figure 3). Additionally, the intensity of inbred effects also varied across genetic background (Figure 3 and File S1). Relative to heterozygotes, line L6038 had a net reduction in canopy height of 2.9 cm ($\beta_{\text{inbred}}+b_{j\left[1\right]}$), while line P6139 had a net 23.4 cm reduction in canopy height ($\beta_{\text{inbred}}+b_{j\left[5\right]}$) (Figure 3).

### Identification of superior parents, hybrids, and testers for applied breeding

GCA estimates from Griffing’s analysis were compared to determine the relative performance of each parent (Table 3). Parent L6038 had negative and significant GCA for all traits except canopy height and width at 80 DAP. Low and significant GCA was also observed in parent L7550 for height (130 DAP) and the ratio of shoot:root biomass. For canopy height, parents with positive and significant GCA included Nbh2189 (130 DAP), P6139 (80 and 130 DAP), and B7262 (130 DAP). Parent Nbh2189 was the only inbred with significant and positive GCA for canopy width (130 DAP). Parents P0159 and B7262 had high and significant GCA for both shoot biomass and the ratio of shoot:root biomass. Positive and significant GCA for root biomass was only observed for parent P0159.

**Table 3:** Pooled estimates of GCA for carrot growth traits combined across all growing environments

	Height		Width		Biomass*^a^*		
Parent	80 DAP	130 DAP	80 DAP	130 DAP	Shoot	Root	Shoot:Root Ratio
L6038	−1.39	−6.5***	0.09	−3.5**	−0.53***	−0.19*	−0.19***
L7550	−2.83	−4.94***	−0.63	−2.23	−0.15	−0.03	−0.08**
P0159	0.85	1.43	−1.56	−1.41	0.39***	0.27***	0.1***
Nbh2189	2.43	3.62**	3.15	7.13***	0.11	0.06	0.03
P6139	3.52*	3.87**	1.86	0.53	−0.15	−0.07	−0.03
B7262	−2.58	2.52*	−2.92	−0.53	0.33***	−0.03	0.18***
Grand Mean	32.27	45.77	43.57	53.74	1.55	2.55	0.6

*** *P* ≤ 0.001, **P ≤ 0.01, *P ≤ 0.05.

a Natural log transformation; dry weight as measured at harvest (130 DAP).

Although the estimation methods were different, GCA estimates largely agree with the additive effects estimated from BayesDiallel, which provided similar rankings based on posterior predicted means (Figure 3A and File S1) and HPD intervals (Figure 3B and File S1). For canopy height (130 DAP), hybrids with parents L6038 and L7750 were, on average, ∼7.3 cm shorter, while hybrids with parents P6139 and B7262 were an average of 4.2 cm taller (Figure 3). The posterior predicted means for canopy height (130 DAP) also demonstrate relatively higher values for hybrids with parents Nbh2189, P6139, and B7262, as well as lower values for hybrids with parents L6038 and L7550 (Figure 3).

**Figure 4:**
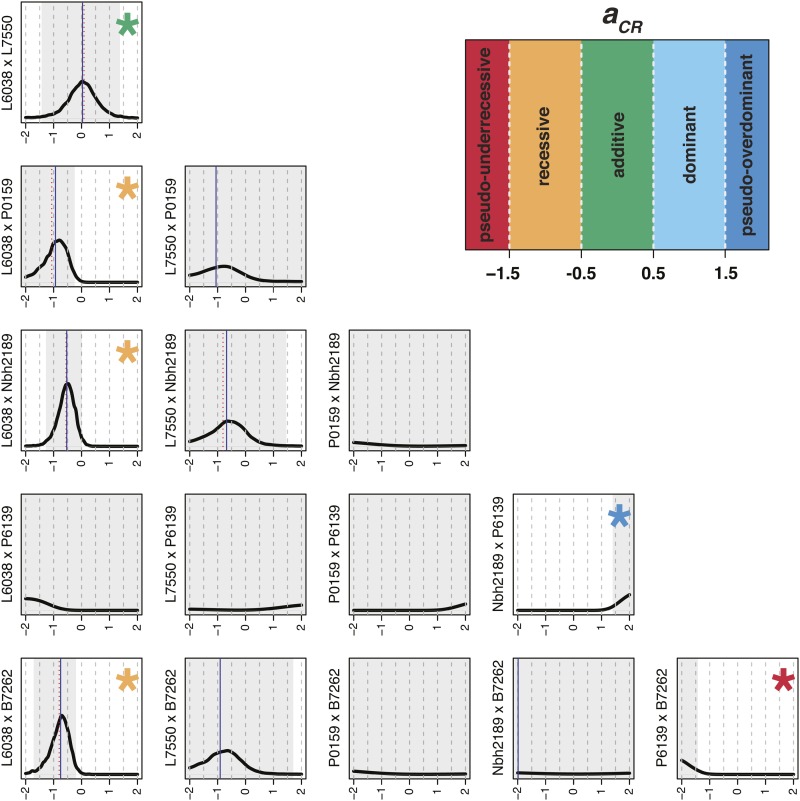
Posterior distributions of the degree of dominance (acr) for canopy height at 130d after planting. Posterior means within the interval are indicated by red dotted lines and medians by blue solid lines, with 95% central quantiles shown in gray. A signal was considered strong if the mean, median, and the majority of the posterior distribution fell within the specified ranges: pseudo-under-recessive (−∞,−1.5), recessive (−1.5,−0.5), additive (−0.5,0.5), dominant (0.5,1.5), pseudo-overdominant (1.5,∞). Asterisks indicate a high posterior probability that, in aggregate, genetic effects of parent A influencing canopy height at 130 DAP are pseudo-under-recessive, recessive, additive, dominant, or pseudo-overdominant to those of parent B (*e.g.*, there is a high posterior probability that the genetic effects of parent L6038 influencing canopy height at 130 DAP are recessive to those of parent B7262).

For Griffing’s analysis, SCA effects were identified as crosses that performed better or worse than expected based on the GCA values of the contributing parents (Table 4). Hybrid Nbh2189 × P6139 had high SCA for both height and width (80 and 130 DAP). For shoot biomass, the largest SCA was observed in hybrid Nbh2189 × B7262. Hybrids with high SCA for root biomass included L7550 × B7262, P0159 × Nbh2189, and Nbh2189 × B7262. No significant positive effects were observed for shoot:root ratio. These results are consistent with posterior distributions for the degree of dominance, with pseudo-overdominance observed in hybrid Nbh2189 × P6139 for canopy height (80 and 130 DAP; Figure 4 and Figure S11 in File S1) and in hybrids L7550 × B7262 and Nbh2189 × B7262 for root biomass (Figures S47 and S53 in File S1). Signals of dominance and pseudo-overdominance were also detected for canopy width at 130 DAP in hybrids L7550 × P6139 and Nbh2189 × B7262 (Figure S29 in File S1). Notably, parents L6038 and P0159 also showed signals of pseudo-overdominance for root biomass when crossed to B7262 (Figures S47 and S53 in File S1).

**Table 4:** Pooled estimates of SCA for carrot growth traits combined across all growing environments

	Height		Width		Biomass*^a^*		
	80 DAP	130 DAP	80 DAP	130 DAP	Shoot	Root	Shoot:Root Ratio
F1 hybrids							
L6038 × L7550	−0.53	−0.75	−1.4	−2.27	0.1	0	0.06
L6038 × P0159	1.05	1.83	1.82	2.86	0.12	0.17	0.05
L6038 × Nbh2189	1.01	−2.03	0.45	−2.62	−0.24	−0.06	−0.1*
L6038 × P6139	1.66	0.96	−0.8	2.43	0.21	0.15	0.06
L6038 × B7262	2.05	0.95	4.62	2.67	−0.06	0.09	−0.04
L7550 × P0159	−1.08	−1.75	−1.91	0.61	0.1	0.19	−0.01
L7550 × Nbh2189	0.48	−0.63	−1.97	−0.28	0.28	0.04	0.09
L7550 × P6139	0.34	0.68	0.01	−0.76	−0.05	−0.03	0.01
L7550 × B7262	−3.43	−2.92	0.96	−1.45	0.33*	0.39***	0
P0159 × Nbh2189	1.46	1.77	1.38	0.23	0.09	0.28**	−0.05
P0159 × P6139	4.04*	4.99*	3.95	1.24	−0.14	−0.04	−0.05
P0159 × B7262	4.39**	2.93	4.28	4.79*	−0.12	−0.09	−0.08
Nbh2189 × P6139	5.57***	6.26**	7.01**	7.2**	0.17	0.12	0.05
Nbh2189 × B7262	−5.45**	0.6	−1.66	1.7	0.54***	0.3**	0.08
P6139 × B7262	2.32	3.17	2.73	5*	−0.22	−0.04	−0.09*
Parental selfs							
L6038	−5.24*	−1.97	−4.58	−3.73	−0.16	−0.33*	−0.03
L7550	2.35	4.31	1.74	3.14	−0.44*	−0.38**	−0.08
P0159	−13.12***	−12.06***	−10.72**	−11.63**	−0.43*	−0.83***	0.1
Nbh2189	−6.40**	−4.48	−9.11**	−8.38*	−0.47*	−0.52***	0.01
P6139	−18.85***	−19.93***	−16.83***	−19.55***	−0.15	−0.34*	−0.01
B7262	−3.42	−6.22	−8.6*	−10.37**	−0.81***	−0.88***	0.05

*** *P* ≤ 0.001, **P ≤ 0.01, *P ≤ 0.05.

a Natural log transformation; dry weight as measured at harvest (130 DAP).

In addition to pseudo-overdominance, other signals of nonadditivity were detected based on the degree of dominance. Several parents expressed dominance when crossed to P6139, including L7550 for canopy width at 80 DAP (Figure S23 in File S1), Nbh2189 for canopy width at 130 DAP (Figure S29 in File S1), and P0159 for fresh shoot biomass (Figure S35 in File S1). Parent L6038 was generally recessive or pseudo-under-recessive for all traits except root biomass, agreeing with previous observations of low GCA (File S1). Similarly, parent L7550 was either recessive or pseudo-under-recessive when crossed to P0159, Nbh2189, and B7262 for shoot biomass (Figures S35 and S41 in File S1), and to Nbh2189 for shoot:root ratio (Figure S59 in File S1). In contrast with the other phenotypes, strong signals of additivity were detected for shoot:root ratio, particularly for crosses with parents P0159 and B7262 (Figure S59 in File S1).

The best tester for each trait was both discriminating (*i.e.*, able to rank the other parental lines by combining ability) and representative (*i.e.*, minimal SCA effects) based on biplot analysis. Line B7262 was the most discriminating parent in hybrid combination for height and width, both 80 and 130 DAP (Figures S7, S13, S19, and S25 in File S1), suggesting it can serve as a valuable tester to rank the combining ability of other parental lines for shoot height and width in carrot. The best testers for other traits included line P0159 for shoot biomass (Figures S31 and S37 in File S1), line P6139 for root biomass (Figures S43 and S49 in File S1), and line L6038 for shoot:root ratio (Figure S55 in File S1).

### Influence of reciprocal cross effects and genotype-by-environment interactions

Highly significant reciprocal effects were detected for all traits in Griffing’s analysis (Tables 2 and 5), suggesting parent-of-origin influences phenotypic expression. For increasing height and width (80 and 130 DAP), lines L6038 and P0159 tended to perform best as female parents and lines L7550 and B7262 tended to perform best as male parents. Significant increases were also observed for shoot biomass, root biomass, and shoot:root ratio when line L7550 was used as a female parent and when lines P0159 and Nbh2189 were used as male parents.

**Table 5:** Pooled estimates of reciprocal cross effects for carrot growth traits over all growing environments

	Height		Width		Biomass*^a^*		
F1 Hybrids	80 DAP	130 DAP	80 DAP	130 DAP	Shoot	Root	Shoot:Root Ratio
L6038 × L7550	4***	4.35**	4.51*	5.25**	−0.1	−0.08	−0.02
L6038 × P0159	−0.91	−2.21	−3.37	−2.62	0.19	0.12	0.05
L6038 × Nbh2189	2.97**	2.43	2.22	3.74*	0.12	0.05	0.03
L6038 × P6139	2.96**	2.76	3.01	3.61*	−0.01	0.1	−0.03
L6038 × B7262	7.18***	9.93***	8.75***	7.14***	−0.15	−0.06	−0.06
L7550 × P0159	−2.89**	−1.61	−3.44	0.32	0.58***	0.28***	0.14***
L7550 × Nbh2189	−7.17***	−4.3	−9.45***	−5.42*	0.77***	0.36***	0.22***
L7550 × P6139	−4.58***	−1.5	−7.17***	−4.13*	0.03	0.04	0.01
L7550 × B7262	−0.24	2.46	1.4	1.04	0.11	0.15	−0.02
P0159 × Nbh2189	−1.5	−6.63***	−3.29	−2.81	0.04	0.04	0.01
P0159 × P6139	−4.72***	−2.96	−6.78***	−4.24*	−0.01	−0.01	0.01
P0159 × B7262	3.18**	6.74***	1.31	4.09*	−0.07	−0.03	0.01
Nbh2189 × P6139	0.81	−1.92	−0.97	−0.82	0	0.07	−0.02
Nbh2189 × B7262	6.14***	7.35**	7.15**	8.75***	−0.28*	−0.09	−0.08*
P6139 × B7262	3.96***	2.58	5.39**	1.71	−0.01	−0.06	0

*** *P* ≤ 0.001, **P ≤ 0.01, *P ≤ 0.05.

a Natural log transformation; dry weight as measured at harvest (130 DAP).

**Figure 5:**
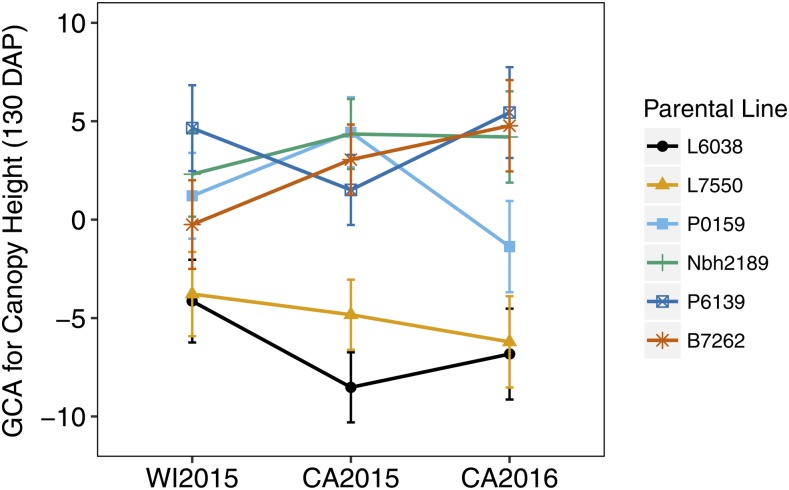
Interaction of GCA and location (WI2015, CA2015, CA2016) for canopy height at 130 DAP.

Based on Griffing’s analysis, genotype by environment interaction (G × E) was significant for canopy height (80 and 130 DAP), shoot biomass (fresh and dry), and shoot:root ratio (Table 2). For corresponding traits with significant GCA × E, SCA × E, and Reciprocal × E interactions, estimates and nonparametric correlations among environments (Spearman’s ρ) for each location are provided in Tables S2–S23 in File S1. Significant GCA × E interactions were observed for canopy height (80 and 130 DAP), shoot biomass, and shoot:root ratio. For canopy height (130 DAP), GCA ranked consistently negative across environments for parents L6038 and L7550 (Figure 5 and Table S6 in File S1). Parent P6139 had positive GCA in all environments, but effects were only significant for the WI2015 and CA2016 locations (Table S6 in File S1). The performance of parents P0159 and B7262 was notably inconsistent and fluctuated between negative and positive values of GCA (Figure 5). SCA × E interactions were significant for height (80 DAP), shoot biomass, and shoot:root ratio, but it was still possible to identify consistently high performing hybrids across environments using hybrid rankings from BayesDiallel (Figures S12, S36, S42, and S60 in File S1). Similarly, significant Reciprocal × E interactions were observed for canopy height (130 DAP) and fresh shoot biomass. Differences across replications within a location were not significant.

**Figure 6:**
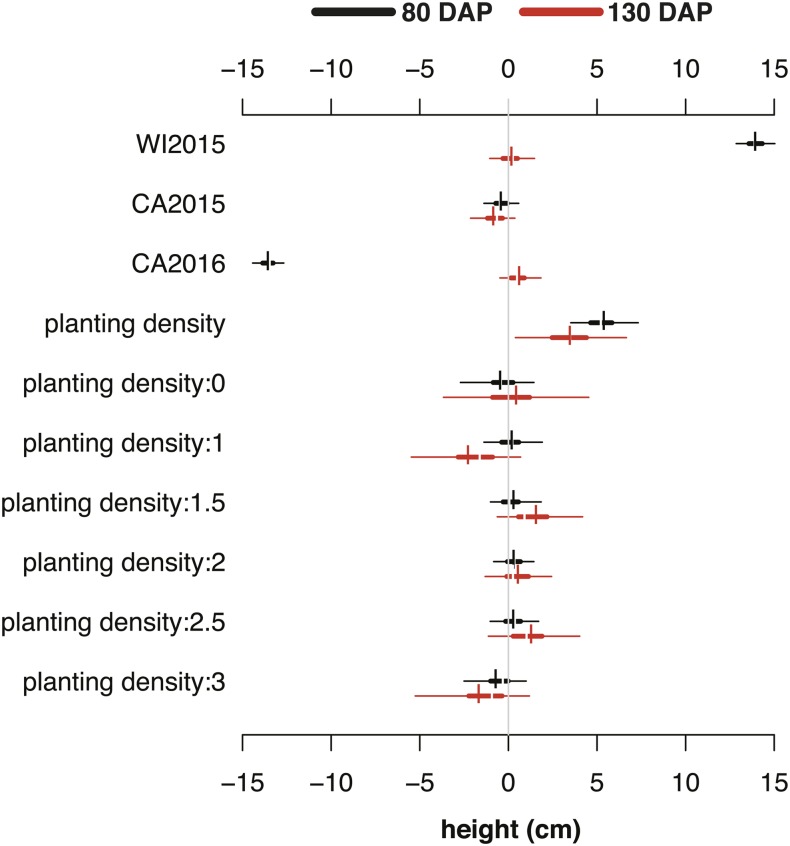
HPD intervals of location and planting density (stand; 1–3 scale, 1 = low, 3 = high) for canopy height at 80 DAP (black) and 130 DAP (red). For each effect, thin and thick horizontal bars show the 95 and 50% HPD intervals, respectively, with vertical breaks indicating the posterior median and short vertical bars the posterior mean.

**Figure 7:**
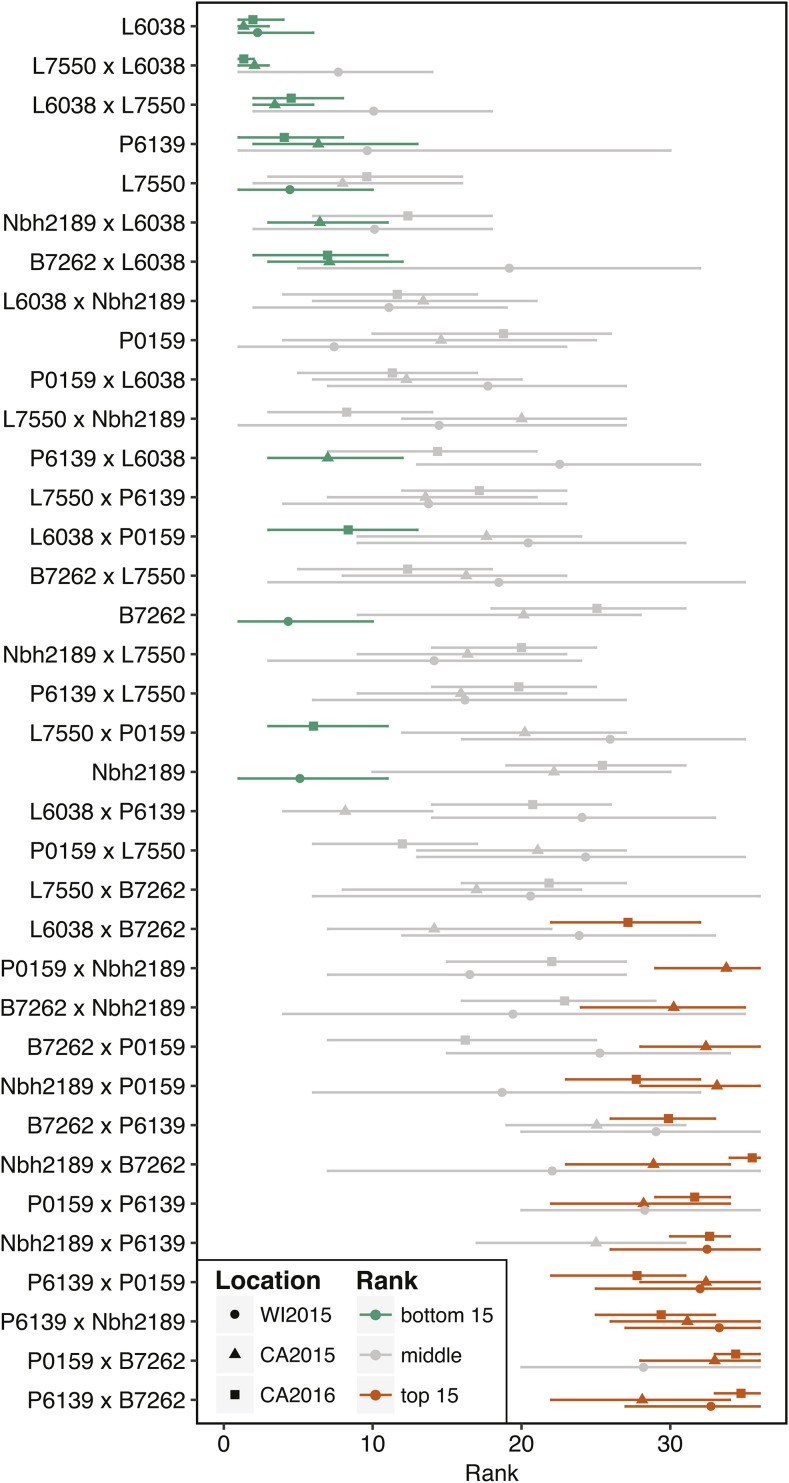
Mean values and 95% HPD intervals of hybrid rankings for canopy height (130 DAP) in a six-parent carrot diallel. Hybrids with intervals in the bottom 15 are shown in green, and hybrids with intervals in the top 15 are shown in orange.

Effects estimated in BayesDiallel included planting density (fixed and random) and location (random) (File S1). On average, planting density increased plant height in a mostly linear fashion, with a greater effect at 80 DAP (5.4 cm) compared to 130 DAP (3.5 cm) (Figure 6). Similarly, location had a greater influence on height at 80 DAP than at 130 DAP, with the highest mean in the WI2015 season and the lowest mean in the CA2016 season (Figure 6). Stratified analysis by location also allowed estimation of G × E by providing rankings of crosses in each growing environment (Figure 7). For canopy height (130 DAP), hybrids P6139 × P0159, P6139 × Nbh2189, and P6139 × B7262 consistently ranked among the tallest in all locations (Figure 7). Although rankings had greater uncertainty in the WI2015 environment, it was generally possible to distinguish consistently high and low ranking hybrids across environments for all phenotypes.

### Genetic architecture varied across traits and over developmental time

**Figure 8:**
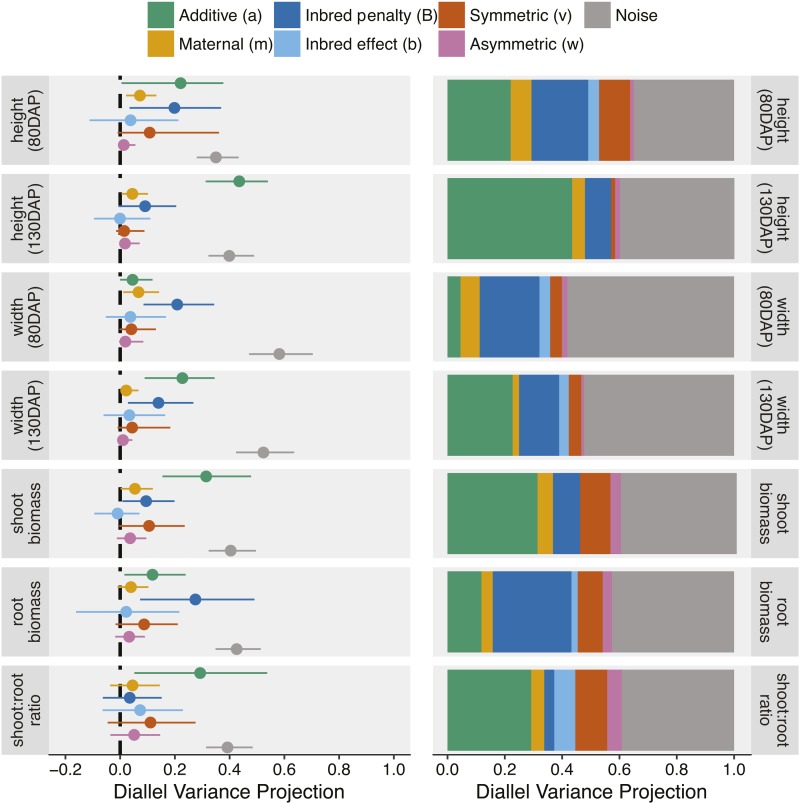
Diallel variance projections characterizing the genetic architecture for each trait, including additive (a), maternal (m), overall inbred penalty (B), parent-specific inbred deviations (b), cross-specific symmetric (v), and cross-specific asymmetric (w) effect classes. Left: mean and 95% credibility intervals of effect classes for each trait. Right: Mean values showing overall genetic architecture.

Although most correlations among phenotypes ranged from moderate (*r* > 0.3) to high (*r* > 0.5) (Figure 2A), genetic architecture varied substantially by trait and across developmental time (Figure 8). Similarly, estimates of Baker’s ratio using both Method I (with parents) and Method III (without parents) suggest the influence of SCA on canopy height and width is larger early in the growing season (80 DAP) compared to the end of the season (130 DAP), although the magnitude of this difference is diminished when using Method III (Table 2). Interestingly, and regardless of estimation method, the lowest value for Baker’s ratio was observed for root biomass, suggesting this trait has a higher influence of SCA effects relative to the other phenotypes measured (Table 2). Results from BayesDiallel reveal a similar relationship, with the overall inbred penalty $\left(\beta_{\text{inbred}}\right)$ explaining more or similar amounts of variation compared to additive effects for midseason height, midseason width, and root biomass (Figure 8 and Table 6).

**Table 6:** Diallel VarP for carrot growth traits

	Height		Width		Biomass*^a^*		
Diallel Inheritance Class	80 DAP	130 DAP	80 DAP	130 DAP	Shoot	Root	Shoot:Root Ratio
Additive (a)	22.07 (0.49,37.72)	43.49 (31.24,53.98)	4.56 (−0.04,11.83)	22.75 (8.96,34.53)	31.43 (15.47,47.83)	11.84 (1.55,23.9)	29.21 (5.18,53.73)
Parent of origin (m)	7.24 (2.15,13.23)	4.48 (0.49,10.19)	6.67 (1.12,14.21)	2.22 (0.14,6.66)	5.37 (0.14,11.93)	3.95 (−0.98,10.31)	4.55 (−3.68,14.49)
Overall inbred (B)	19.8 (3.43,36.9)	9.1 (−0.57,20.49)	20.87 (8.54,34.36)	13.98 (2.85,26.76)	9.44 (0.82,19.86)	27.48 (7.3,49.06)	3.51 (−6.33,15.18)
Inbred (b)	3.79 (−11.2,21.29)	−0.08 (−9.57,11.05)	3.74 (−5.23,16.75)	3.37 (−6.07,16.45)	−0.91 (−9.46,7.08)	2.17 (−16.12,21.62)	7.34 (−6.41,22.94)
Symmetric (v)	10.82 (−0.93,36.13)	1.44 (−1.49,8.87)	4.09 (−0.24,13.07)	4.36 (−0.95,18.33)	10.6 (−0.34,23.6)	8.73 (−1.67,21.04)	11.12 (−4.57,27.55)
Asymmetric (w)	1.32 (−0.46,5.57)	1.73 (−0.2,7.15)	1.92 (−0.38,8.46)	1 (−0.1,4.46)	3.68 (−1.19,9.57)	3.27 (−1.8,9.05)	5.08 (−3.58,14.62)
Total variance explained	65.03 (56.69,72.02)	60.15 (51.04,67.74)	41.85 (29.6,52.9)	47.69 (36.41,57.67)	59.62 (50.38,67.66)	57.43 (48.6,65.16)	60.81 (51.58,68.61)
Unexplained variance	34.97 (27.98,43.31)	39.85 (32.26,48.96)	58.15 (47.1,70.4)	52.31 (42.33,63.59)	40.38 (32.34,49.62)	42.57 (34.84,51.4)	39.19 (31.39,48.42)

Under each trait (column) is listed the predicted percentage and 95% credibility intervals of variance that would be attributable to each class of effect. Predictions are conditional on a future complete diallel composed of the same parental lines.

a Natural log transformation; dry weight as measured at harvest (130 DAP).

As described by Crowley *et al.* (2014), the variance projection of the additive diallel inheritance class, VarP[a], can be likened to repeatability. Traits with significant additive effects included canopy height at 80 DAP (VarP[a] = 0.22), canopy height at 130 DAP (0.43), canopy width at 130 DAP (0.23), shoot biomass (0.31), root biomass (0.12), and shoot:root ratio (0.29) (Figure 8 and Table 6). The influence of nonadditive variation was largely due to the overall inbred penalty, which contributed significantly to canopy height at 80 DAP (VarP[B] = 0.20), canopy width at 80 DAP (0.21) and at 130 DAP (0.14), shoot biomass (0.09), and root biomass (0.27) (Figure 8 and Table 6). However, parent-specific inbred effects, cross-specific symmetric effects, and cross-specific asymmetric effects did not contribute significantly to the predicted phenotypes (Figure 8 and Table 6). While parent-of-origin is not a genetic effect, it did explain variation for canopy height at 80 DAP (Var[m] = 0.07) and 130 DAP (0.04), canopy width at 80 DAP (0.07) and 130 DAP (0.02), and shoot biomass (0.05) (Figure 8 and Table 6).

## Discussion

### Primary gene action

In this study, we estimated genetic, parent-of-origin, and environmental effects on carrot growth traits for six carrot inbred lines, and their combinations, in a 6 × 6 diallel framework. Significant genetic variation contributed to all carrot growth attributes, suggesting that there is potential to improve these traits in carrot.

Apart from canopy width at 80 DAP, all phenotypes had a measurable proportion of heritable variation, as evidenced by the presence of nonzero additive effects in the diallel variance projection. Traits with high additivity included canopy height (130 DAP) and shoot biomass, both of which are well documented as highly heritable polygenic traits that play a fundamental role in plant fitness and adaptation (Khush 2001; Meyer *et al.* 2004; Peiffer *et al.* 2014). High additivity was also observed for the ratio of shoot:root biomass, which had strong signals of additivity based on the degree of dominance, $a_{CR}.$

For the parental lines in this study, we observed varying sensitivity to inbreeding, which could be due to genetic divergence and/or differing levels of prior inbreeding (East 1936; Birchler *et al.* 2003). This matches expectations based on the biological constraints of outcrossing in carrot, which has putative susceptibility to inbreeding depression (Simon 2000). Consequently, hybrid vigor was evident for root biomass, which had relatively high proportions of nonadditive genetic variation, significant estimates of an inbred penalty, and signals of pseudo-overdominance (Figure 8 and Figures S47 and S53 in File S1). This result coincides with widespread evidence of heterosis in plants, whereby hybrids demonstrate increased developmental speed and greater biomass acquisition relative to their inbred parents (Shull 1908; East 1936; Birchler *et al.* 2003; Meyer *et al.* 2004), and agrees with previous observations of hybrid vigor for root weight in carrot (Simon *et al.* 1982; Duan *et al.* 1996; Simon and Strandberg 1998; Suh *et al.* 1999; Jagosz 2011, 2012).

### Breeding strategies

With discovery of CMS systems in carrot (Welch and Grimball 1947), breeding strategies transitioned from selection in open-pollinated populations to an inbred-hybrid system, thereby improving crop uniformity and vigor (Peterson and Simon 1986). We expect that traits with significant overall inbred effects, such as canopy height (80 DAP), canopy width (80 DAP), and root biomass, will be responsive to commonly used hybrid breeding strategies in carrot, such as reciprocal recurrent selection. Alternatively, selection for traits with high additivity, such as canopy height (130 DAP), canopy width (130 DAP), shoot biomass, and shoot:root ratio may allow more rapid genetic gain while indirectly selecting for positively correlated traits under nonadditive control. For all phenotypes in this study, we also identified promising parental lines and hybrids for use in applied breeding. Line B7262 was especially notable as a favorable tester for canopy height and width, which may be useful to assess the combining abilities of carrot germplasm at midseason and at harvest.

Accounting for G × E is especially important in biennial crops like carrot, as breeding programs rely on winter nurseries to achieve an annual breeding cycle (Simon *et al.* 2008). We conducted trials in CA and WI, which are two of the leading carrot production regions and representative of common, but contrasting, breeding environments. In general, significant G × E interactions did not affect the ability to identify high and low rankings among parents and hybrids. Thus, we anticipate that environmental differences are important, but should have a minimal impact on selection efforts.

### Source–sink relationships

Biomass partitioning between the shoot and root is a major consideration in carrot breeding, and has been extensively studied, both in a breeding and an ecological context (Barnes 1979; Currah and Thomas 1979; Hole *et al.* 1983; Thomas *et al.* 1983; Hole and Dearman 1991). The ideotype for carrot shoot growth is rapid initial growth that plateaus following canopy closure, simultaneously reducing the critical weed free period and promoting taproot development. Equally important is avoiding growth habits with large, dense canopies, which foster a microclimate that is conducive to the development of foliar diseases (Simon *et al.* 2008).

Consistent with findings by Hole *et al.* (1983), we found a strong linear relationship between the log transforms of shoot and root biomass (*r* = 0.69, *P* < 0.001). However, the ratio of shoot:root biomass had a wide range across parents (0.23–0.64), providing evidence that high shoot biomass is not necessary to produce roots with high biomass, and vice versa.

Previous work has demonstrated rapid and early acquisition of dry matter in carrot storage roots, with the taproot constituting 42% of the plant dry weight at 67 d after sowing (Benjamin and Wren 1978). Interestingly, our results demonstrate that canopy height and width at 80 DAP were negatively correlated with the ratio of shoot:root biomass (*r* = −0.26 and −0.29, respectively, *P* < 0.001) and positively correlated with root biomass at harvest (*r* = 0.58 and 0.55, respectively, *P* < 0.001). Conversely, canopy height at harvest (130 DAP) was positively correlated with shoot:root ratio (*r* = 0.39, *P* < 0.001) and not significantly correlated with root biomass (*r* = 0.10, *P* = 0.18). This suggests that early shoot growth is important for root biomass accumulation and, in combination with observations of pseudo-overdominance and significant inbred effects, agrees with previous conclusions that these traits may be subject to hybrid vigor.

### Method of analysis

When applied appropriately, traditional diallel analysis provides valuable information on combining abilities for parental lines and the underlying gene action for complex traits (Griffing 1956b). However, the benefits of diallel mating designs are often overshadowed by practical and theoretical constraints of the analysis. The use of hierarchical modeling, and, in particular, the use of a Bayesian MCMC approach as used here, confers several advantages, including robust handling of imbalanced data, which is especially important for crops with poor seed set or limited availability of inbred lines; better support intervals for ranks and variances; straightforward inference of potentially complex functions of estimated parameters (such as the degree of dominance), and, as a consequence of these, improved biological interpretability (Greenberg *et al.* 2010; Lenarcic *et al.* 2012). For this study, we chose to present two complementary analyses of diallel data: a traditional analysis using Griffing’s Model I, combined in this case with multiple imputation, and a modern analysis using BayesDiallel. The advantage of this comparison is the presentation of results from BayesDiallel, which is not currently used in plant breeding and may be intimidating to researchers unfamiliar with Bayesian approaches, in the context of Griffing’s analysis, which is relatable and familiar to plant breeders. Both methods provided similar conclusions regarding primary gene action, parental rankings, and hybrid performance.

Of relevance in this experiment was robustness to data imbalance, which is a pervasive challenge in field experiments. Although this was not a problem for BayesDiallel, it was a substantial challenge when applying Griffing’s model, for which balance is required. To ensure valid application of Griffing’s model under data imbalance, we framed the imbalance as a missing data problem, and addressed this using multiple imputation, which produces a set of plausible values to replace missing information (Rubin 1987). This approach may be useful for researchers interested in performing traditional diallel analysis with fixed genotypes. Imputation has advantages over alternative methods to address missing data in diallel experiments in that it is relatively simple to implement, makes use of all available data for a given trait, and replaces missing data with plausible estimates to avoid reductions in sample size. However, there are several caveats and compromises regarding multiple imputation, namely, that there are inadequate or vague diagnostics and, although simple in principle, methods to pool multi-factor ANOVA results are often vague, or are not widely accessible (van Ginkel and Kroonenberg 2014; Grund *et al.* 2016). In this study, we demonstrate the application of existing, relatively straightforward, methods to pool results for diallel analysis across multiple environments.

A notable advantage of BayesDiallel was the option to add covariates for location and planting density as random and fixed effects. Posterior distributions for location matched expectations based on observed data, with higher means observed in WI2015 compared to CA2015 and CA2016. The inclusion of planting density was especially advantageous and matched expectations from previous studies, which demonstrated significant effects of planting density on canopy height and biomass partitioning in carrot (Bleasdale 1967; Benjamin and Sutherland 1992; Li *et al.* 1996; Traka-Mavrona 1996). Of interest in future studies will be further investigation of potential genotype × density interactions.

Precise estimates of repeatability are useful when determining which breeding strategy will result in the most genetic gain. However, traditional repeatability measures based on estimates of variance components typically have wide confidence intervals, and their formulation as a population-level statistic can make meaningful interpretation possible only under restrictive conditions. As an alternative, BayesDiallel’s variance projection (VarP) (Crowley *et al.* 2014) is a more precise, repeatability-like measure with a practical focus: it benefits applied breeding by (1) describing how several inheritance classes will influence future experiments composed of the same parents, and (2) providing a 95% credibility interval as a measure of uncertainty, which affords more flexibility when designing future experiments and estimating potential for genetic gain.

### Conclusions

The rise of sustainable agriculture has tasked breeders to develop cultivars with improved weed competitive ability. Using traditional and modern approaches, we analyzed diallel data to describe the quantitative inheritance of previously uncharacterized traits in carrot, which have been demonstrated to confer improved competitive ability in other crops. However, future trialing for weed competitive ability in carrot will be essential to validate the utility of these traits, to determine the underlying mechanism of competitive ability (*i.e.*, tolerance or suppression), and to assess relative fitness costs (*e.g.*, trade-offs with yield).

Results from this study support applied breeding efforts for carrot shoot growth in numerous ways, most notably through the quantification of inbred performance, the identification of useful tester lines, and the assessment of potential hybrid combinations. Furthermore, the detailed characterization of the inbred parents in this study provides a foundation for the development of a multi-parental advanced generation intercross (MAGIC) population in carrot, which will facilitate future in-depth genetic studies (Huang *et al.* 2015).

Lastly, we demonstrate the utility of the BayesDiallel framework for modeling heritable and nonheritable components of carrot phenotypes. This example will make BayesDiallel more accessible as a resource in the plant breeding community to maximize the potential of diallel experiments, especially in under-resourced crops with limited characterization of heterotic groups and combining abilities.

## Supplementary Material

Supplemental material is available online at www.g3journal.org/lookup/suppl/doi:10.1534/g3.117.300235/-/DC1.

## APPENDIX A

### Degree of Dominance: Statistical Methods

In Comstock and Robinson (1948), a method was described for obtaining a quantitative estimate of the degree of dominance, a, from a simple linear model of phenotypes on genotypes. The model is reproduced in summary below:

p=y+e (A1)

where p is the phenotype, y is the genetic component, and e is the deviation or residual. The paper goes on to describe a variance-component based method of determining the aggregate dominance contribution to the phenotype.^1^

Given a specific locus (in our case, we are considering founder genome contributions rather than loci), two alleles, B and b, can be combined as BB, Bb, or bb in a breeding population, and the phenotypic expectation for hybrids, in the absence of dominance, is given by:

$$y_{Bb}=\left(y_{BB}+y_{bb}\right)/2$$ (A2)

or, equivalently,

$$y_{BB}-y_{Bb}=y_{Bb}-y_{bb}$$ (A3)

Elaborating the notation to consider the case where dominance plays a role, we have:

$$y_{BB}=z+2u$$ (A4)

$$y_{BB}=z+u+au$$ (A5)

$$y_{bb}=z$$ (A6)

We can then rewrite the value of a according to the relationship between $y_{BB},$ $y_{Bb},$ and $y_{bb}$ in the following steps. First, regardless of the value of a, we know that:

$$y_{BB}-y_{bb}=z+2u-\left(z\right)=2u$$ (A7)

Also, we know that:

$$y_{BB}-y_{Bb}=z+2u-\left(z+u+au\right)=u-au=\left(1-a\right)\cdot u$$ (A8)

$$\left(y_{BB}-y_{Bb}\right)/u=1-a$$ (A9)

$$a=1-\left(y_{BB}-y_{Bb}\right)/u$$ (A10)

We substitute the value of u in Equation A7 for the u in Equation A8, giving us the relation:

$$a=1-2\cdot \left(\frac{y_{BB}-y_{Bb}}{y_{BB}-y_{bb}}\;\right)$$ (A11)

We recognize that the quantity on the right, $\left(y_{BB}-y_{Bb}\right)/\left(y_{BB}-y_{bb}\right)$, is equivalent to the definition of the dominance index, D, in Kacser and Burns (1981), which is used in a diallel analysis of dominance in Maurizio *et al.* (2017).

The Comstock-Robinson parameter for dominance^2,3^, referred to as $a_{CR}$ in this article, was furthermore used by Gardner and Lonnquist (1959) to quantify the degree of dominance in genetic crosses of maize. We adapt the classification of degree of dominance in that paper, as follows, with the corresponding values for the Kacser–Burns dominance index, D, given on the right:

**Table:**

Category	Value ($a_{CR}$)	Value ($D_{KB}$)
Pseudo-under-recessivity	a≪−1.0	D≫1.0
Recessivity	a=−1.0	D=1.0
No dominance (additivity)	a=0.0	D=0.5
Dominance	a=1.0	D=0.0
Pseudo-overdominance	a≫1.0	D≪0.0

1 For our purposes, rather than compute estimates of the average or aggregate dominance contribution based on the variance components, which is captured to some extent in the Variance Projection analysis, we use the posterior predictive estimates of phenotypes within the inbred and hybrid groups to generate a posterior distribution of the degree of dominance parameter, $a_{CR}\text{,}$ for all pairings of different inbred parents in the 6 × 6 diallel (*n* = 15 categories).

2 Note that *a* is *a* measure of dominance; it equals zero when dominance is absent and increases in magnitude as *y_Bb_* deviates from the midpoint between *y_BB_* and *y*_bb_**.

3 Therefore if the estimate of *a* is significantly greater than one, it can be concluded that one or more *a* is greater than one, i.e., that there is overdominance of genes at one or more locus.
