# Supplementary material for: Dissecting the Genetic Architecture of Shoot Growth in Carrot (Daucus carota L.) Using a Diallel Mating Design
Source: G3 (Bethesda). 2017 Nov 29;8(2):411–26. doi: 10.1534/g3.117.300235 (PMC5919754; doi:10.1534/g3.117.300235)
Supplement: Supplementary file 1 [file 411FileS1.pdf]

File S1

Turner et al: Supplementary Results

1 Parental lines **4**
1.1 Parental means . . . 5

2 Multiple imputation **6**
2.1 Incidence of missing data . . . 6
2.2 Convergence of MCMC chains . . . 7
2.3 Observed and imputed values . . . 8
2.4 Observed and imputed values by year . . . 9
2.5 Pooled ANOVA results . . . 10

3 Midseason height (80 DAP) **13**
3.1 GGE biplot . . . 13
3.2 Observed and predicted values . . . 14
3.3 HPD intervals for inheritance classes . . . 15
3.4 HPD intervals for fixed and random effects . . . 15
3.5 Degree of dominance . . . 16
3.6 GxE . . . 17
3.6.1 GCA x environment interactions . . . 17
3.6.2 SCA x environment interactions . . . 18
3.6.3 Rankings by environment . . . 19

4 Height (130 DAP) **20**
4.1 GGE biplot . . . 20
4.2 Observed and predicted values . . . 21
4.3 HPD intervals for inheritance classes . . . 22
4.4 HPD intervals for fixed and random effects . . . 22
4.5 Degree of dominance . . . 23
4.6 GxE . . . 24
4.6.1 GCA x environment interactions . . . 24
4.6.2 Reciprocal x environment interactions . . . 25
4.6.3 Rankings by environment . . . 26

5 Midseason width (80 DAP) 27
5.1 GGE biplot . . . 27
5.2 Observed and predicted values . . . 28
5.3 HPD intervals for inheritance classes . . . 29
5.4 HPD intervals for fixed and random effects . . . 29
5.5 Degree of dominance . . . 30
5.6 GxE . . . 31
5.6.1 Rankings by environment . . . 31

6 Width (130 DAP) 32
6.1 GGE biplot . . . 32
6.2 Observed and predicted values . . . 33
6.3 HPD intervals for inheritance classes . . . 34
6.4 HPD intervals for fixed and random effects . . . 34
6.5 Degree of dominance . . . 35
6.6 GxE . . . 36
6.6.1 Rankings by environment . . . 36

7 Shoot biomass (fresh) 37
7.1 GGE biplot . . . 37
7.2 Observed and predicted values . . . 38
7.3 HPD intervals for inheritance classes . . . 39
7.4 HPD intervals for fixed and random effects . . . 39
7.5 Degree of dominance . . . 40
7.6 GxE . . . 41
7.6.1 GCA x environment interactions . . . 41
7.6.2 SCA x environment interactions . . . 42
7.6.3 Reciprocal x environment interactions . . . 43
7.6.4 Rankings by environment . . . 44

8 Shoot biomass (dry) 45
8.1 GGE biplot . . . 45
8.2 Observed and predicted values . . . 46
8.3 HPD intervals for inheritance classes . . . 47
8.4 HPD intervals for fixed and random effects . . . 47
8.5 Degree of dominance . . . 48
8.6 GxE . . . 49
8.6.1 GCA x environment interactions . . . 49
8.6.2 SCA x environment interactions . . . 50
8.6.3 Rankings by environment . . . 51

9 Root biomass (fresh) 52
9.1 GGE biplot 52
9.2 Observed and predicted values 53
9.3 HPD intervals for inheritance classes 54
9.4 HPD intervals for fixed and random effects 54
9.5 Degree of dominance 55
9.6 GxE 56
9.6.1 Rankings by environment 56

10 Root biomass (dry) 57
10.1 GGE biplot 57
10.2 Observed and predicted values 58
10.3 HPD intervals for inheritance classes 59
10.4 HPD intervals for fixed and random effects 59
10.5 Degree of dominance 60
10.6 GxE 61
10.6.1 Rankings by environment 61

11 Shoot:root ratio 62
11.1 GGE biplot 62
11.2 Observed and predicted values 63
11.3 HPD intervals for inheritance classes 64
11.4 HPD intervals for fixed and random effects 64
11.5 Degree of dominance 65
11.6 GxE 66
11.6.1 GCA x environment interactions 66
11.6.2 SCA x environment interactions 67
11.6.3 Rankings by environment 68

1. Parental lines

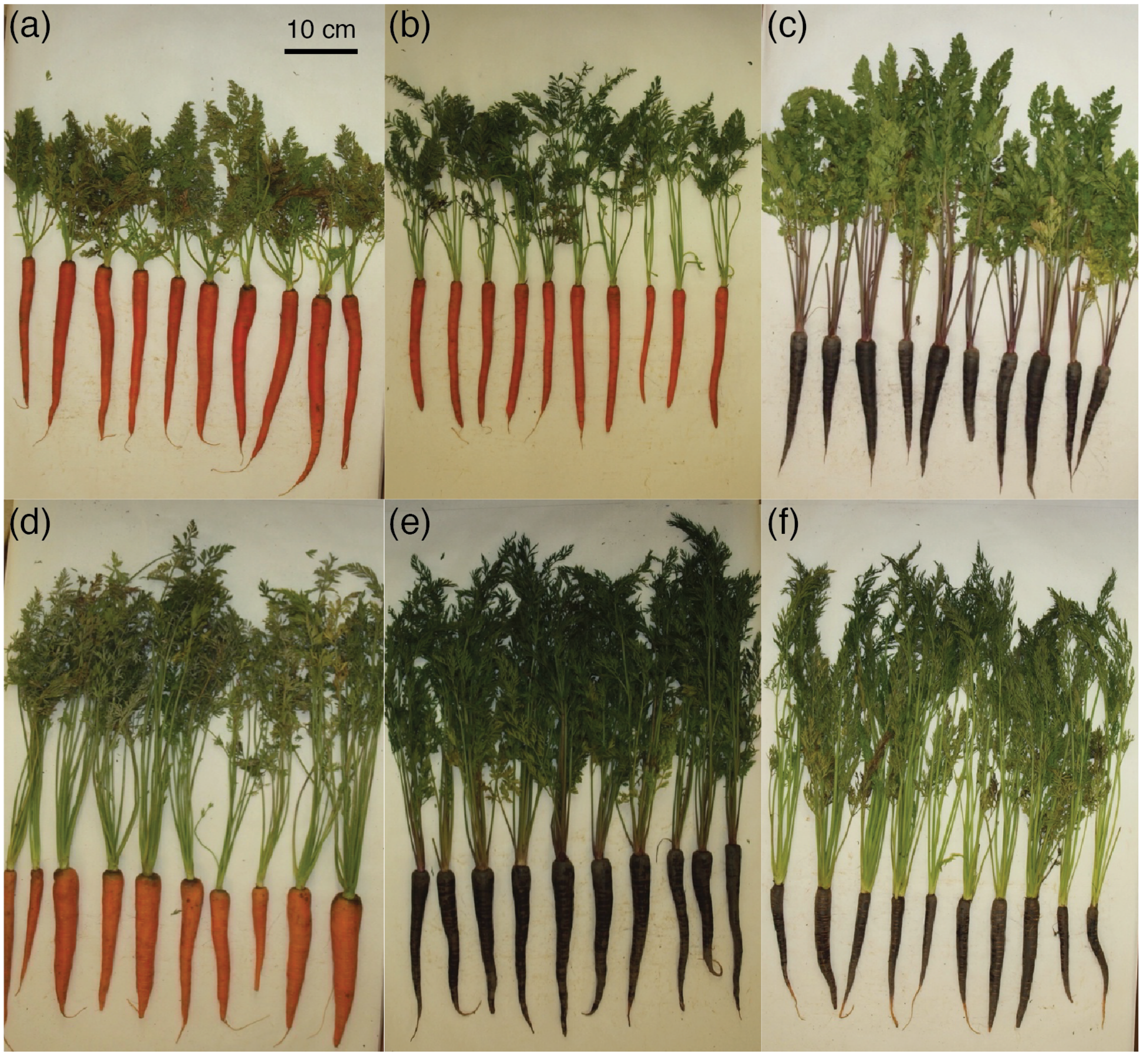

Figure S1: Carrot inbred lines used as parents in this study. (a) L6038, (b) L7550, (c) P0159, (d) Nbh2189, (e) P6139, (f) B7262.

1.1 Parental means

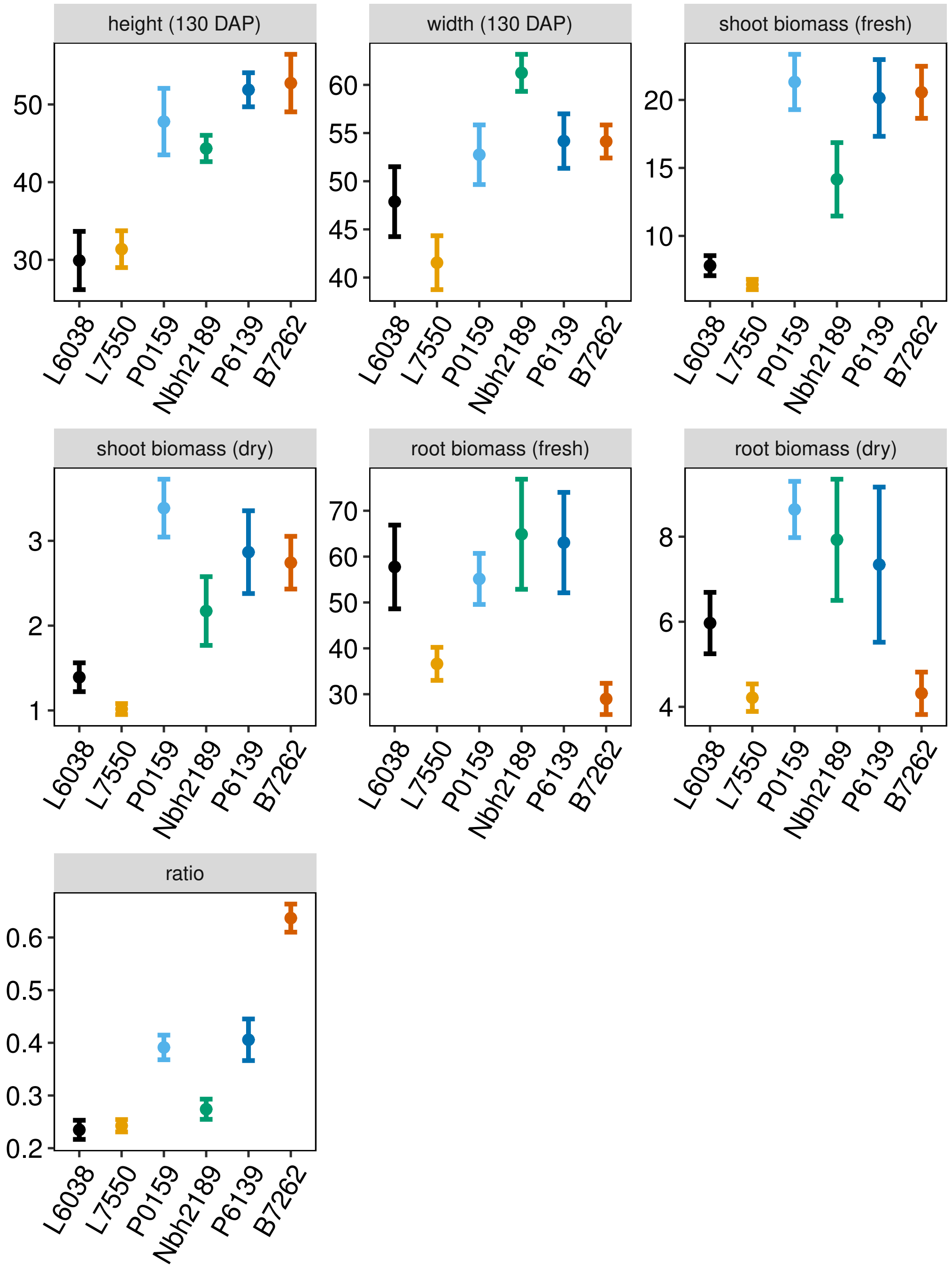

Figure S2: Variation among parental lines for selected phenotypes. Values are means ± 95% confidence intervals.

2. Multiple imputation

2.1 Incidence of missing data

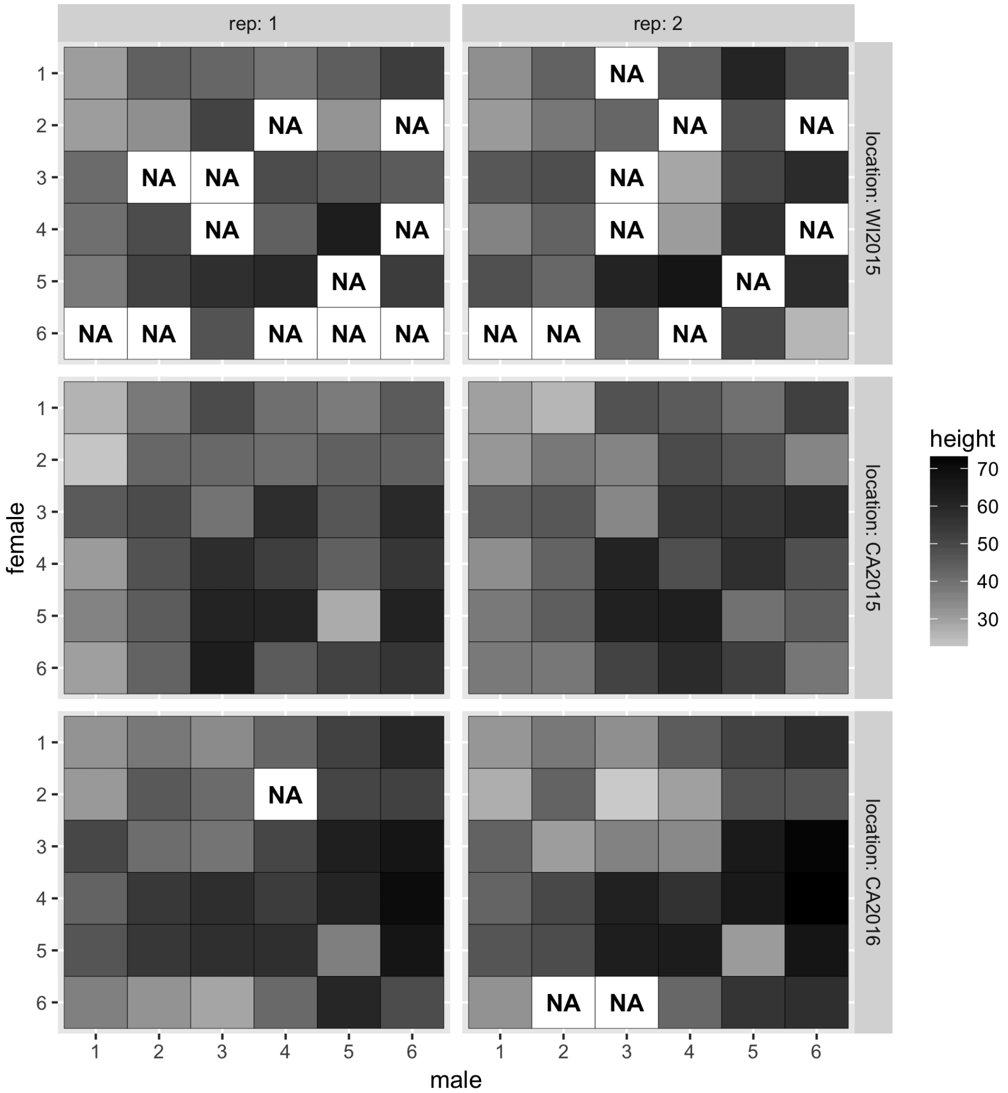

Figure S3: Incidence of missing data by replication and year for canopy height (130 DAP). Key: 1 = L6038, 2 = L7550, 3 = P0159, 4 = Nbh2189, 5 = P6139, 6 = B7262.

2.2 Convergence of MCMC chains

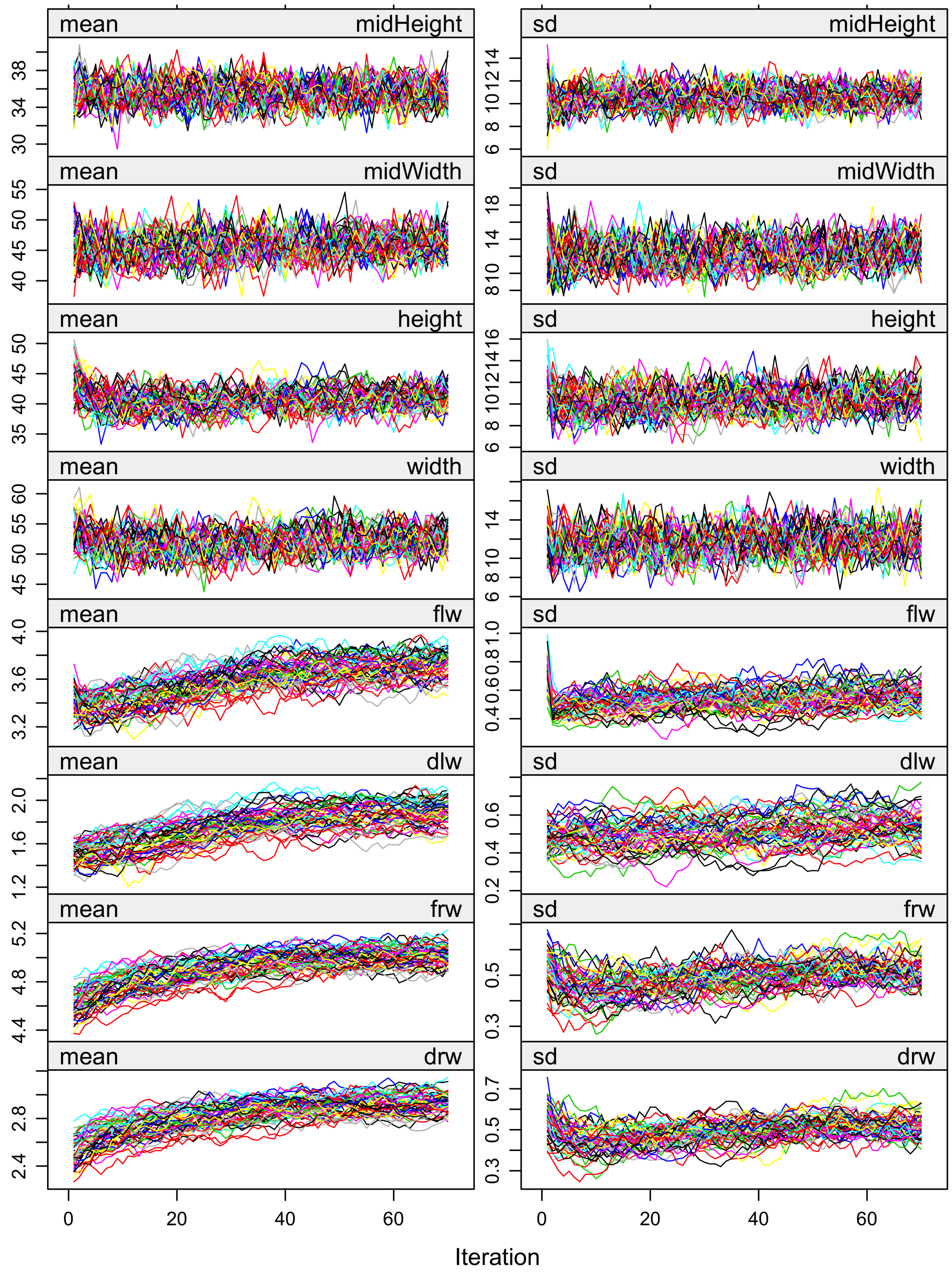

Figure S4: Convergence of imputed Markov chain Monte Carlo (MCMC) chains for all phenotypes. Key: flw = fresh shoot biomass, dlw = dry shoot biomass, frw = fresh root biomass, drw = dry root biomass.

2.3 Observed and imputed values

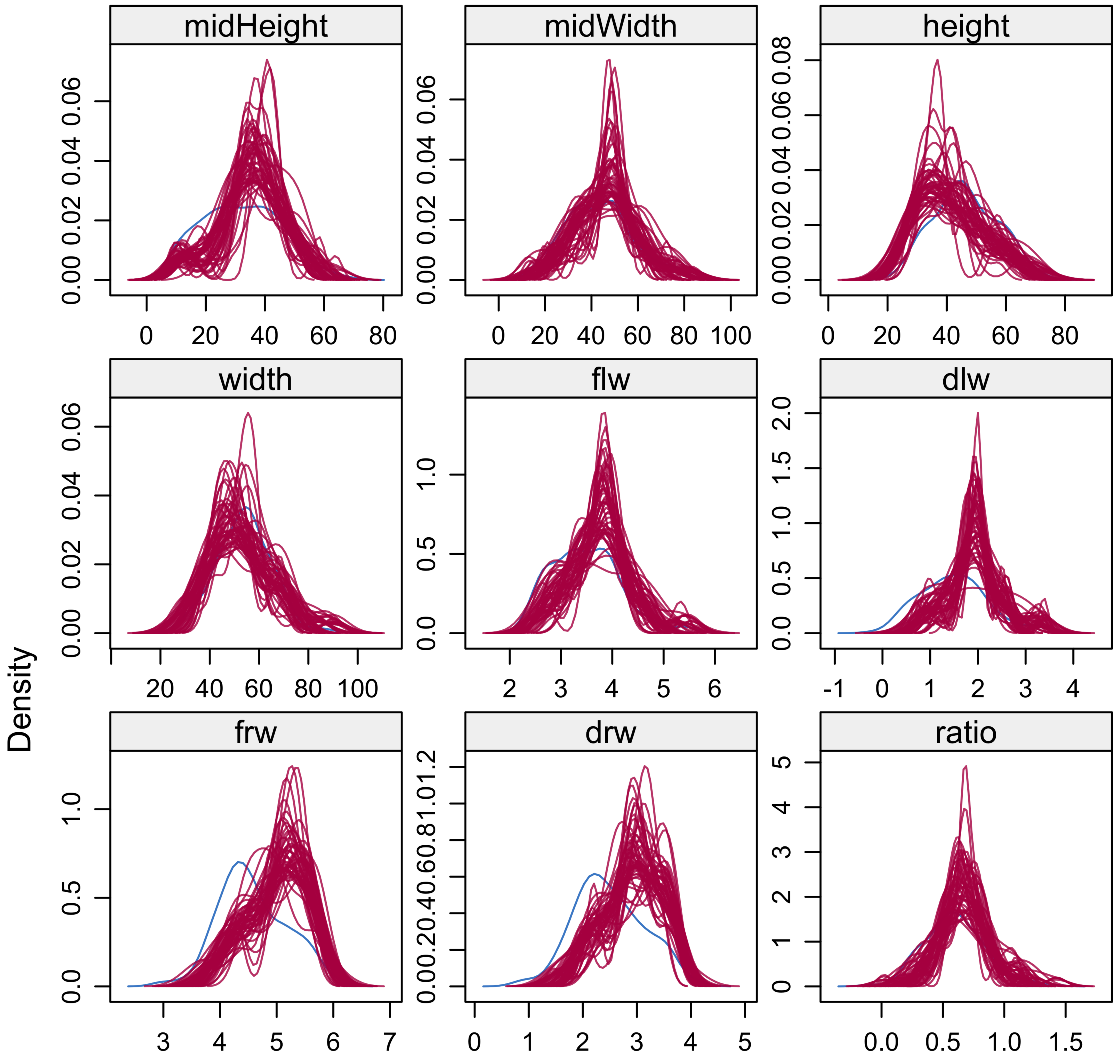

Figure S5: Distributions of observed (blue) and imputed (pink) values for all phenotypes. Key: flw = fresh shoot biomass, dlw = dry shoot biomass, frw = fresh root biomass, drw = dry root biomass.

2.4 Observed and imputed values by year

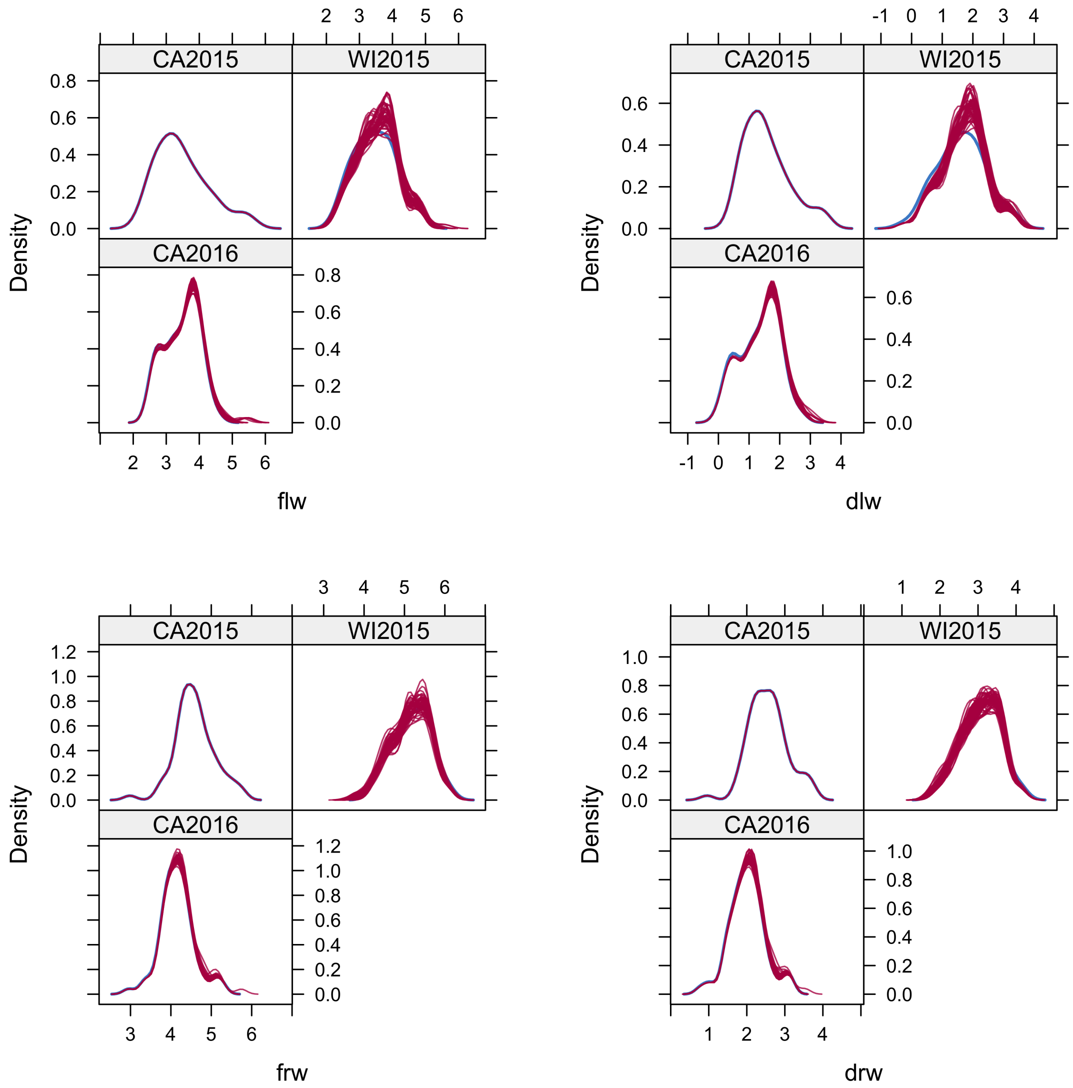

Figure S6: Distributions of observed (blue) and imputed (pink) values for selected traits by year. Key: flw = fresh shoot biomass, dlw = dry shoot biomass, frw = fresh root biomass, drw = dry root biomass.

2.5 Pooled ANOVA results

Table S1: Pooled results of Griffing's ANOVA (Method I, Model I) for multiply imputed data of carrot growth traits. Values include mean squares, critical F-values, corrected numerator degrees of freedom (rN), corrected denominator degrees of freedom (rD), and corresponding p-values.

Phenotype	Source	MS	F-value	rN	rD	P-value
	genotype	331.34	21.56	33.78	94.12	2.44E-31
	gca	516.06	33.42	4.88	94.12	3.12E-19
	sca	393.69	25.48	14.09	94.12	4.90E-26
	recip	207.42	13.44	14.29	94.12	5.36E-17
	year	10794.60	702.62	1.99	94.12	2.95E-57
height (80 DAP)	gxe	25.91	1.68	62.74	94.12	1.09E-02
	gcaxe	39.72	2.53	8.77	94.12	1.29E-02
	scaxe	25.26	1.63	26.49	94.12	4.50E-02
	recipxe	21.96	1.42	26.32	94.12	1.13E-01
	rep:year	24.00	1.32	1.43	94.12	2.65E-01
	residual	15.38	NA	NA	NA	NA
	genotype	489.30	15.29	34.20	81.71	2.00E-23
	gca	1488.95	46.49	4.97	81.71	1.96E-22
	sca	376.33	11.71	14.32	81.71	2.16E-14
	recip	269.07	8.34	13.83	81.71	1.01E-10
	year	268.70	7.96	1.78	81.71	1.10E-03
height (130 DAP)	gxe	68.48	2.14	62.81	81.71	6.62E-04
	gcaxe	134.49	4.12	8.89	81.71	2.23E-04
	scaxe	43.07	1.33	24.82	81.71	1.70E-01
	recipxe	71.88	2.23	26.85	81.71	3.02E-03
	rep:year	12.49	0.29	1.14	81.71	6.19E-01
	residual	32.14	NA	NA	NA	NA
	genotype	353.57	7.79	33.31	90.75	3.62E-15
	gca	363.35	7.92	4.74	90.75	4.76E-06
	sca	362.43	7.94	14.05	90.75	9.63E-11
	recip	341.46	7.49	14.15	90.75	3.28E-10
	year	11668.22	257.25	1.99	90.75	4.63E-38
width (80 DAP)	gxe	55.60	1.22	58.44	90.75	1.94E-01
	gcaxe	55.63	1.17	7.45	90.75	3.29E-01
	scaxe	36.82	0.79	20.52	90.75	7.24E-01
	recipxe	74.37	1.62	24.50	90.75	5.28E-02
	rep:year	56.44	0.99	1.21	90.75	3.39E-01
	residual	45.39	NA	NA	NA	NA

Continued on next page

Table S1 – continued from previous page

Phenotype	Source	MS	F-value	rN	rD	P-value
width (130 DAP)	genotype	433.77	8.16	32.89	90.48	1.13E-15
	gca	1024.37	19.22	4.91	90.48	7.41E-13
	sca	435.66	8.16	14.12	90.48	5.04E-11
	recip	235.02	4.37	13.19	90.48	1.02E-05
	year	944.08	17.26	1.88	90.48	7.78E-07
	gxe	64.74	1.21	58.34	90.48	2.01E-01
	gcaxe	110.75	2.03	8.44	90.48	4.85E-02
	scaxe	50.95	0.95	25.05	90.48	5.41E-01
	recipxe	63.20	1.18	25.31	90.48	2.80E-01
	rep:year	106.03	1.98	1.96	90.48	1.45E-01
	residual	53.17	NA	NA	NA	NA
shoot biomass (fresh)	genotype	1.80	15.67	33.26	83.96	4.99E-24
	gca	7.27	63.32	4.96	83.96	5.99E-27
	sca	0.95	8.18	13.81	83.96	1.22E-10
	recip	0.83	7.13	13.11	83.96	3.61E-09
	year	0.11	0.05	0.06	83.96	1.61E-01
	gxe	0.26	2.27	58.11	83.96	3.00E-04
	gcaxe	0.45	3.88	9.05	83.96	3.69E-04
	scaxe	0.26	2.29	28.00	83.96	1.94E-03
	recipxe	0.19	1.67	23.85	83.96	4.50E-02
	rep:year	0.10	0.77	1.77	83.96	4.51E-01
	residual	0.12	NA	NA	NA	NA
shoot biomass (dry)	genotype	2.13	15.35	33.41	93.50	1.93E-25
	gca	8.55	61.63	4.96	93.50	5.33E-28
	sca	1.21	8.70	13.87	93.50	1.07E-11
	recip	0.91	6.45	13.33	93.50	1.11E-08
	year	2.06	14.22	1.82	93.50	8.18E-06
	gxe	0.28	1.98	58.09	93.50	1.61E-03
	gcaxe	0.40	2.80	8.87	93.50	6.14E-03
	scaxe	0.31	2.25	27.50	93.50	2.06E-03
	recipxe	0.20	1.40	23.10	93.50	1.33E-01
	rep:year	0.12	0.69	1.44	93.50	4.57E-01
	residual	0.14	NA	NA	NA	NA

Continued on next page

Table S1 – continued from previous page

Phenotype	Source	MS	F-value	rN	rD	P-value
	genotype	0.76	8.73	33.57	94.71	4.15E-17
	gca	1.13	12.99	4.81	94.71	2.02E-09
	sca	1.11	12.76	14.51	94.71	1.74E-16
	recip	0.28	3.18	13.03	94.71	5.45E-04
	year	17.80	205.50	1.99	94.71	4.18E-35
root biomass (fresh)	gxe	0.12	1.44	62.13	94.71	5.37E-02
	gcaxe	0.21	2.40	9.18	94.71	1.64E-02
	scaxe	0.14	1.55	25.69	94.71	6.63E-02
	recipxe	0.09	0.98	24.86	94.71	4.95E-01
	rep:year	0.14	1.53	1.88	94.71	2.22E-01
	residual	0.09	NA	NA	NA	NA
	genotype	0.91	9.26	33.49	95.15	5.75E-18
	gca	1.74	17.55	4.88	95.15	3.99E-12
	sca	1.30	13.22	14.44	95.15	6.11E-17
	recip	0.24	2.43	12.18	95.15	8.24E-03
	year	19.71	200.19	1.99	95.15	9.40E-35
root biomass (dry)	gxe	0.12	1.26	59.40	95.15	1.54E-01
	gcaxe	0.20	1.97	9.01	95.15	5.11E-02
	scaxe	0.13	1.33	25.13	95.15	1.66E-01
	recipxe	0.09	0.94	24.92	95.15	5.53E-01
	rep:year	0.15	1.41	1.80	95.15	2.48E-01
	residual	0.10	NA	NA	NA	NA
	genotype	0.24	17.39	32.86	82.36	3.51E-25
	gca	1.27	93.46	4.95	82.36	2.47E-32
	sca	0.06	4.19	12.68	82.36	2.94E-05
	recip	0.07	5.09	12.96	82.36	1.61E-06
	year	0.27	18.69	1.79	82.36	5.84E-07
shoot:root ratio	gxe	0.04	2.69	60.59	82.36	1.64E-05
	gcaxe	0.08	6.15	8.99	82.36	1.42E-06
	scaxe	0.04	3.03	27.97	82.36	5.34E-05
	recipxe	0.02	1.15	21.92	82.36	3.13E-01
	rep:year	0.01	0.33	0.67	82.36	4.82E-01
	residual	0.01	NA	NA	NA	NA

3. Midseason height (80 DAP)

3.1 GGE biplot

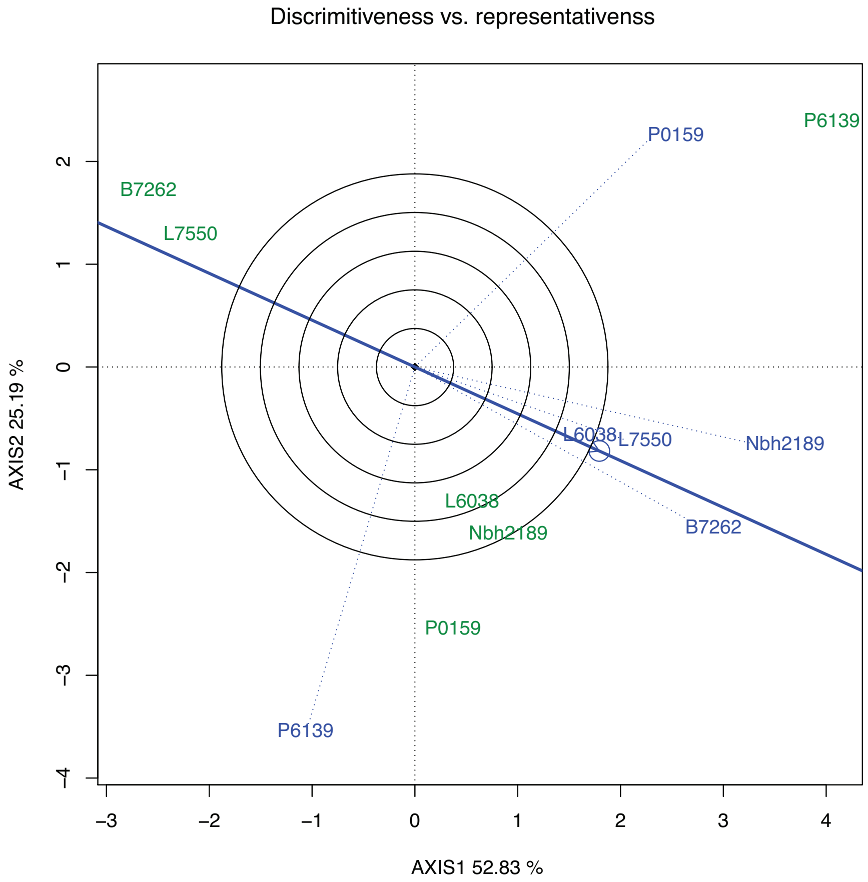

Figure S7: Discriminativeness vs. representativeness view of a biplot for canopy height at 80 days after planting. Parents treated as testers are shown in blue and as entries in green. The blue line indicates the average tester coordinate (ATC) abscissa, with the blue circle indicating the average tester score. The arrow in the circle indicates the direction of increasing GCA for the entries. Parent B7262 was both the most discriminating (had the longest vector) and was representative of other parental lines (vector was close to the ATC abscissa).

3.2 Observed and predicted values

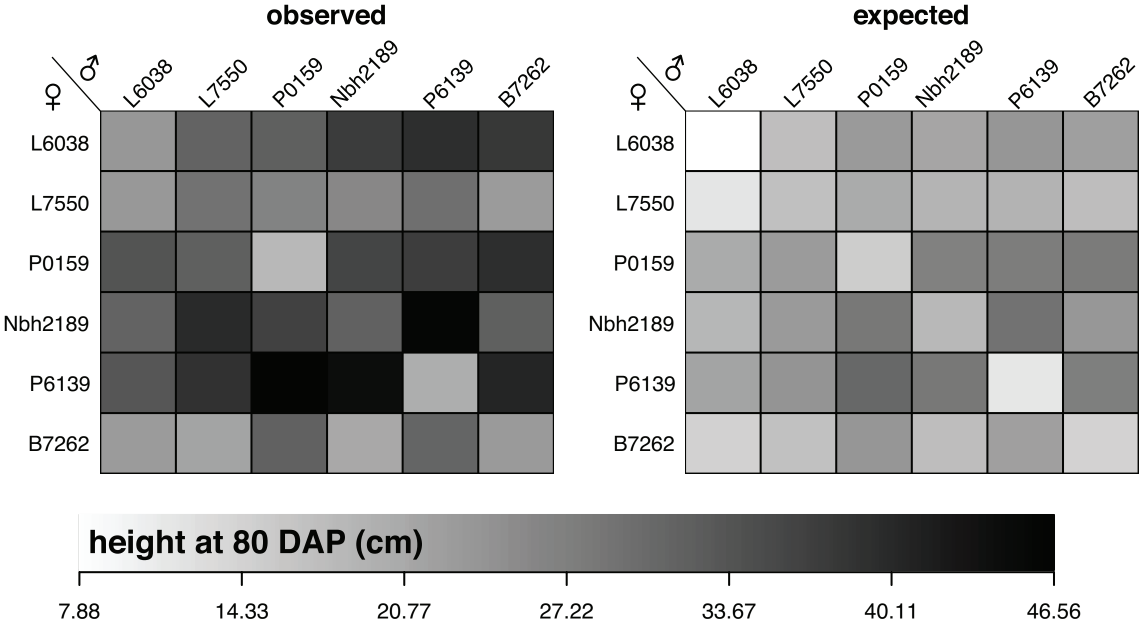

Figure S8: Observed (left) and predicted (right) means for height (80 DAP). Posterior predicted means on the right are the result of fitting the model in BayesDiallel to the data on the left.

3.3 HPD intervals for inheritance classes

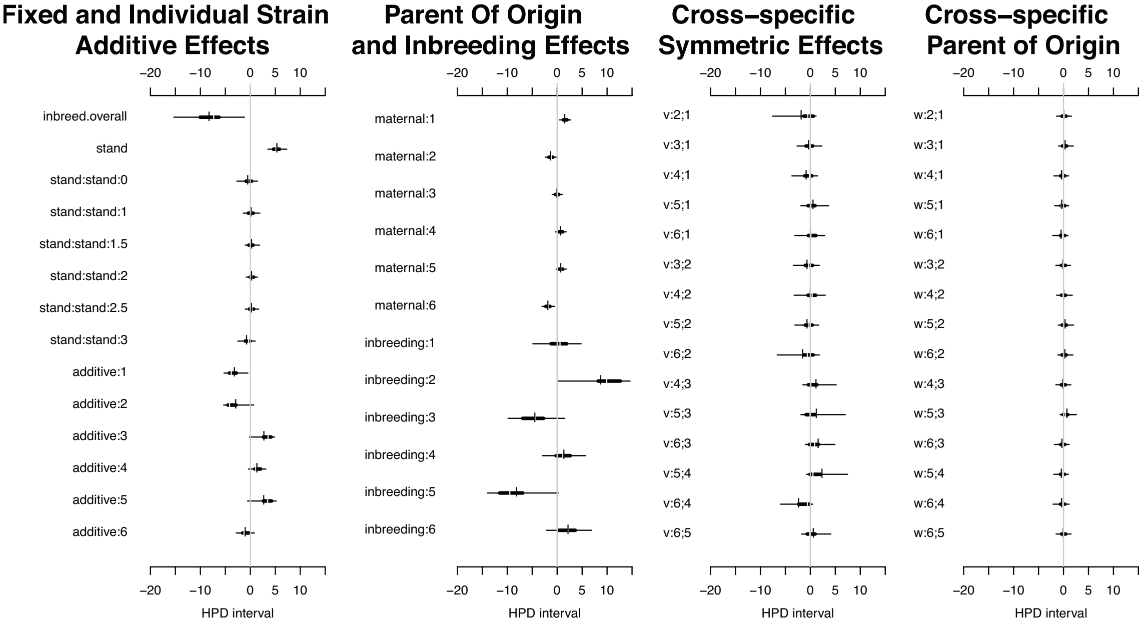

Figure S9: HPD intervals for canopy height (80 DAP; cm).

3.4 HPD intervals for fixed and random effects

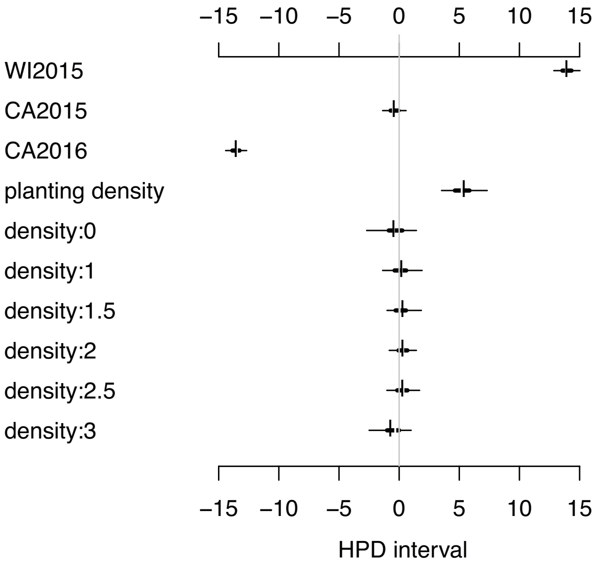

Figure S10: HPD intervals of fixed and random effects for canopy height (80 DAP; cm). Planting density ranged from 0 (low) to 3 (high).

3.5 Degree of dominance

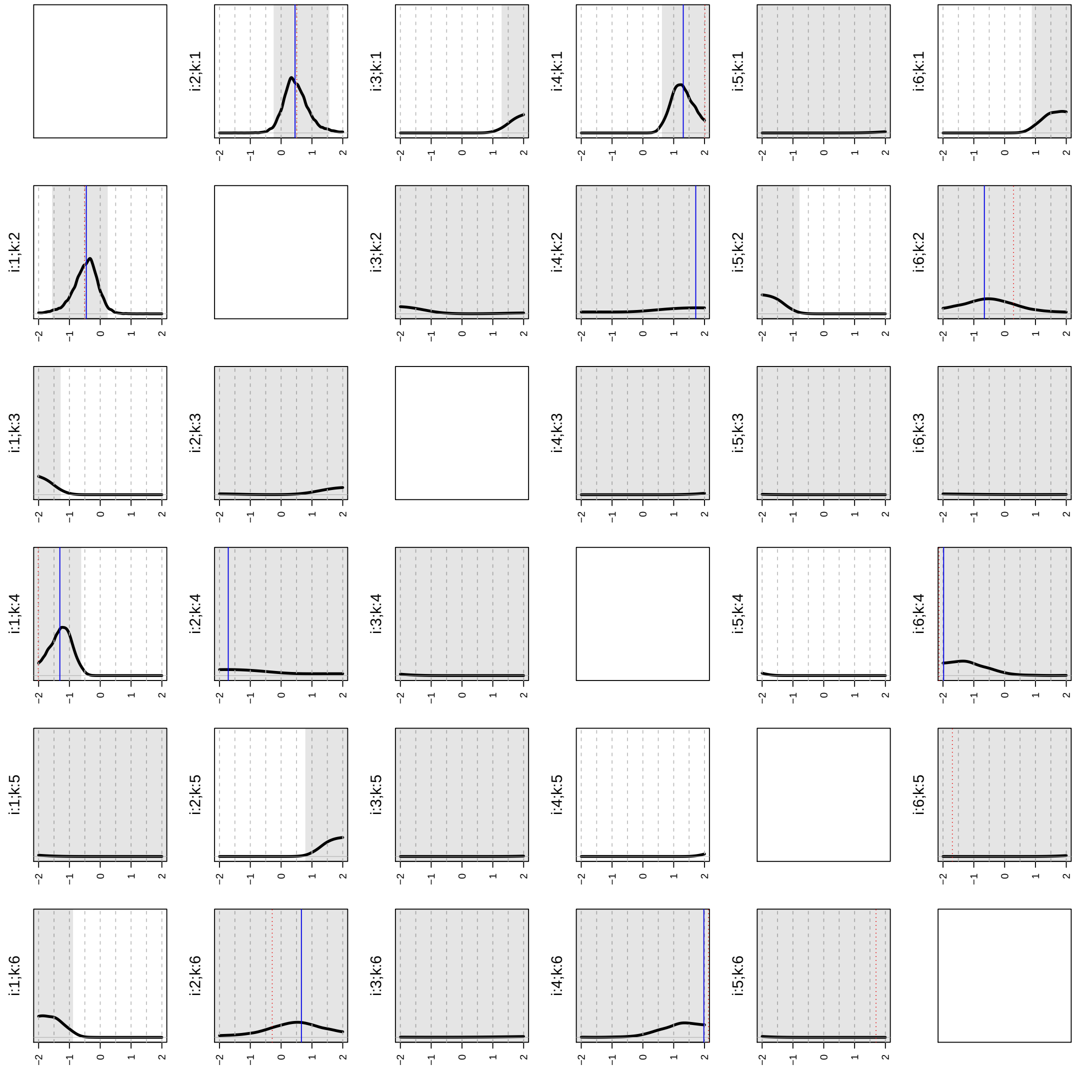

Figure S11: Posterior distributions of the degree of dominance for midseason height (80 DAP). Posterior means within the interval are indicated by red dotted lines and medians by blue solid lines, with 95% central quantiles shown in grey. A signal was considered strong if the mean, median, and the majority of the posterior distribution fell within the specified ranges: pseudo-underrecessive (-∞,-1.5), recessive (-1.5,-0.5), additive (-0.5,0.5), dominant (0.5,1.5), pseudo-overdominant (1.5,∞). Key for parental lines: 1=L6038, 2=L7550, 3=P0159, 4=Nbh2189, 5=P6139, 6=B7262.

3.6 GxE

3.6.1 GCA x environment interactions

Table S2: GCA for plant height (80 DAP; cm) in each growing environment.

Parent	WI2015	CA2015	CA2016
L6038	-3.45	-0.65	-0.06
L7550	-2.78	-2.9	-2.81*
P0159	1.16	2.17	-0.77
Nbh2189	2.88	1.24	3.17*
P6139	4.19*	3.52*	2.84*
B7262	-1.99	-3.38*	-2.37

***P≤0.001, **P≤0.01, *P≤0.05

Table S3: Nonparametric correlations (Spearman's rho) among environments for GCA of plant height (80 DAP).

Environment	Spearman's rho
WI2015-CA2015	0.71
WI2015-CA2016	0.60
CA2015-CA2016	0.66

***P≤0.001, **P≤0.01, *P≤0.05

3.6.2 SCA x environment interactions

Table S4: SCA for plant height (80 DAP; cm) in each growing environment.

F1 hybrids	WI2015	CA2015	CA2016
L6038 x L7550	2.16	-2.48	-1.26
L6038 x P0159	0.69	2.83	-0.37
L6038 x Nbh2189	-1.48	2.3	2.22
L6038 x P6139	-0.01	3.56	1.44
L6038 x B7262	1.21	0.91	4.02
L7550 x P0159	-3.06	2.04	-2.22
L7550 x Nbh2189	-1.27	1.89	0.83
L7550 x P6139	-1.84	3.4	-0.54
L7550 x B7262	-3.77	-6*	-0.54
P0159 x Nbh2189	1.95	2.03	0.38
P0159 x P6139	0.1	5.12	6.9*
P0159 x B7262	2.39	6.89*	3.9
Nbh2189 x P6139	7*	4.22	5.48
Nbh2189 x B7262	-6.11	-5.97*	-4.28
P6139 x B7262	-0.95	5.66	2.24
**Parental selfs**			
L6038	-3.5	-7.11	-5.11
L7550	3.31	1.15	2.6
P0159	-13.34**	-18.9***	-7.12
Nbh2189	-9.31*	-4.46	-5.45
P6139	-20.01***	-21.94***	-14.58***
B7262	-6.33	-1.49	-2.44

***P≤0.001, **P≤0.01, *P≤0.05

Table S5: Nonparametric correlations (Spearman's rho) among environments for SCA of plant height (80 DAP).

Environment	Spearman's rho
WI2015-CA2015	0.64**
WI2015-CA2016	0.82***
CA2015-CA2016	0.79***

***P≤0.001, **P≤0.01, *P≤0.05

3.6.3 Rankings by environment

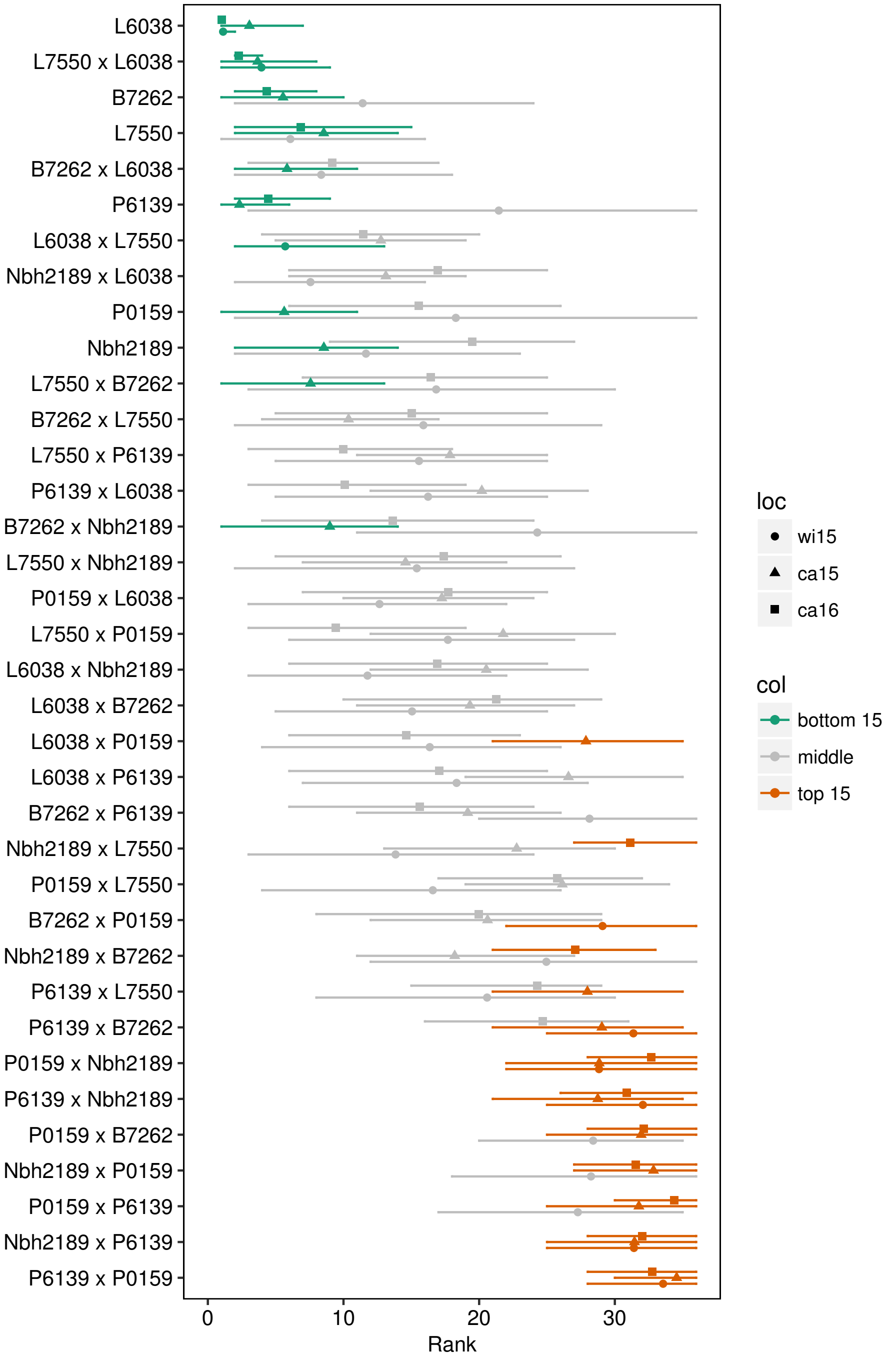

Figure S12: Mean values and 95% highest posterior density (HPD) intervals of hybrid rankings for canopy height (cm; 80 DAP). Hybrids with intervals in the bottom 15 are shown in blue and hybrids with intervals in the top 15 are shown in red.

4. Height (130 DAP)

4.1 GGE biplot

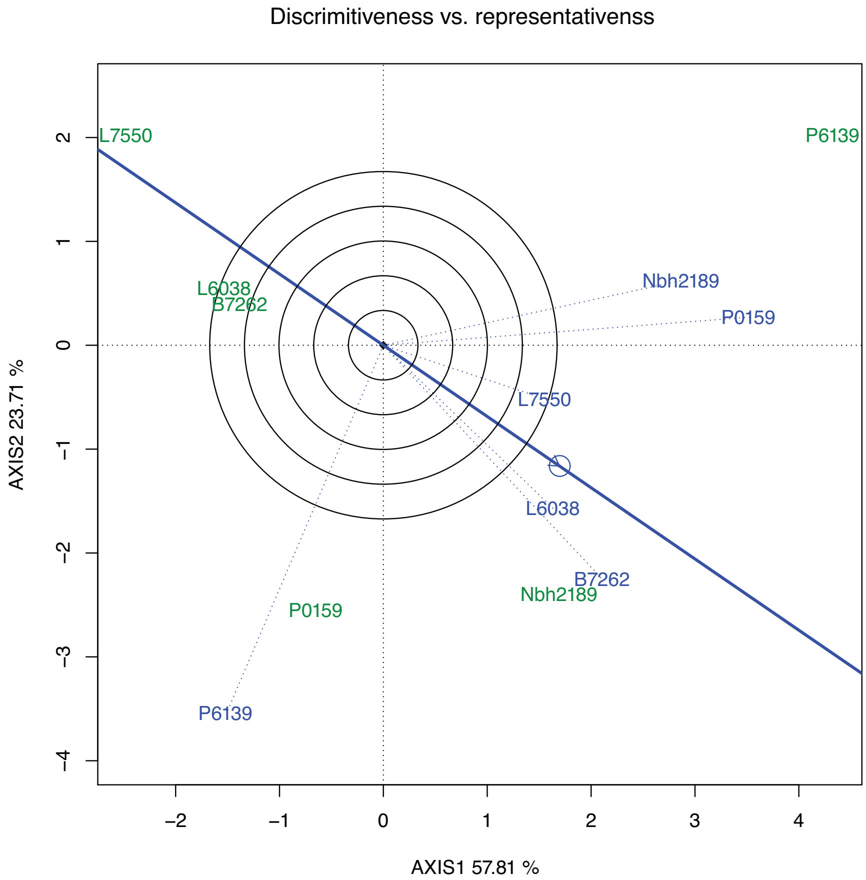

Figure S13: Discriminativeness vs. representativeness view of a biplot for canopy height at 130 days after planting. Parents treated as testers are shown in blue and as entries in green. The blue line indicates the average tester coordinate (ATC) abscissa, with the blue circle indicating the average tester score. The arrow in the circle indicates the direction of increasing GCA for the entries. Parent B7262 was both the most discriminating (had the longest vector) and was representative of other parental lines (vector was close to the ATC abscissa).

4.2 Observed and predicted values

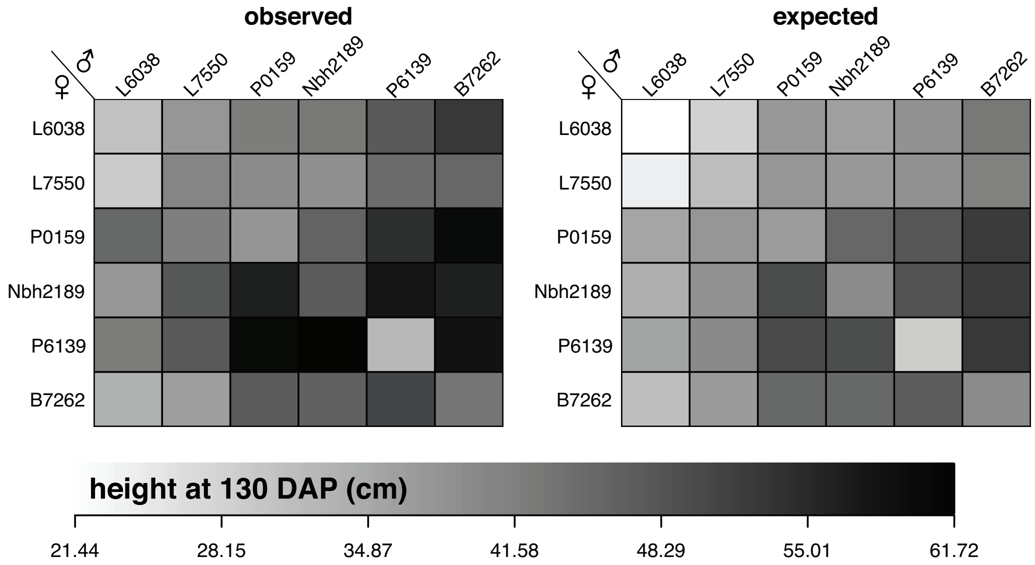

Figure S14: Observed (left) and predicted (right) means for height (130 DAP). Posterior predicted means on the right are the result of fitting the model in BayesDiallel to the data on the left.

4.3 HPD intervals for inheritance classes

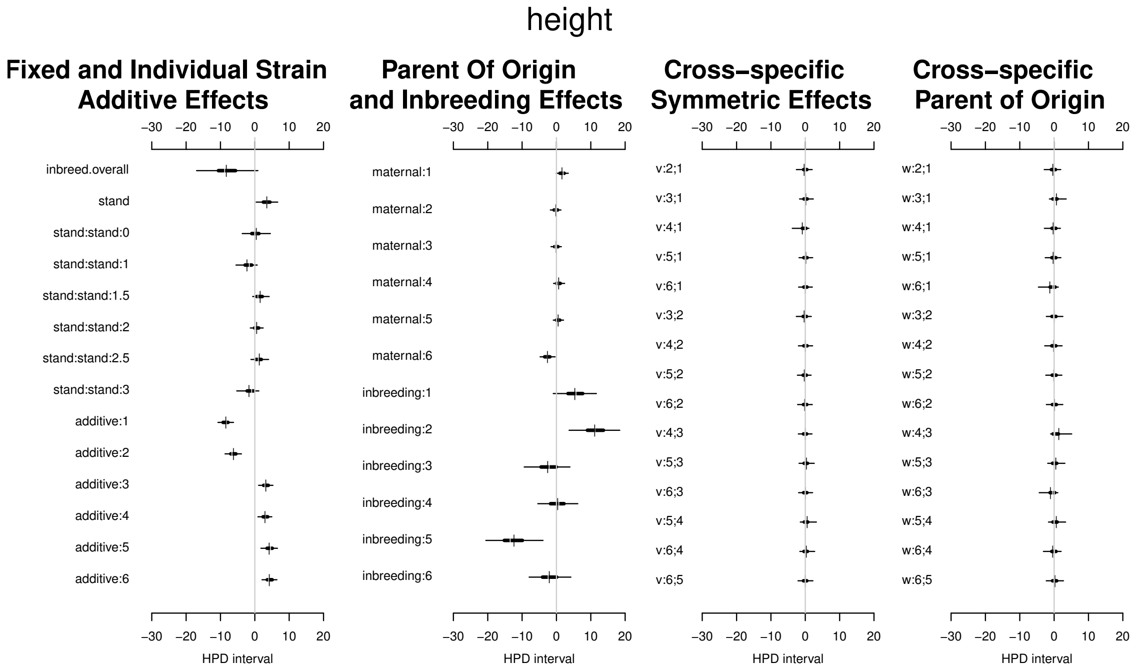

Figure S15: HPD intervals for canopy height (130 DAP; cm).

4.4 HPD intervals for fixed and random effects

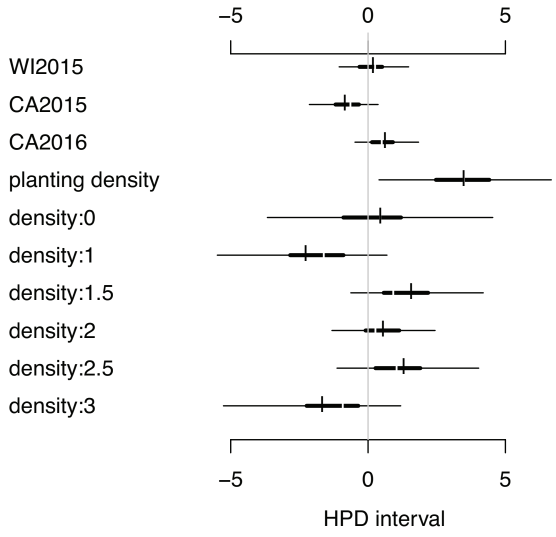

Figure S16: HPD intervals of fixed and random effects for canopy height (130 DAP; cm). Planting density ranged from 0 (low) to 3 (high).

4.5 Degree of dominance

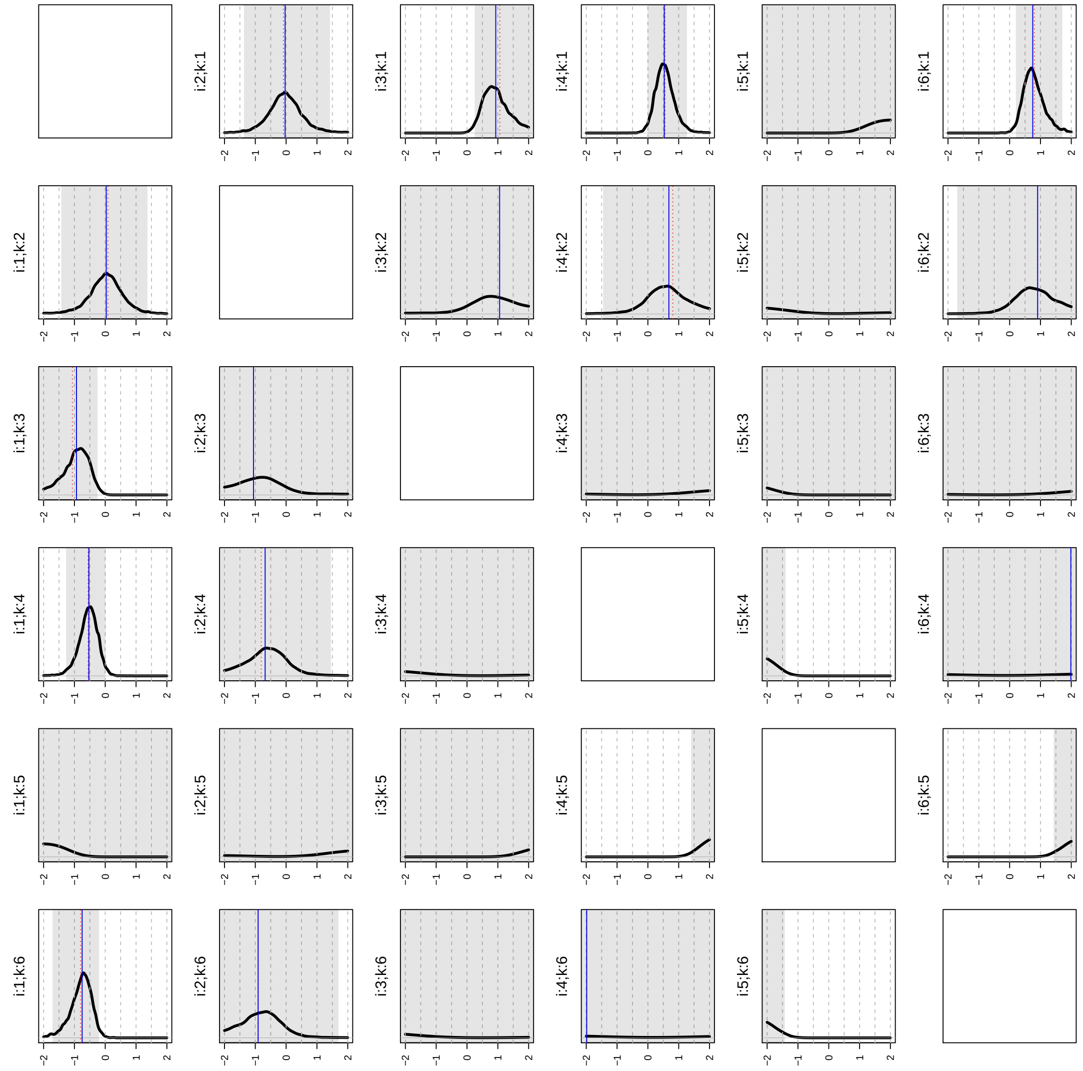

Figure S17: Posterior distributions of the degree of dominance for height (130 DAP). Posterior means within the interval are indicated by red dotted lines and medians by blue solid lines, with 95% central quantiles shown in grey. A signal was considered strong if the mean, median, and the majority of the posterior distribution fell within the specified ranges: pseudo-underrecessive (-∞,-1.5), recessive (-1.5,-0.5), additive (-0.5,0.5), dominant (0.5,1.5), pseudo-overdominant (1.5,∞). Key for parental lines: 1=L6038, 2=L7550, 3=P0159, 4=Nbh2189, 5=P6139, 6=B7262.

4.6 GxE

4.6.1 GCA x environment interactions

Table S6: GCA for plant height (130 DAP; cm) in each growing environment.

Parent	WI2015	CA2015	CA2016
L6038	-4.14*	-8.52***	-6.83***
L7550	-3.78	-4.83**	-6.21**
P0159	1.21	4.44**	-1.37
Nbh2189	2.31	4.35*	4.2
P6139	4.65*	1.51	5.44*
B7262	-0.25	3.05	4.77*

***P≤0.001, **P≤0.01, *P≤0.05

Table S7: Nonparametric correlations (Spearman's rho) among environments for GCA of plant height (130 DAP).

Environment	Spearman's rho
WI2015-CA2015	0.60
WI2015-CA2016	0.83*
CA2015-CA2016	0.43

***P≤0.001, **P≤0.01, *P≤0.05

4.6.2 Reciprocal x environment interactions

Table S8: Reciprocal effects for plant height (130 DAP; cm) in each location.

F1 hybrids	WI2015	CA2015	CA2016
L6038 x L7550	6.38**	2.25	4.42*
L6038 x P0159	-1.93	1.75	-6.46**
L6038 x Nbh2189	1.83	5.13*	0.33
L6038 x P6139	4.83*	0.92	2.54
L6038 x B7262	10.71***	7.29***	11.79***
L7550 x L6038	-6.38**	-2.25	-4.42*
L7550 x P0159	1.45	-4.38*	-1.92
L7550 x Nbh2189	-4.75	-0.08	-8.06**
L7550 x P6139	-3.42	0.29	-1.38
L7550 x B7262	1.58	-0.67	6.46*
P0159 x L6038	1.93	-1.75	6.46**
P0159 x L7550	-1.45	4.38*	1.92
P0159 x Nbh2189	-9.58*	-1.75	-8.56***
P0159 x P6139	-5.25**	-5.5**	1.88
P0159 x B7262	3.85	0.71	15.65***
Nbh2189 x L6038	-1.83	-5.13*	-0.33
Nbh2189 x L7550	4.75	0.08	8.06**
Nbh2189 x P0159	9.58*	1.75	8.56***
Nbh2189 x P6139	-1.33	-5.71**	1.29
Nbh2189 x B7262	7.46	-0.13	14.71***
P6139 x L6038	-4.83*	-0.92	-2.54
P6139 x L7550	3.42	-0.29	1.38
P6139 x P0159	5.25**	5.5**	-1.88
P6139 x Nbh2189	1.33	5.71**	-1.29
P6139 x B7262	2.65	0.54	4.54*
B7262 x L6038	-10.71***	-7.29***	-11.79***
B7262 x L7550	-1.58	0.67	-6.46*
B7262 x P0159	-3.85	-0.71	-15.65***
B7262 x Nbh2189	-7.46	0.13	-14.71***
B7262 x P6139	-2.65	-0.54	-4.54*

***P≤0.001, **P≤0.01, *P≤0.05

Table S9: Nonparametric correlations (Spearman's rho) among environments for reciprocal effects of plant height (130 DAP).

Environment	Spearman's rho
WI2015-CA2015	0.53***
WI2015-CA2016	0.79***
CA2015-CA2016	0.28

***P≤0.001, **P≤0.01, *P≤0.05

4.6.3 Rankings by environment

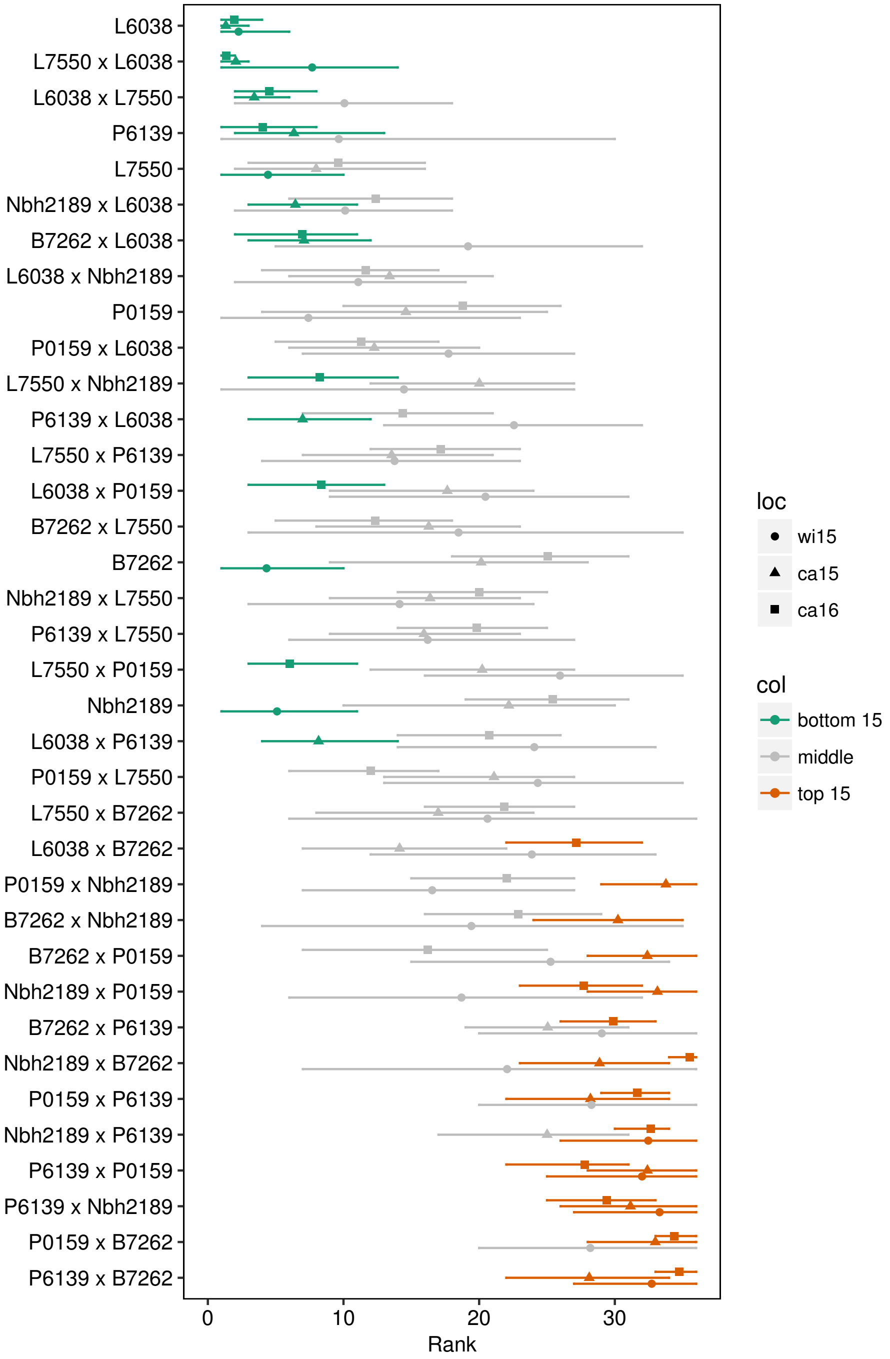

Figure S18: Mean values and 95% highest posterior density (HPD) intervals of hybrid rankings for canopy height (cm; 130 DAP). Hybrids with intervals in the bottom 15 are shown in blue and hybrids with intervals in the top 15 are shown in red.

5. Midseason width (80 DAP)

5.1 GGE biplot

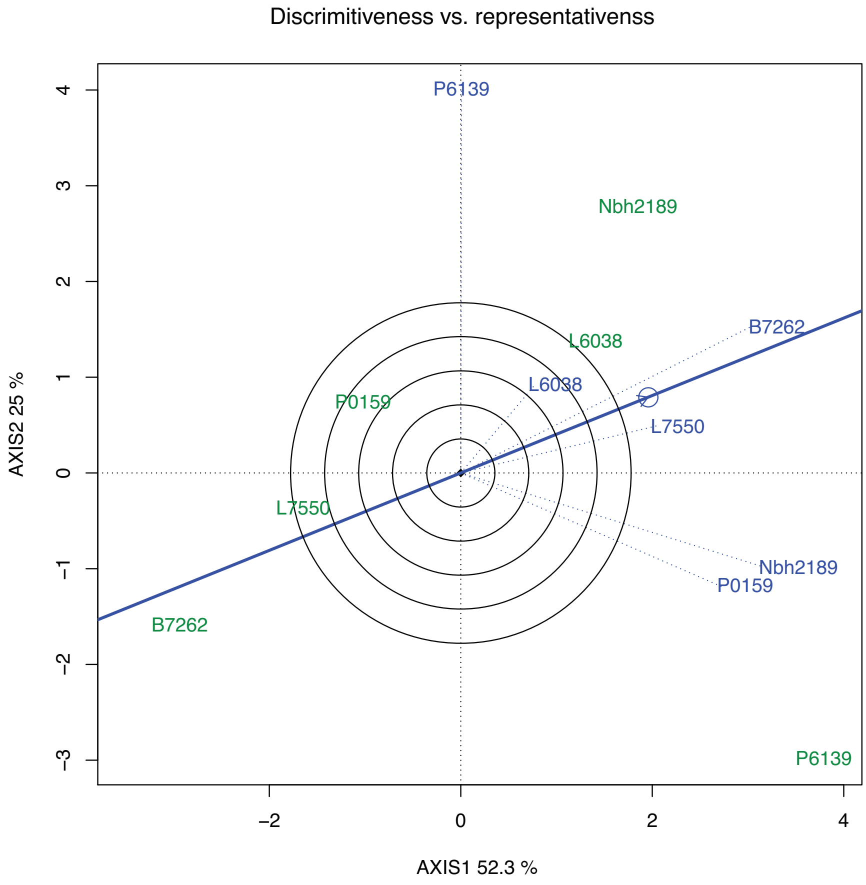

Figure S19: Discriminativeness vs. representativeness view of a biplot for canopy width at 80 days after planting. Parents treated as testers are shown in blue and as entries in green. The blue line indicates the average tester coordinate (ATC) abscissa, with the blue circle indicating the average tester score. The arrow in the circle indicates the direction of increasing GCA for the entries. Parent B7262 was both the most discriminating (had the longest vector) and was representative of other parental lines (vector was close to the ATC abscissa).

5.2 Observed and predicted values

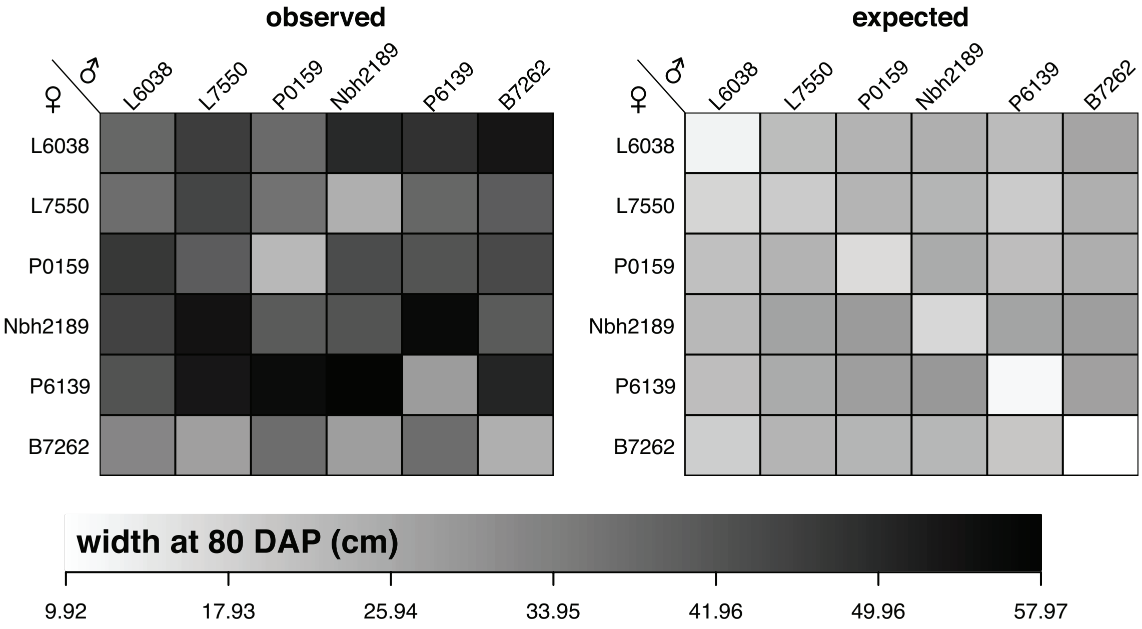

Figure S20: Observed (left) and predicted (right) means for width (80 DAP). Posterior predicted means on the right are the result of fitting the model in BayesDiallel to the data on the left.

5.3 HPD intervals for inheritance classes

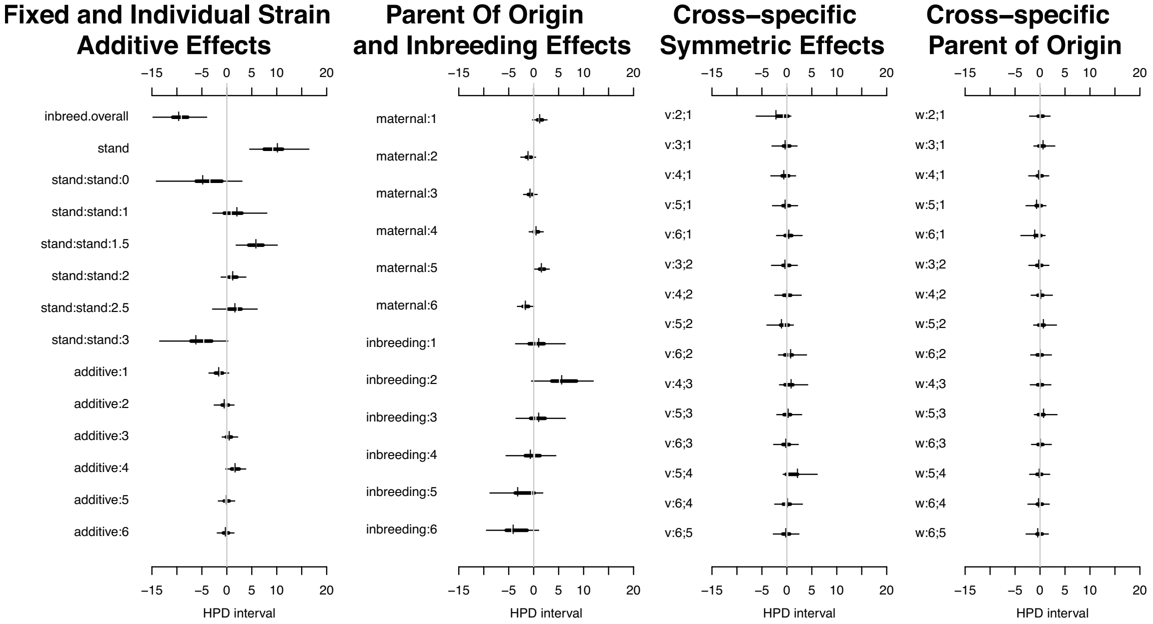

Figure S21: HPD intervals for canopy width (80 DAP; cm).

5.4 HPD intervals for fixed and random effects

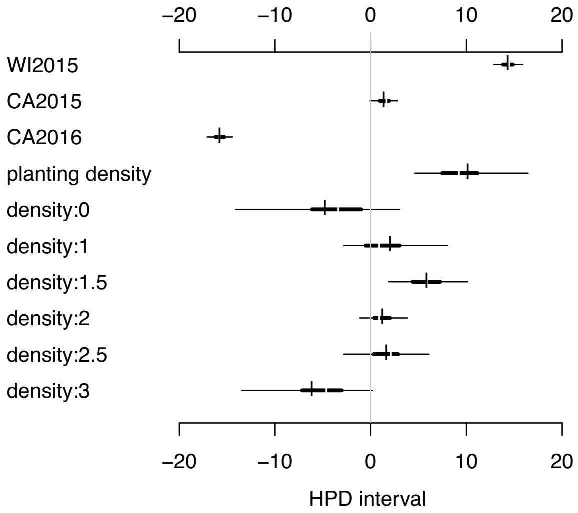

Figure S22: HPD intervals of fixed and random effects for canopy width (80 DAP; cm). Planting density ranged from 0 (low) to 3 (high).

5.5 Degree of dominance

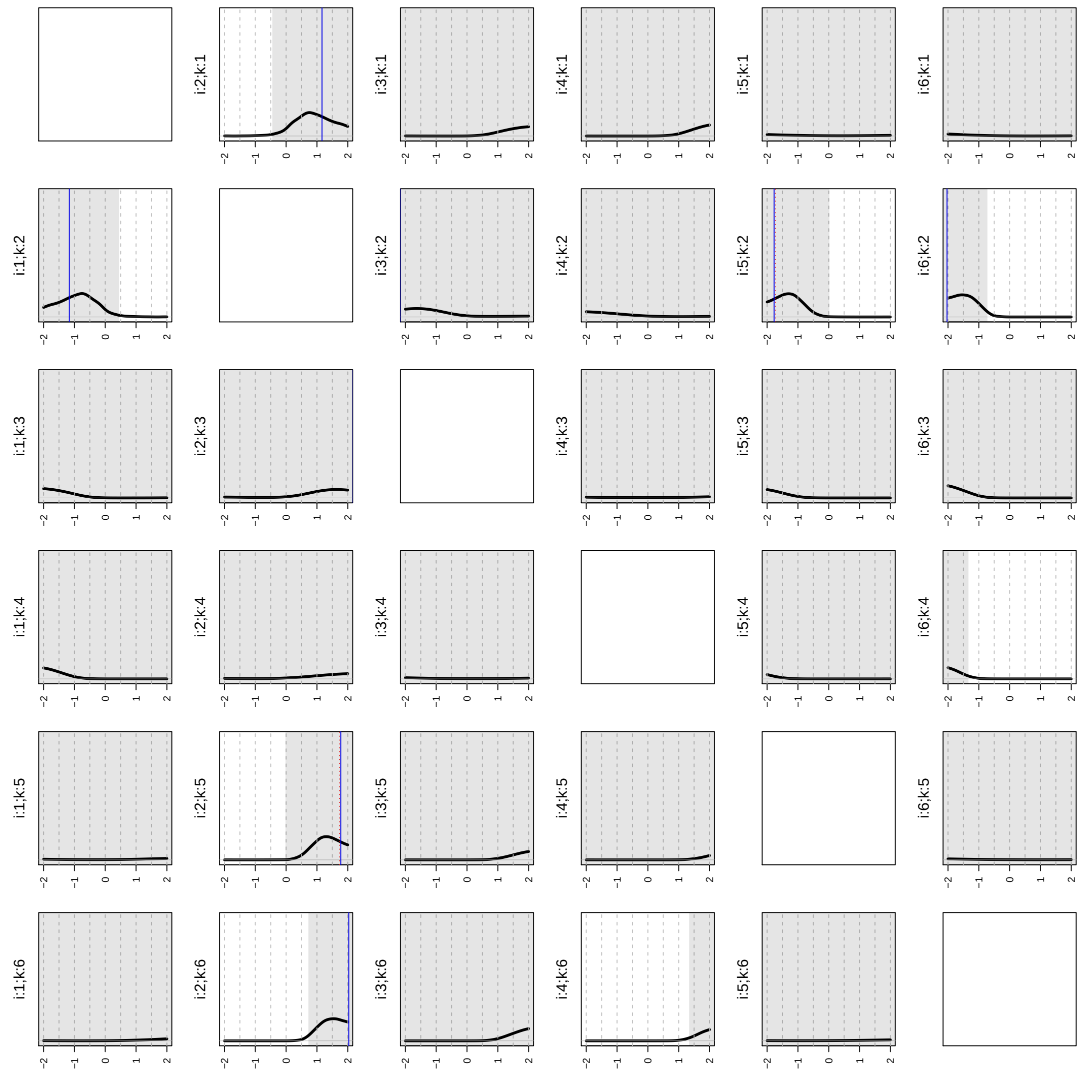

Figure S23: Posterior distributions of the degree of dominance for midseason width (80 DAP). Posterior means within the interval are indicated by red dotted lines and medians by blue solid lines, with 95% central quantiles shown in grey. A signal was considered strong if the mean, median, and the majority of the posterior distribution fell within the specified ranges: pseudo-underrecessive (-∞,-1.5), recessive (-1.5,-0.5), additive (-0.5,0.5), dominant (0.5,1.5), pseudo-overdominant (1.5,∞). Key for parental lines: 1=L6038, 2=L7550, 3=P0159, 4=Nbh2189, 5=P6139, 6=B7262.

5.6 GxE

5.6.1 Rankings by environment

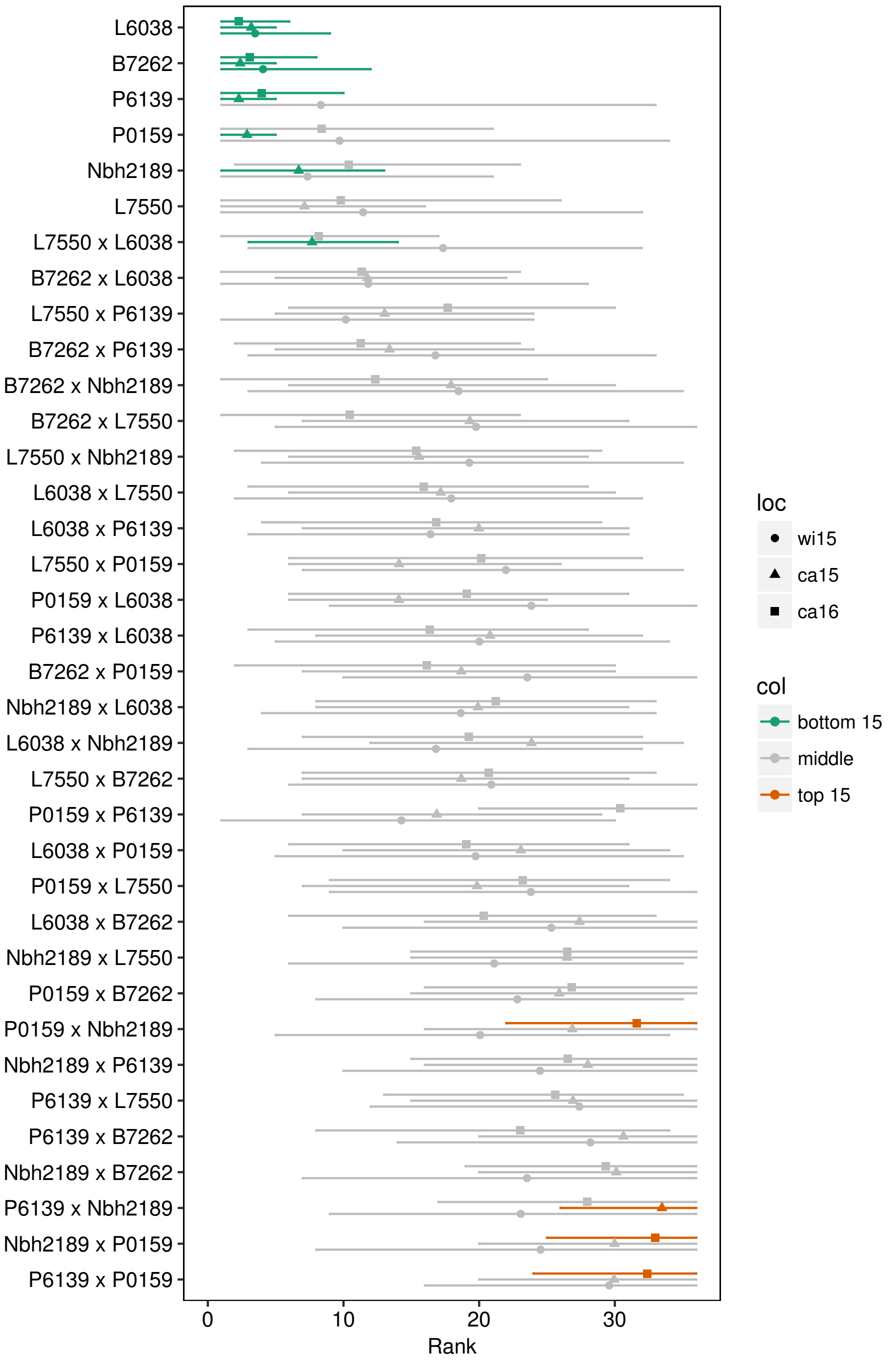

Figure S24: Mean values and 95% highest posterior density (HPD) intervals of hybrid rankings for canopy width (cm; 80 DAP). Hybrids with intervals in the bottom 15 are shown in blue and hybrids with intervals in the top 15 are shown in red.

6. Width (130 DAP)

6.1 GGE biplot

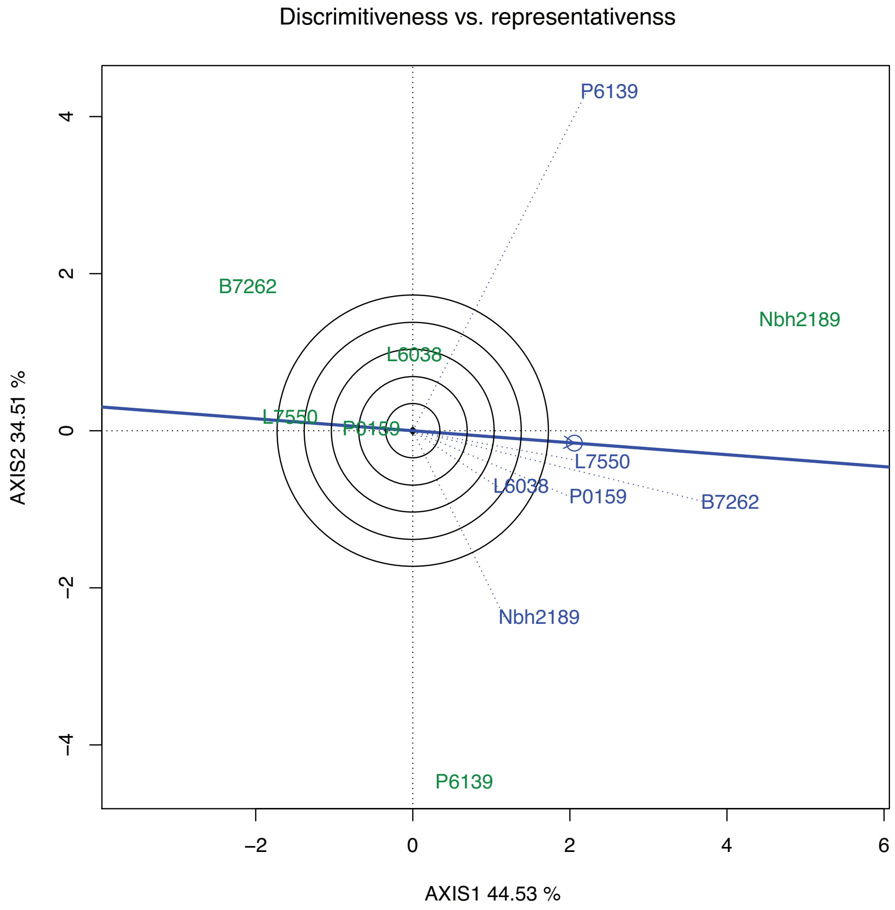

Figure S25: Discriminativeness vs. representativeness view of a biplot for canopy width at 130 days after planting. Parents treated as testers are shown in blue and as entries in green. The blue line indicates the average tester coordinate (ATC) abscissa, with the blue circle indicating the average tester score. The arrow in the circle indicates the direction of increasing GCA for the entries. Parent B7262 was both the most discriminating (had the longest vector) and was representative of other parental lines (vector was close to the ATC abscissa).

6.2 Observed and predicted values

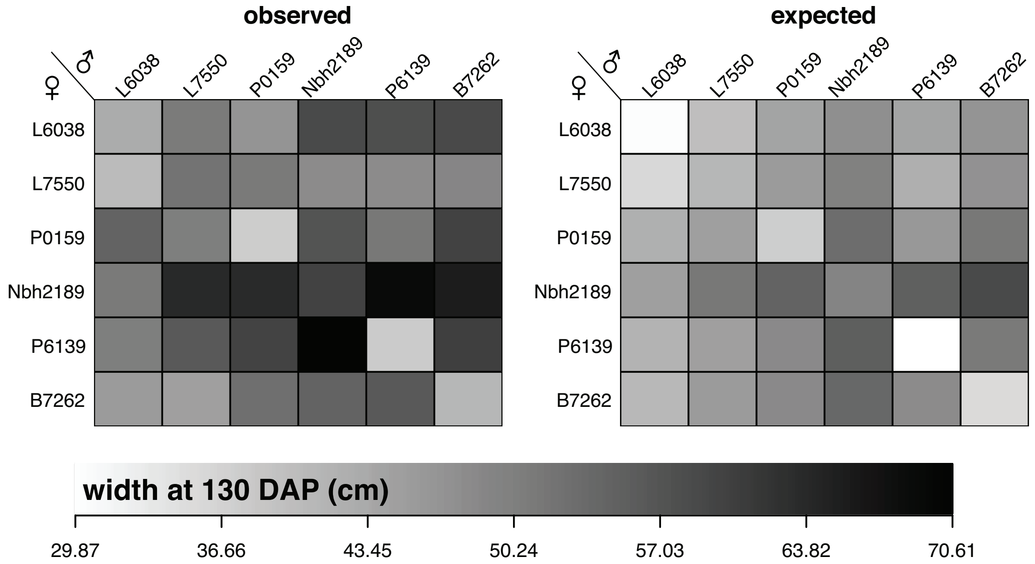

Figure S26: Observed (left) and predicted (right) means for width (130 DAP). Posterior predicted means on the right are the result of fitting the model in BayesDiallel to the data on the left.

6.3 HPD intervals for inheritance classes

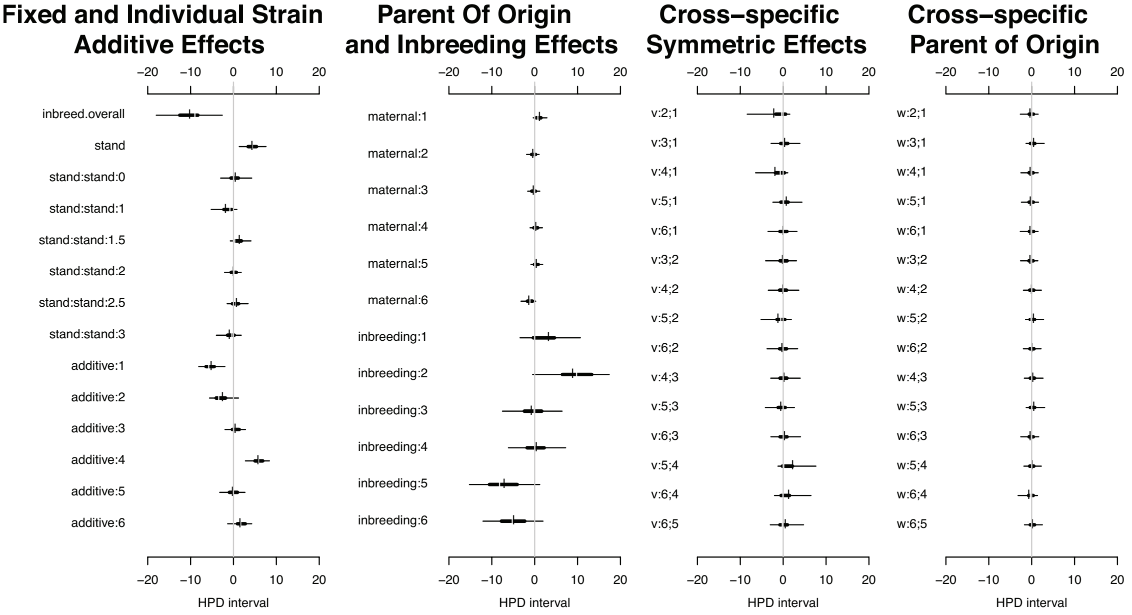

Figure S27: HPD intervals for canopy width (130 DAP; cm).

6.4 HPD intervals for fixed and random effects

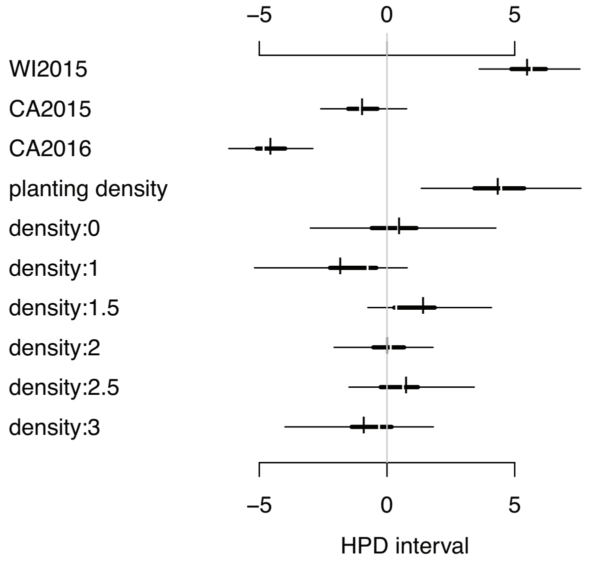

Figure S28: HPD intervals of fixed and random effects for canopy width (130 DAP; cm). Planting density ranged from 0 (low) to 3 (high).

6.5 Degree of dominance

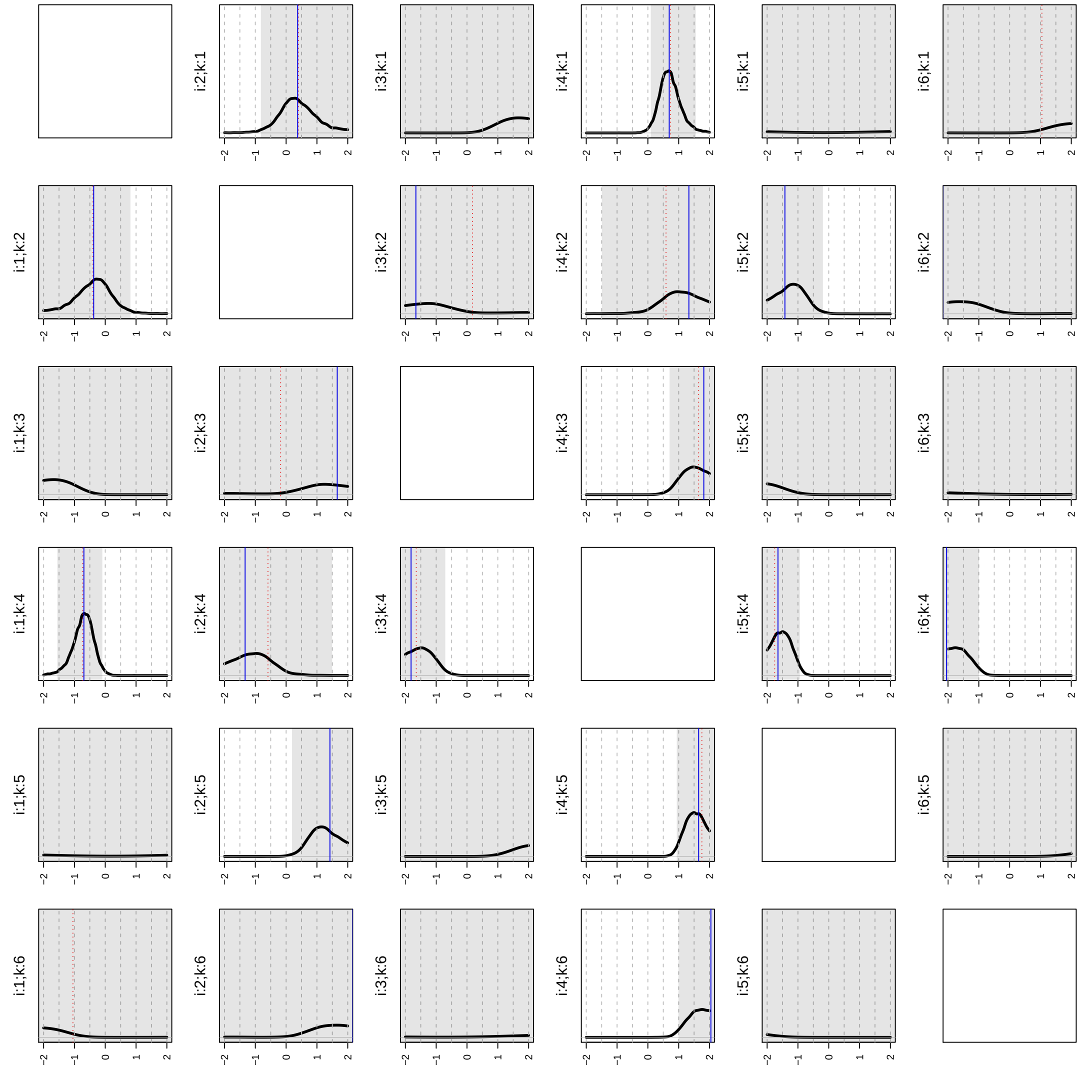

Figure S29: Posterior distributions of the degree of dominance for canopy width (130 DAP). Posterior means within the interval are indicated by red dotted lines and medians by blue solid lines, with 95% central quantiles shown in grey. A signal was considered strong if the mean, median, and the majority of the posterior distribution fell within the specified ranges: pseudo-underrecessive (-∞,-1.5), recessive (-1.5,-0.5), additive (-0.5,0.5), dominant (0.5,1.5), pseudo-overdominant (1.5,∞). Key for parental lines: 1=L6038, 2=L7550, 3=P0159, 4=Nbh2189, 5=P6139, 6=B7262.

6.6 GxE

6.6.1 Rankings by environment

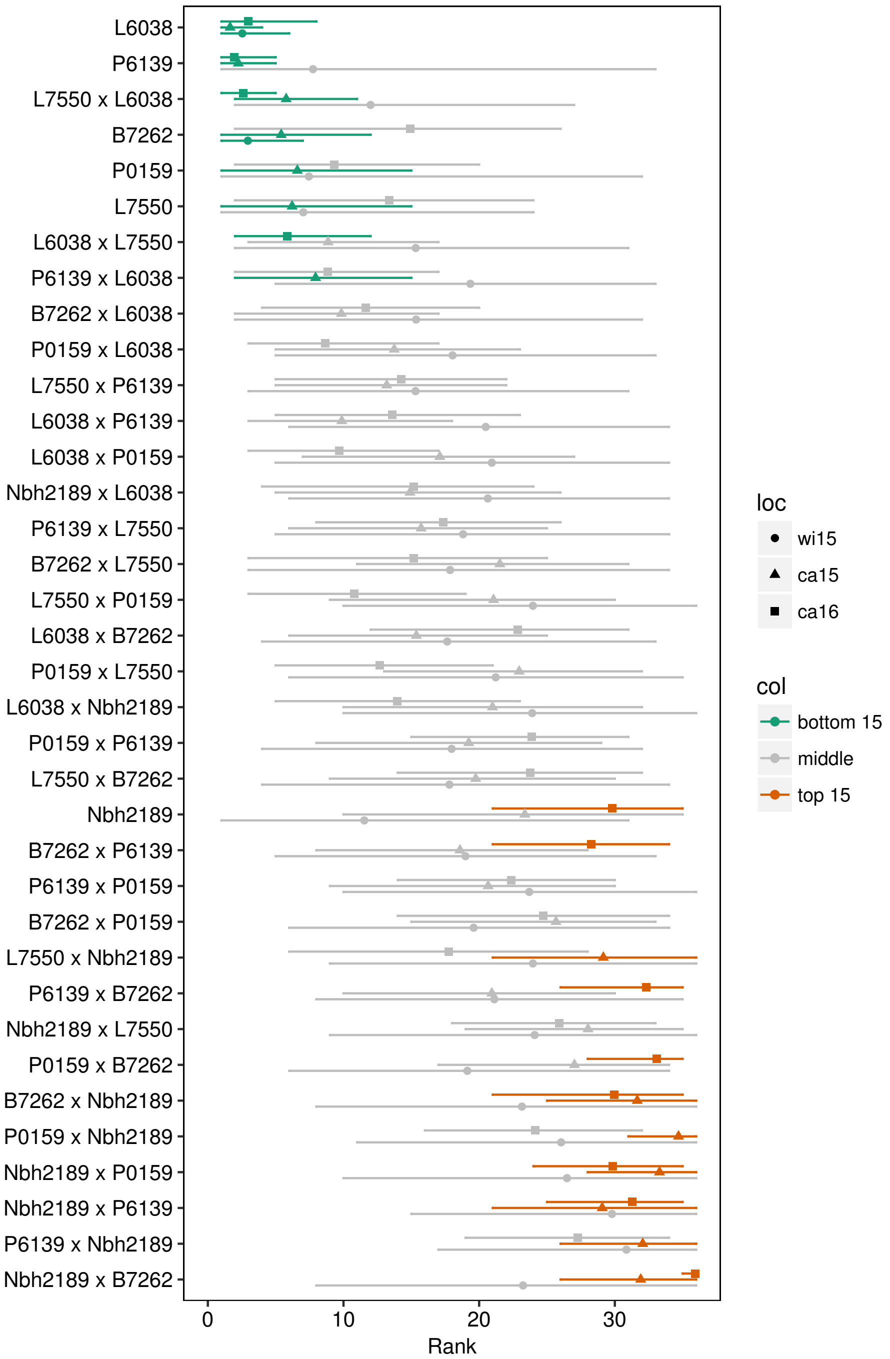

Figure S30: Mean values and 95% highest posterior density (HPD) intervals of hybrid rankings for canopy width (cm; 130 DAP). Hybrids with intervals in the bottom 15 are shown in blue and hybrids with intervals in the top 15 are shown in red.

7. Shoot biomass (fresh)

7.1 GGE biplot

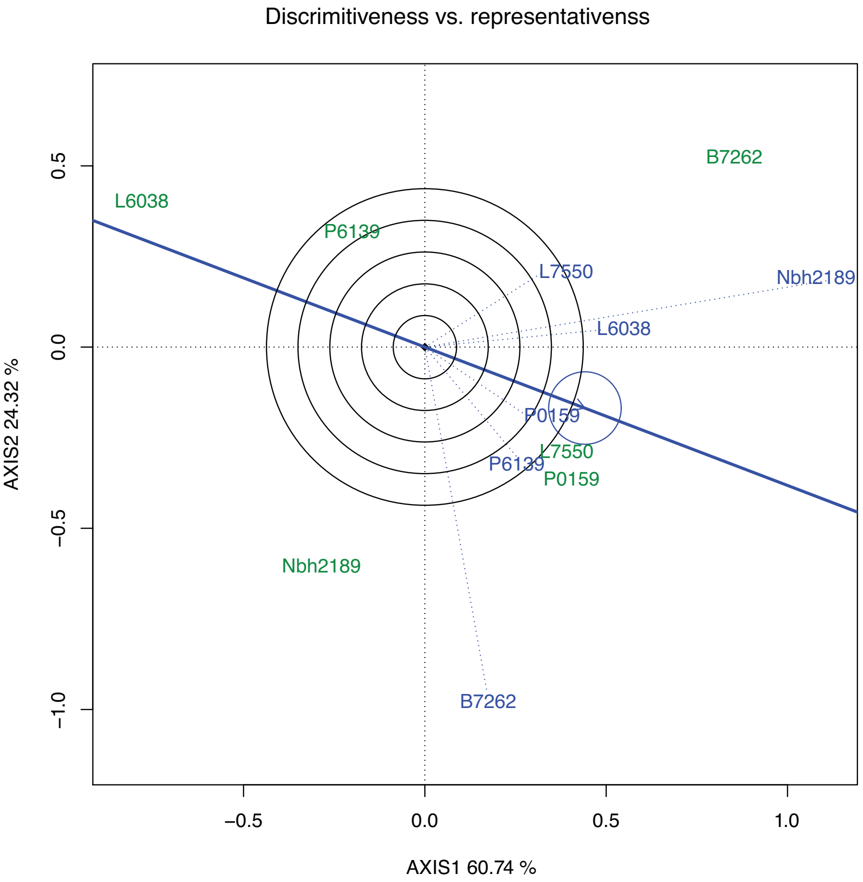

Figure S31: Discriminativeness vs. representativeness view of a biplot for fresh shoot biomass (measured 130 days after planting). Parents treated as testers are shown in blue and as entries in green. The blue line indicates the average tester coordinate (ATC) abscissa, with the blue circle indicating the average tester score. The arrow in the circle indicates the direction of increasing GCA for the entries. Parent P0159 was both the most discriminating (had the longest vector) and was representative of other parental lines (vector was close to the ATC abscissa).

7.2 Observed and predicted values

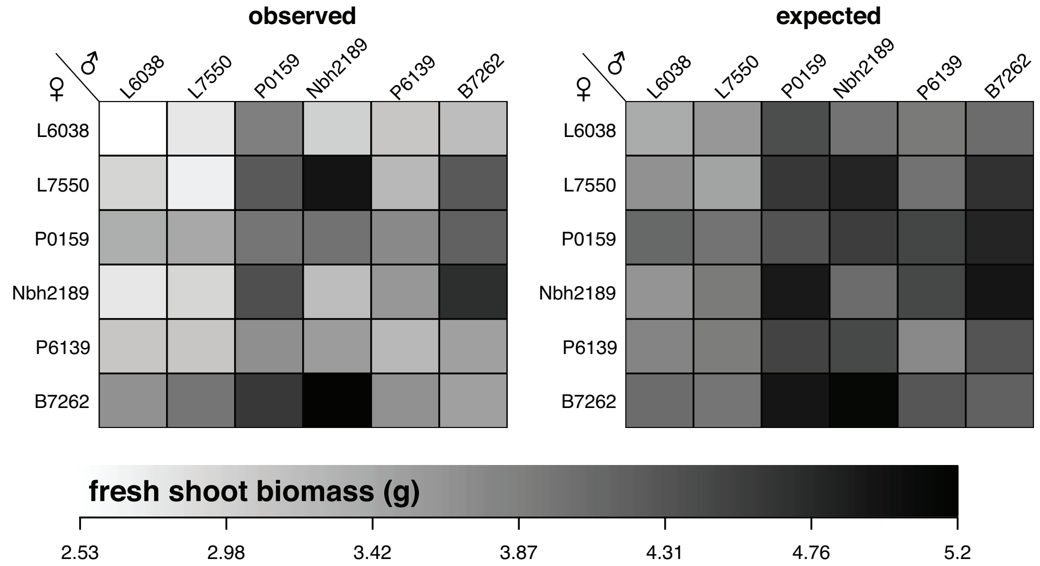

Figure S32: Observed (left) and predicted (right) means for fresh shoot biomass (g). Posterior predicted means on the right are the result of fitting the model in BayesDiallel to the data on the left.

7.3 HPD intervals for inheritance classes

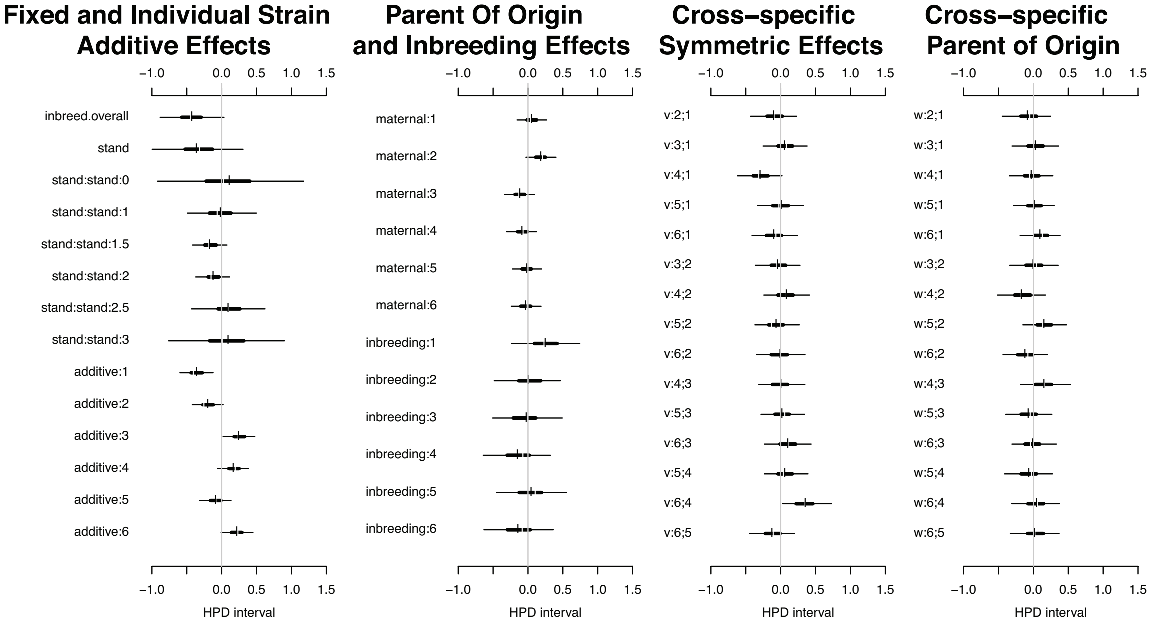

Figure S33: HPD intervals for fresh shoot biomass (g).

7.4 HPD intervals for fixed and random effects

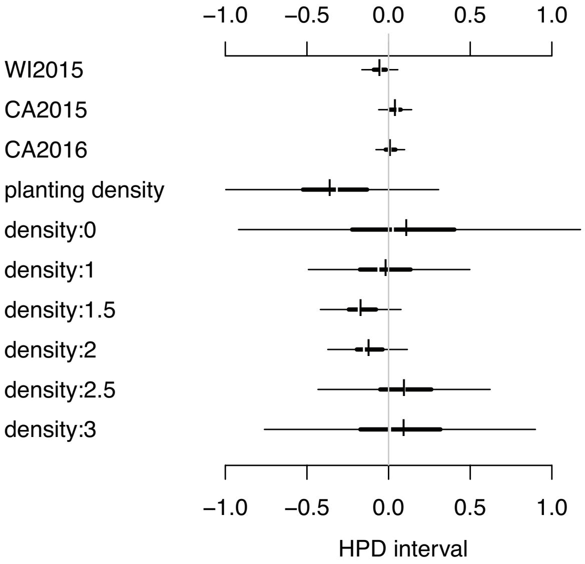

Figure S34: HPD intervals of fixed and random effects for fresh shoot biomass (g). Planting density ranged from 0 (low) to 3 (high).

7.5 Degree of dominance

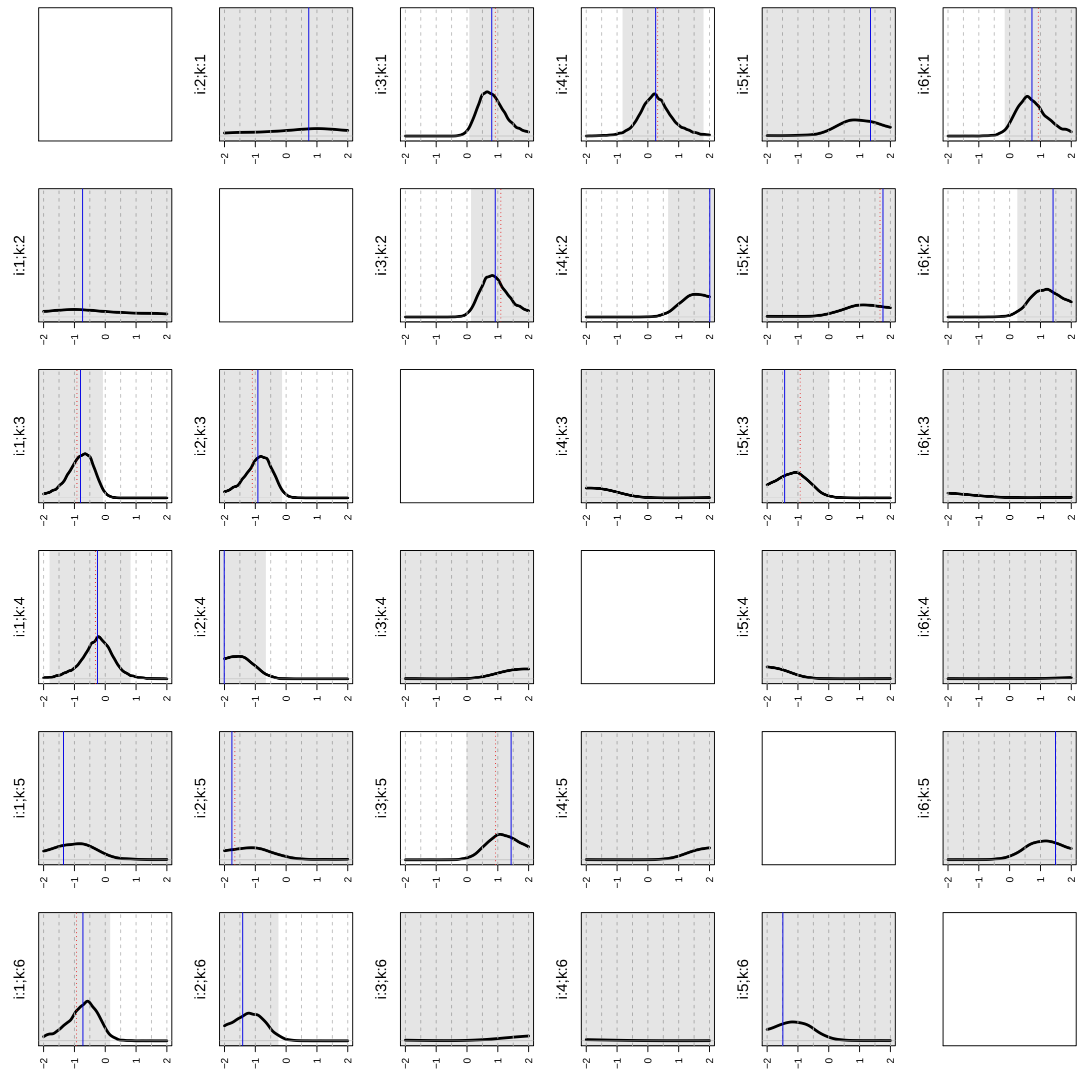

Figure S35: Posterior distributions of the degree of dominance for fresh shoot biomass. Posterior means within the interval are indicated by red dotted lines and medians by blue solid lines, with 95% central quantiles shown in grey. A signal was considered strong if the mean, median, and the majority of the posterior distribution fell within the specified ranges: pseudo-underrecessive (-∞,-1.5), recessive (-1.5,-0.5), additive (-0.5,0.5), dominant (0.5,1.5), pseudo-overdominant (1.5,∞). Key for parental lines: 1=L6038, 2=L7550, 3=P0159, 4=Nbh2189, 5=P6139, 6=B7262.

7.6 GxE

7.6.1 GCA x environment interactions

Table S10: GCA for fresh shoot biomass (g) in each growing environment.

Parent	WI2015	CA2015	CA2016
L6038	-0.32*	-0.67***	-0.42***
L7550	-0.15	-0.09	-0.28**
P0159	0.36**	0.38*	0.38***
Nbh2189	0.03	0.28	0.12
P6139	-0.11	-0.29*	-0.02
B7262	0.19	0.4**	0.22*

***P≤0.001, **P≤0.01, *P≤0.05

Table S11: Nonparametric correlations (Spearman's rho) among environments for GCA of fresh shoot biomass (g).

Environment	Spearman's rho
WI2015-CA2015	0.89*
WI2015-CA2016	1***
CA2015-CA2016	0.89*

***P≤0.001, **P≤0.01, *P≤0.05

7.6.2 SCA x environment interactions

Table S12: SCA for fresh shoot biomass (g) in each growing environment.

F1 hybrids	WI2015	CA2015	CA2016
L6038 x L7550	0.3	-0.15	0
L6038 x P0159	-0.05	0.19	0.17
L6038 x Nbh2189	0.36	-0.53*	-0.5*
L6038 x P6139	0.13	0.21	0.16
L6038 x B7262	-0.23	-0.03	0.02
L7550 x P0159	0.17	-0.13	0.16
L7550 x Nbh2189	0.4	0.29	0.1
L7550 x P6139	-0.3	0.18	0.1
L7550 x B7262	0.14	0.4	0.13
P0159 x Nbh2189	0	0.1	0.07
P0159 x P6139	-0.29	0.13	-0.12
P0159 x B7262	-0.17	0	-0.08
Nbh2189 x P6139	0.37	-0.18	0.17
Nbh2189 x B7262	0.4	1.01***	0.29
P6139 x B7262	-0.19	-0.37	-0.03
Parental selfs			
L6038	-0.54	0.31	0.08
L7550	-0.22	-0.61	-0.24
P0159	-0.38	-0.29	-0.22
Nbh2189	-0.53	-0.68*	-0.06
P6139	-0.1	0.02	-0.35
B7262	-0.73	-1.01**	-0.41

***P≤0.001, **P≤0.01, *P≤0.05

Table S13: Nonparametric correlations (Spearman's rho) among environments for SCA of fresh shoot biomass (g).

Environment	Spearman's rho
WI2015-CA2015	0.32
WI2015-CA2016	0.46*
CA2015-CA2016	0.66**

***P≤0.001, **P≤0.01, *P≤0.05

7.6.3 Reciprocal x environment interactions

Table S14: Reciprocal effects for fresh shoot biomass (g) in each location.

F1 hybrids	WI2015	CA2015	CA2016
L6038 x L7550	-0.17	-0.05	-0.04
L6038 x P0159	0	0.23	0.31*
L6038 x Nbh2189	0.25*	0.24*	-0.12
L6038 x P6139	0.26*	-0.1	-0.16
L6038 x B7262	-0.02	-0.08	-0.17
L7550 x L6038	0.17	0.05	0.04
L7550 x P0159	0.83***	0.5***	0.2
L7550 x Nbh2189	0.54**	1.09***	0.53**
L7550 x P6139	-0.13	0.19	0.19
L7550 x B7262	0.23	0.79***	-0.12
P0159 x L6038	0	-0.23	-0.31*
P0159 x L7550	-0.83***	-0.5***	-0.2
P0159 x Nbh2189	0.06	-0.2	-0.01
P0159 x P6139	-0.09	0.01	0.15
P0159 x B7262	-0.1	-0.03	0.05
Nbh2189 x L6038	-0.25*	-0.24*	0.12
Nbh2189 x L7550	-0.54**	-1.09***	-0.53**
Nbh2189 x P0159	-0.06	0.2	0.01
Nbh2189 x P6139	0.19	-0.08	-0.02
Nbh2189 x B7262	-0.29	-0.27*	-0.24*
P6139 x L6038	-0.26*	0.1	0.16
P6139 x L7550	0.13	-0.19	-0.19
P6139 x P0159	0.09	-0.01	-0.15
P6139 x Nbh2189	-0.19	0.08	0.02
P6139 x B7262	0.16	-0.1	-0.16
B7262 x L6038	0.02	0.08	0.17
B7262 x L7550	-0.23	-0.79***	0.12
B7262 x P0159	0.1	0.03	-0.05
B7262 x Nbh2189	0.29	0.27*	0.24*
B7262 x P6139	-0.16	0.1	0.16

***P≤0.001, **P≤0.01, *P≤0.05

Table S15: Nonparametric correlations (Spearman's rho) among environments for reciprocal effects of fresh shoot biomass (g).

Environment	Spearman's rho
WI2015-CA2015	0.45**
WI2015-CA2016	0.09
CA2015-CA2016	0.65***

***P≤0.001, **P≤0.01, *P≤0.05

7.6.4 Rankings by environment

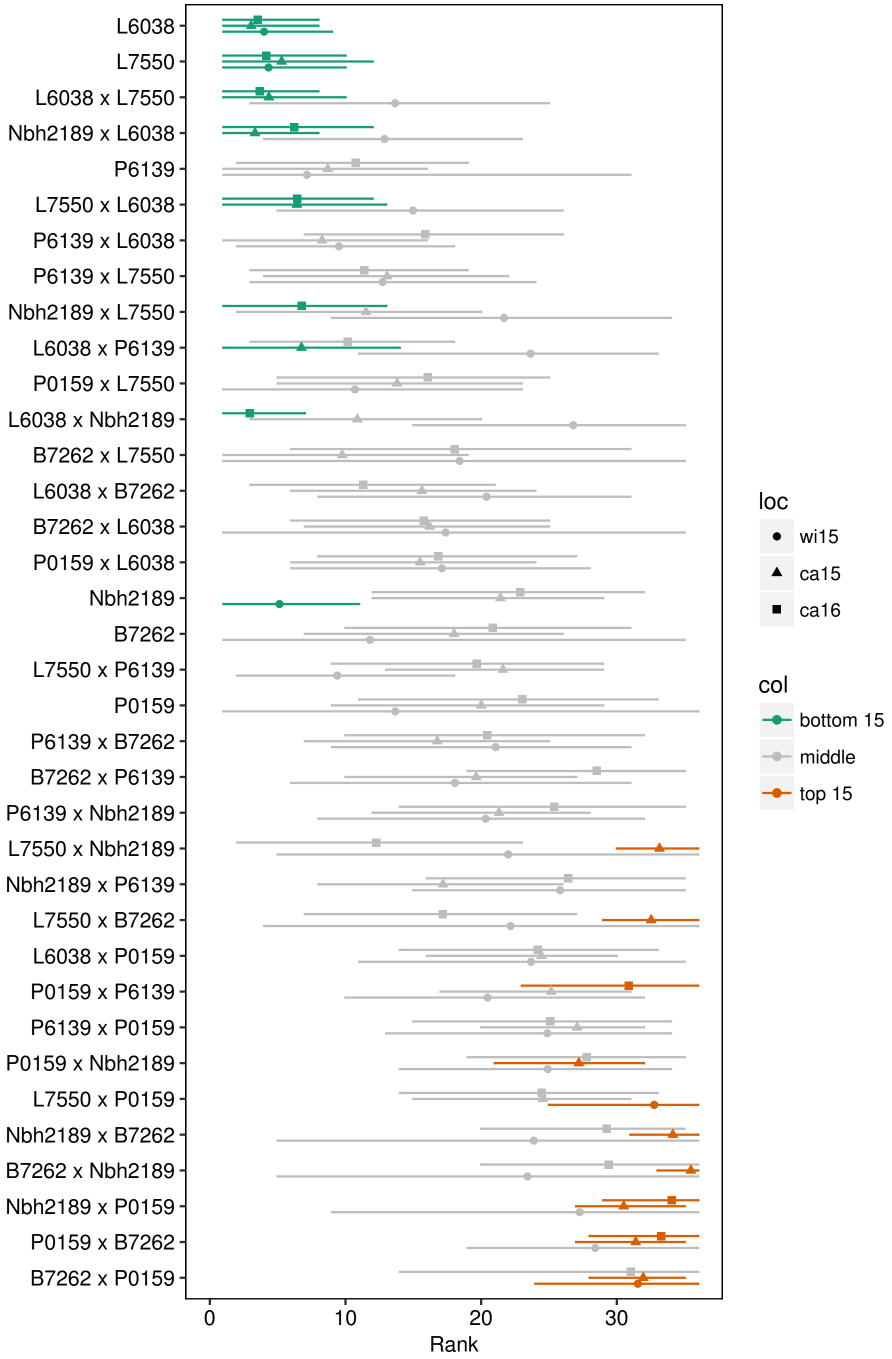

Figure S36: Mean values and 95% highest posterior density (HPD) intervals of hybrid rankings for fresh shoot biomass (g; 130 DAP). Hybrids with intervals in the bottom 15 are shown in blue and hybrids with intervals in the top 15 are shown in red.

8. Shoot biomass (dry)

8.1 GGE biplot

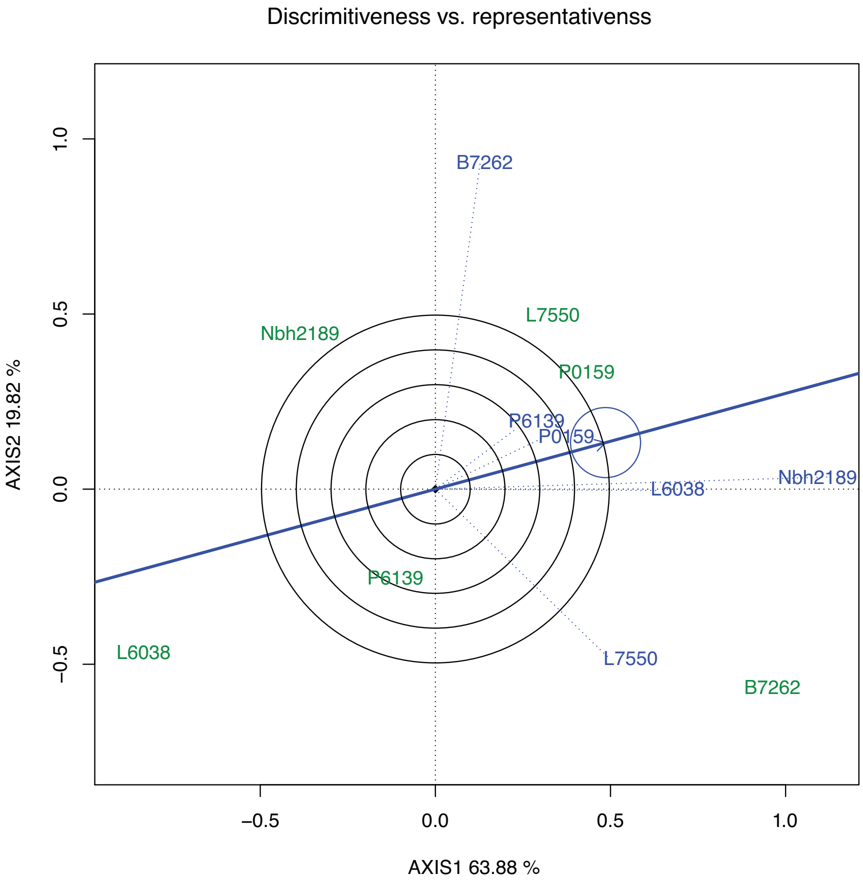

Figure S37: Discriminativeness vs. representativeness view of a biplot for dry shoot biomass (measured 130 days after planting). Parents treated as testers are shown in blue and as entries in green. The blue line indicates the average tester coordinate (ATC) abscissa, with the blue circle indicating the average tester score. The arrow in the circle indicates the direction of increasing GCA for the entries. Parent Nbh2189 was the most discriminating (had the longest vector) and parent P0159 was the most representative of other parental lines (vector was close to the ATC abscissa).

8.2 Observed and predicted values

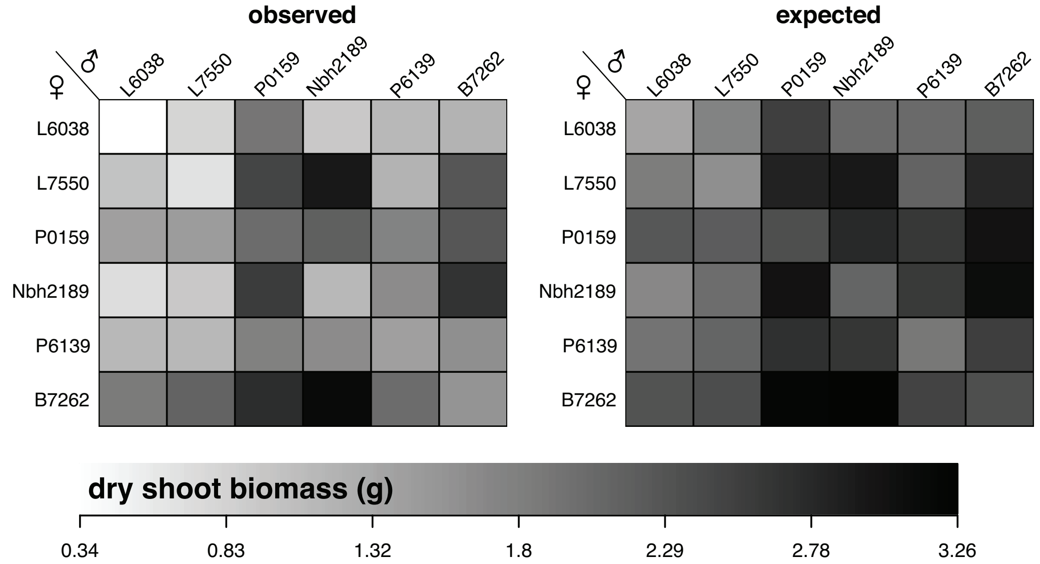

Figure S38: Observed (left) and predicted (right) means for dry shoot biomass (g). Posterior predicted means on the right are the result of fitting the model in BayesDiallel to the data on the left.

8.3 HPD intervals for inheritance classes

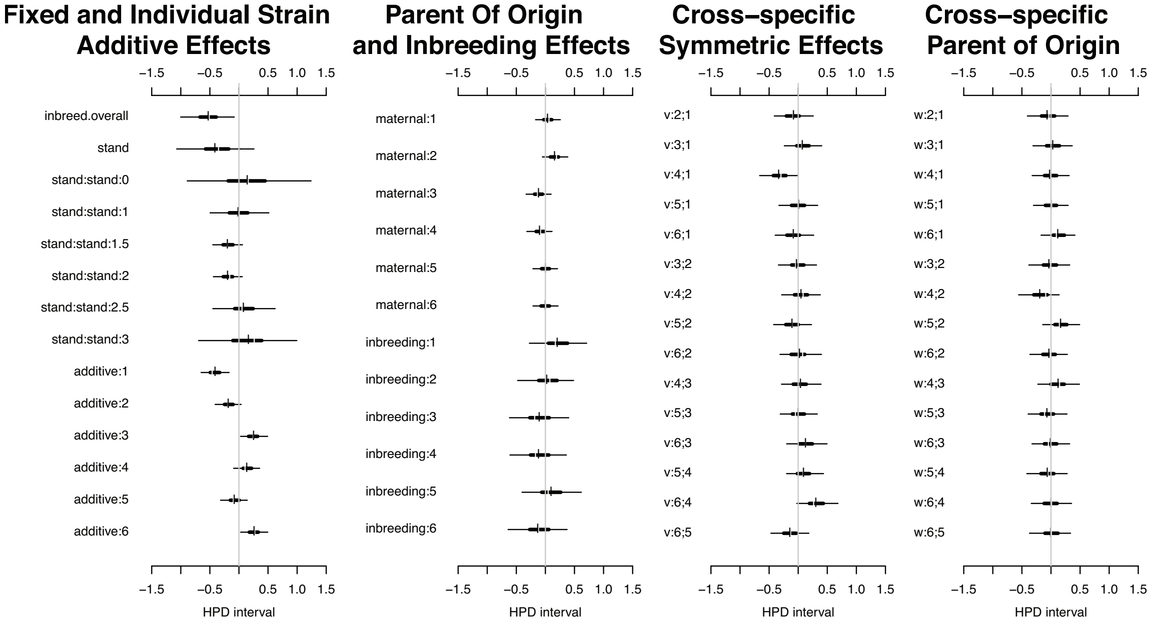

Figure S39: HPD intervals for dry shoot biomass (g).

8.4 HPD intervals for fixed and random effects

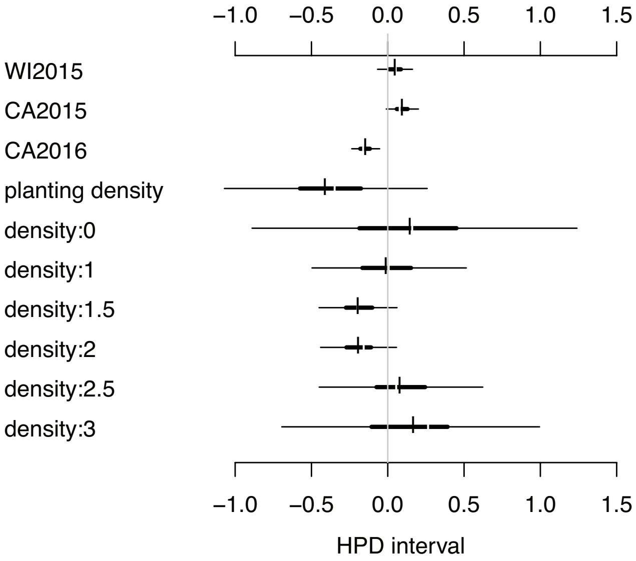

Figure S40: HPD intervals of fixed and random effects for dry shoot biomass (g). Planting density ranged from 0 (low) to 3 (high).

8.5 Degree of dominance

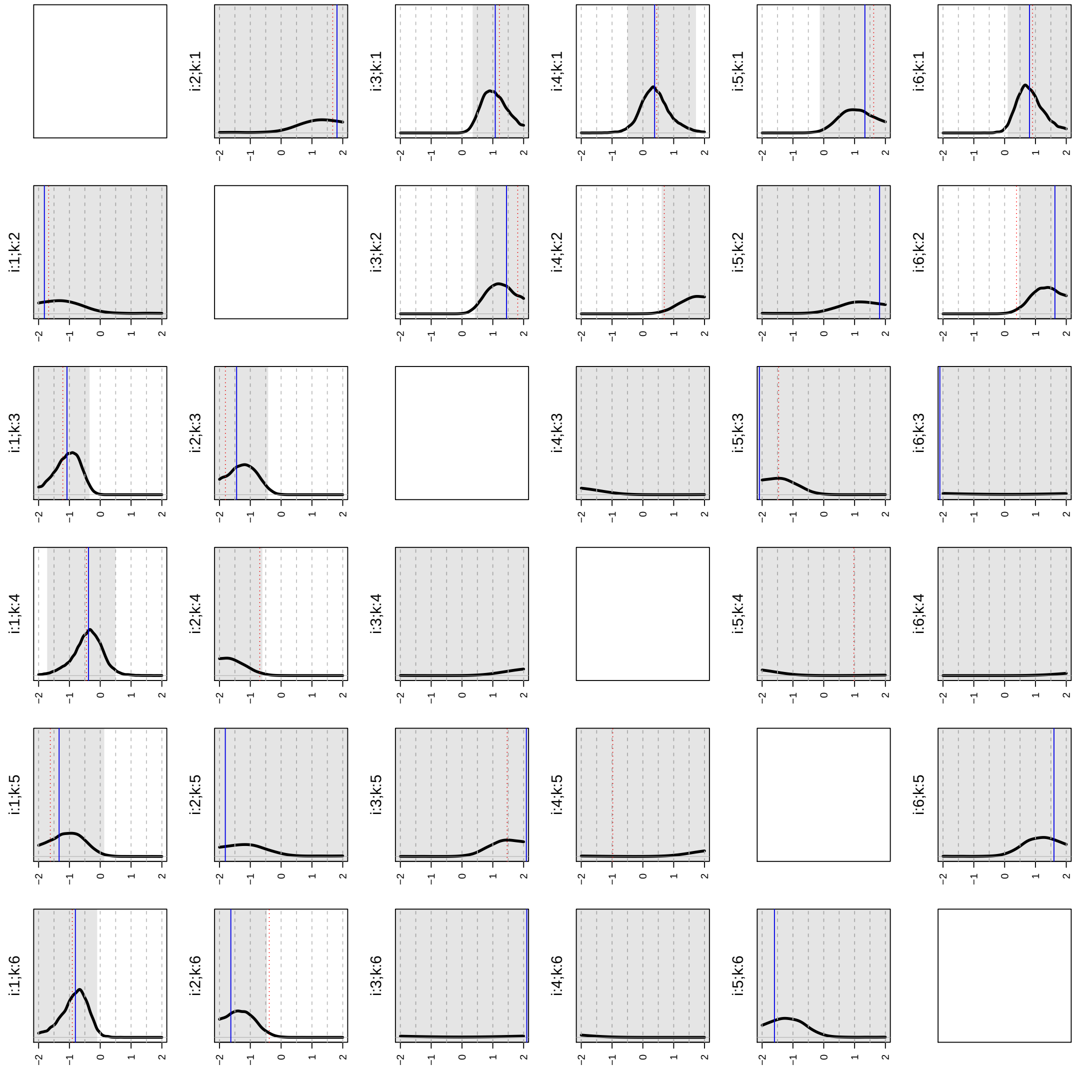

Figure S41: Posterior distributions of the degree of dominance for dry shoot biomass. Posterior means within the interval are indicated by red dotted lines and medians by blue solid lines, with 95% central quantiles shown in grey. A signal was considered strong if the mean, median, and the majority of the posterior distribution fell within the specified ranges: pseudo-underrecessive (-∞,-1.5), recessive (-1.5,-0.5), additive (-0.5,0.5), dominant (0.5,1.5), pseudo-overdominant (1.5,∞). Key for parental lines: 1=L6038, 2=L7550, 3=P0159, 4=Nbh2189, 5=P6139, 6=B7262.

8.6 GxE

8.6.1 GCA x environment interactions

Table S16: GCA for dry shoot biomass (g) in each growing environment.

Parent	WI2015	CA2015	CA2016
L6038	-0.41**	-0.61***	-0.56***
L7550	-0.14	-0.03	-0.28*
P0159	0.43**	0.31*	0.43***
Nbh2189	-0.04	0.24	0.13
P6139	-0.12	-0.3*	-0.01
B7262	0.29	0.4**	0.3*

***P≤0.001, **P≤0.01, *P≤0.05

Table S17: Nonparametric correlations (Spearman's rho) among environments for GCA of dry shoot biomass (g).

Environment	Spearman's rho
WI2015-CA2015	0.89*
WI2015-CA2016	1***
CA2015-CA2016	0.89*

***P≤0.001, **P≤0.01, *P≤0.05

8.6.2 SCA x environment interactions

Table S18: SCA for dry shoot biomass (g) in each growing environment.

F1 hybrids	WI2015	CA2015	CA2016
L6038 x L7550	0.49*	-0.14	-0.04
L6038 x P0159	-0.02	0.17	0.22
L6038 x Nbh2189	0.42	-0.52*	-0.61**
L6038 x P6139	0.17	0.25	0.23
L6038 x B7262	-0.23	-0.04	0.08
L7550 x P0159	0.15	-0.08	0.22
L7550 x Nbh2189	0.48	0.25	0.1
L7550 x P6139	-0.38	0.11	0.11
L7550 x B7262	0.2	0.53*	0.24
P0159 x Nbh2189	0.04	0.12	0.12
P0159 x P6139	-0.38	0.13	-0.17
P0159 x B7262	-0.28	-0.01	-0.05
Nbh2189 x P6139	0.43	-0.14	0.21
Nbh2189 x B7262	0.36	0.93***	0.32
P6139 x B7262	-0.24	-0.35	-0.08
Parental selfs			
L6038	-0.79*	0.28	0.04
L7550	-0.34	-0.67*	-0.31
P0159	-0.63	-0.32	-0.32
Nbh2189	-0.64	-0.64	-0.13
P6139	-0.08	0.01	-0.39
B7262	-0.86*	-1.06**	-0.5

***P≤0.001, **P≤0.01, *P≤0.05

Table S19: Nonparametric correlations (Spearman's rho) among environments for SCA of dry shoot biomass (g).

Environment	Spearman's rho
WI2015-CA2015	0.30
WI2015-CA2016	0.48*
CA2015-CA2016	0.70**

***P≤0.001, **P≤0.01, *P≤0.05

8.6.3 Rankings by environment

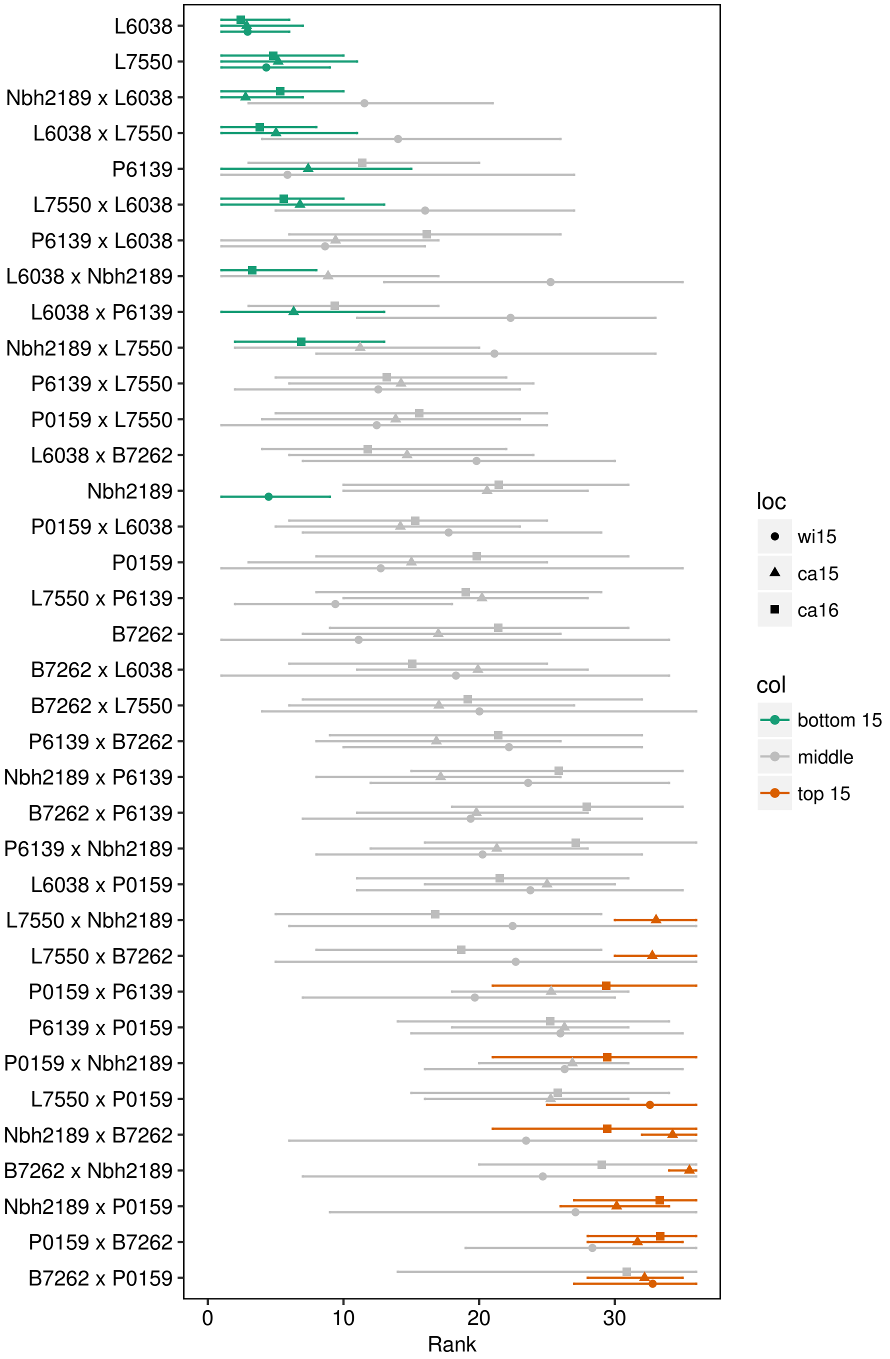

Figure S42: Mean values and 95% highest posterior density (HPD) intervals of hybrid rankings for dry shoot biomass (g; 130 DAP). Hybrids with intervals in the bottom 15 are shown in blue and hybrids with intervals in the top 15 are shown in red.

9. Root biomass (fresh)

9.1 GGE biplot

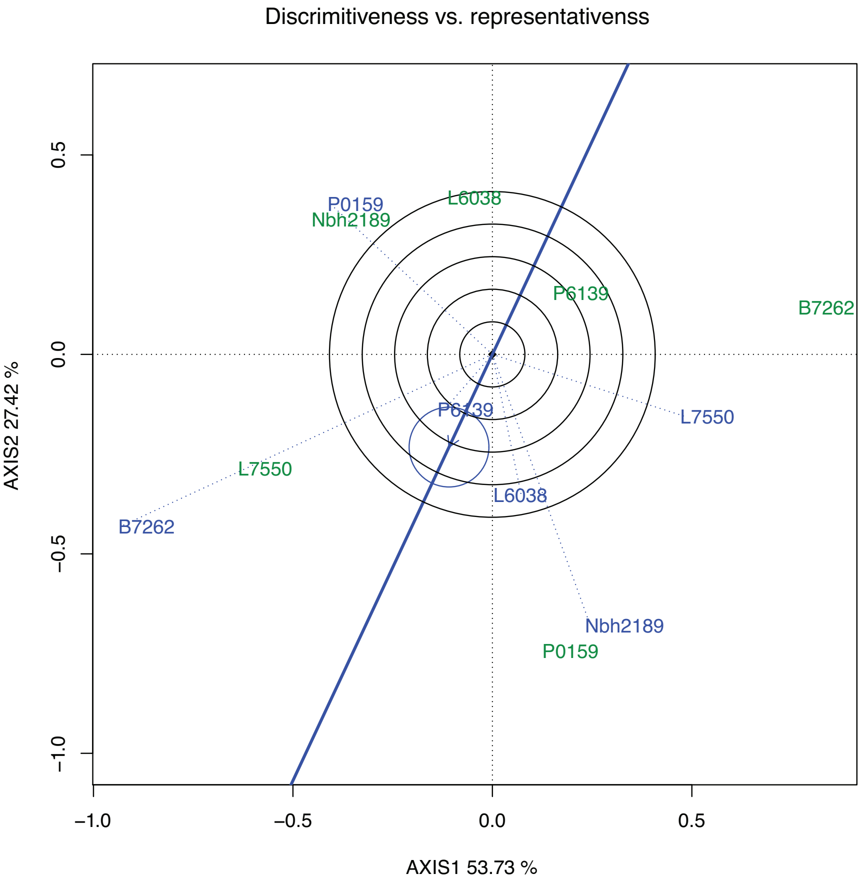

Figure S43: Discriminativeness vs. representativeness view of a biplot for fresh root biomass (measured 130 days after planting). Parents treated as testers are shown in blue and as entries in green. The blue line indicates the average tester coordinate (ATC) abscissa, with the blue circle indicating the average tester score. The arrow in the circle indicates the direction of increasing GCA for the entries. Parent Nbh2189 was both discriminating (had the longest vector) and was moderately representative of other parental lines (vector was close to the ATC abscissa), although parent P6139 was the most representative.

9.2 Observed and predicted values

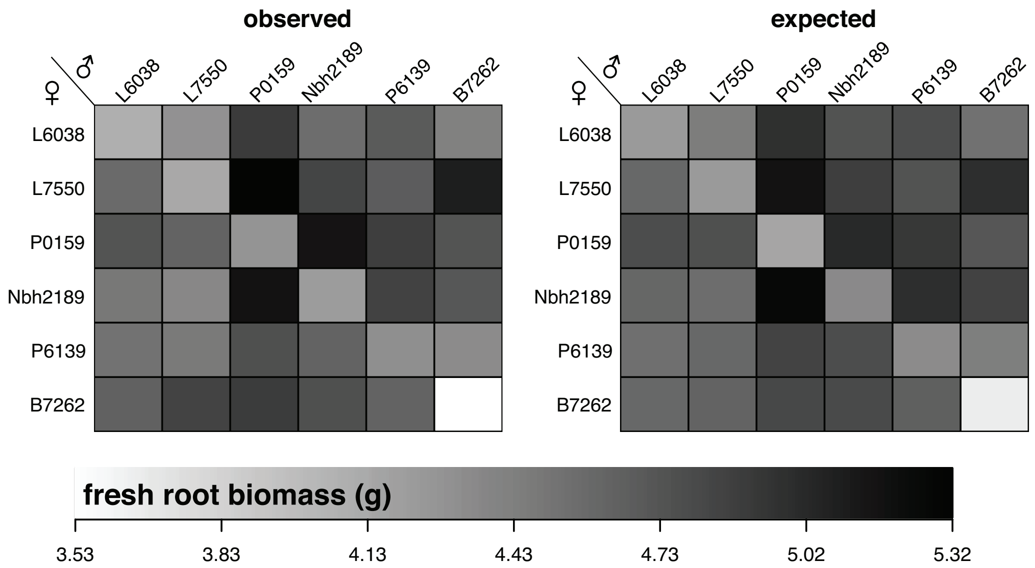

Figure S44: Observed (left) and predicted (right) means for root biomass (fresh; g). Posterior predicted means on the right are the result of fitting the model in BayesDiallel to the data on the left.

9.3 HPD intervals for inheritance classes

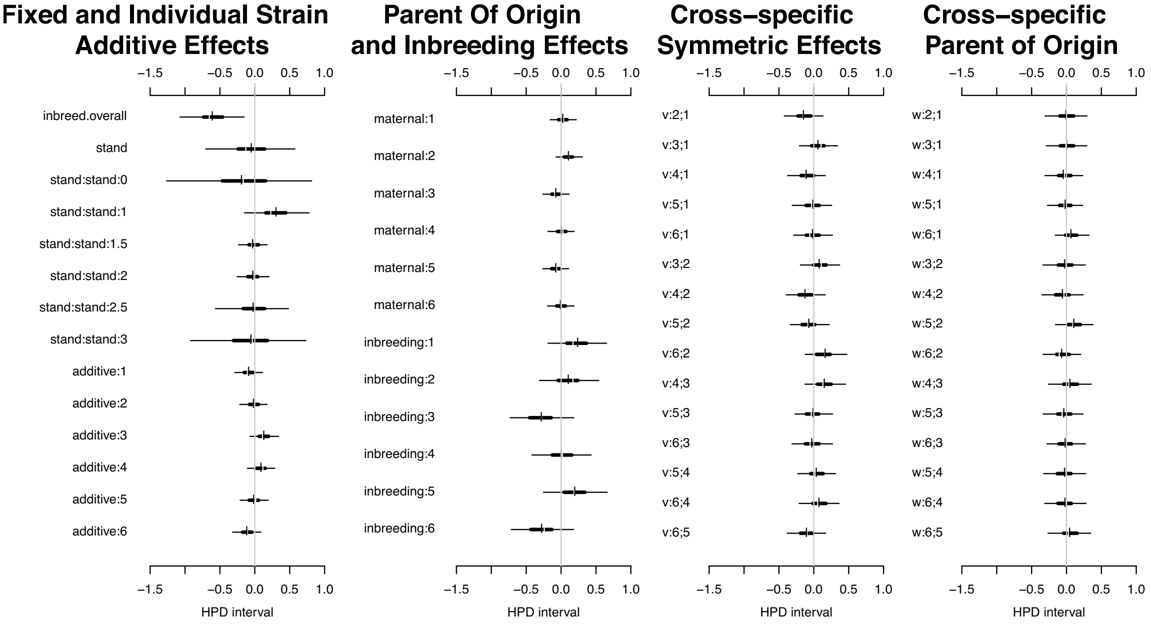

Figure S45: HPD intervals for fresh root biomass (g).

9.4 HPD intervals for fixed and random effects

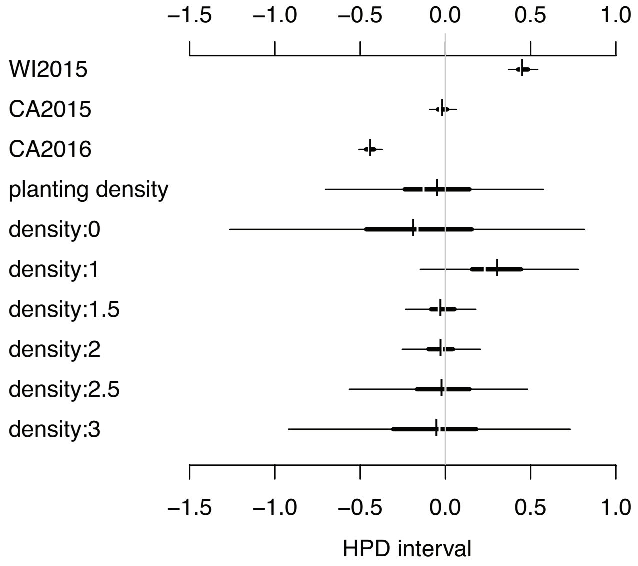

Figure S46: HPD intervals of fixed and random effects for fresh root biomass (g). Planting density ranged from 0 (low) to 3 (high).

9.5 Degree of dominance

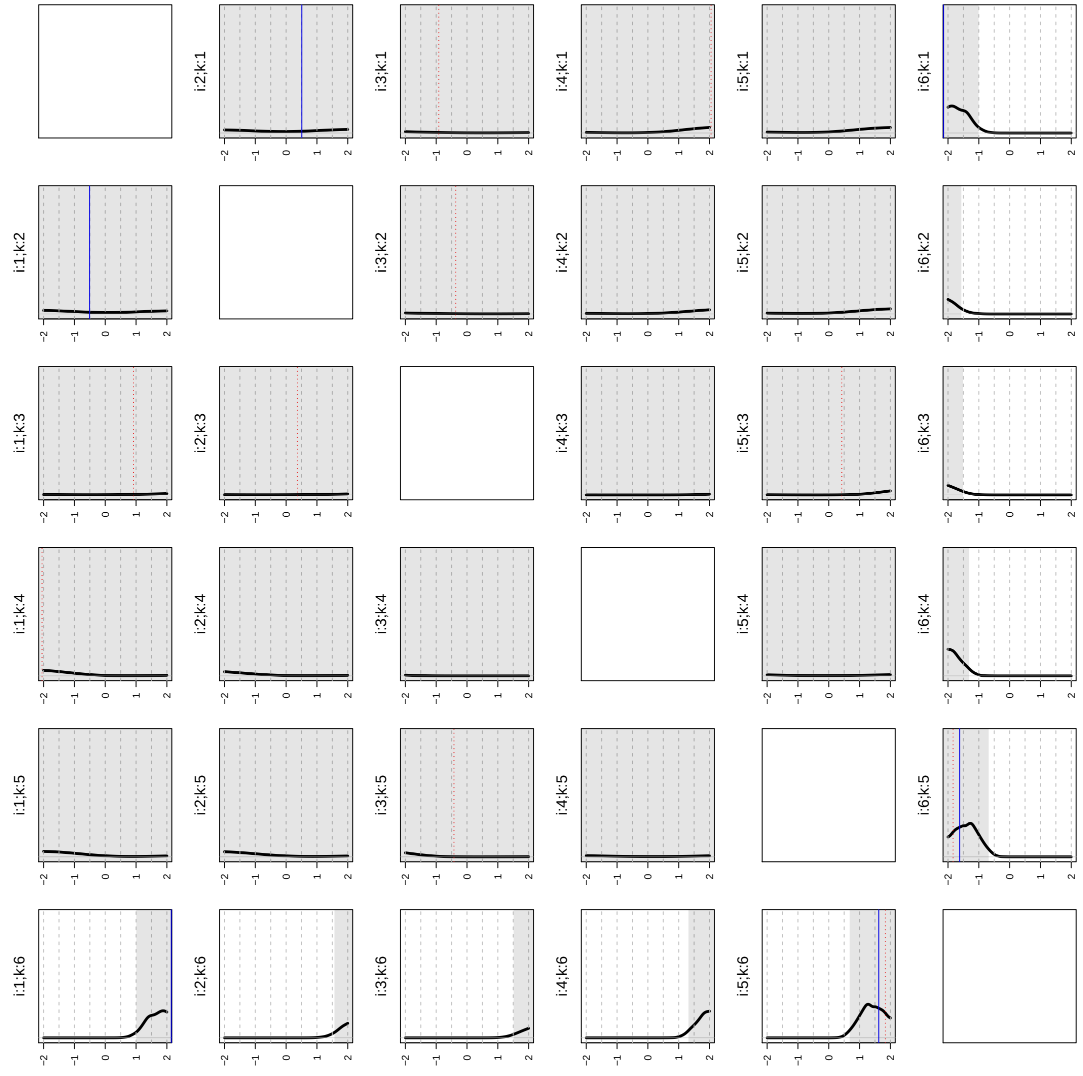

Figure S47: Posterior distributions of the degree of dominance for fresh root biomass. Posterior means within the interval are indicated by red dotted lines and medians by blue solid lines, with 95% central quantiles shown in grey. A signal was considered strong if the mean, median, and the majority of the posterior distribution fell within the specified ranges: pseudo-underrecessive (-∞,-1.5), recessive (-1.5,-0.5), additive (-0.5,0.5), dominant (0.5,1.5), pseudo-overdominant (1.5,∞). Key for parental lines: 1=L6038, 2=L7550, 3=P0159, 4=Nbh2189, 5=P6139, 6=B7262.

9.6 GxE

9.6.1 Rankings by environment

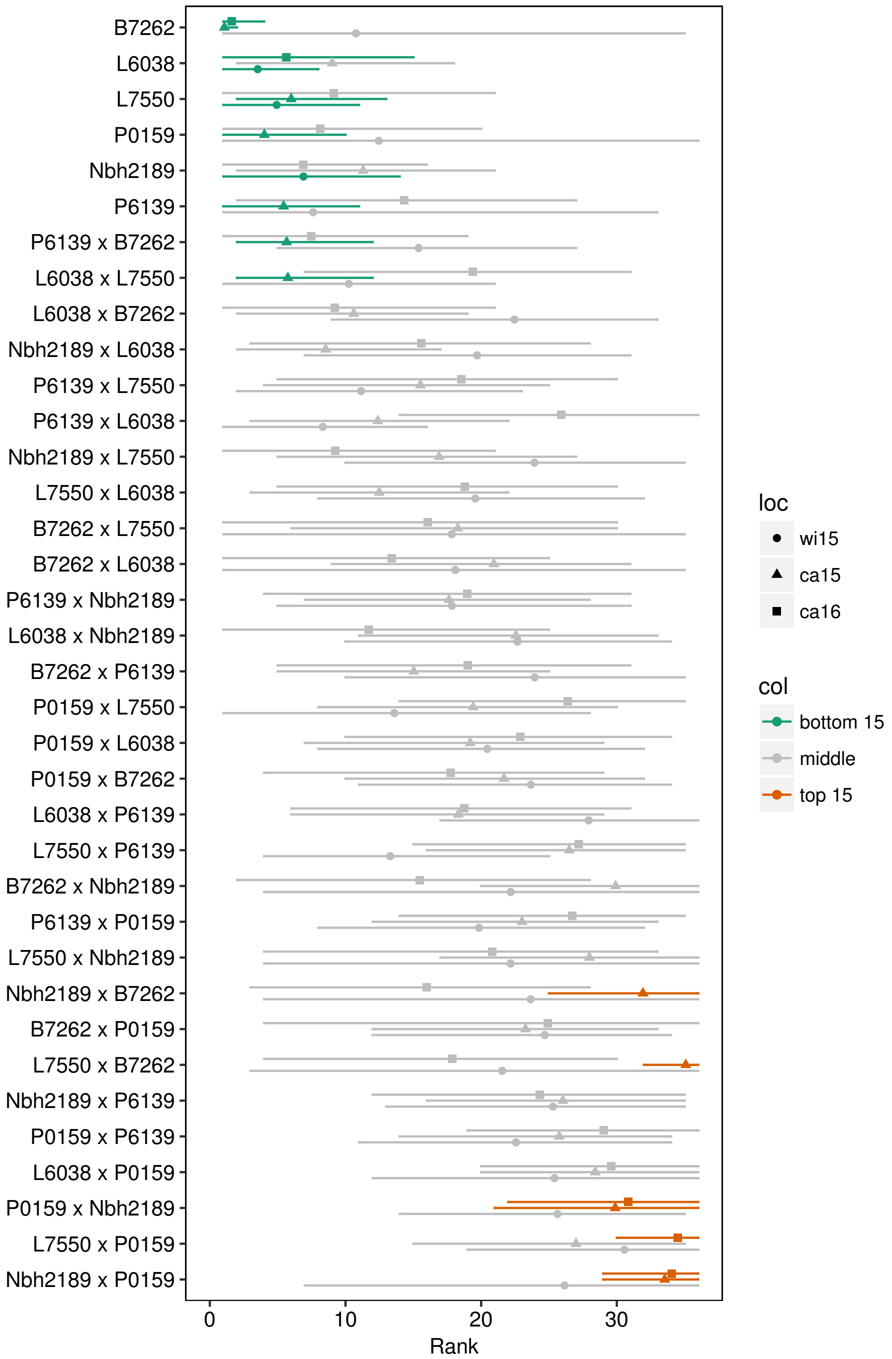

Figure S48: Mean values and 95% highest posterior density (HPD) intervals of hybrid rankings for fresh root biomass (g; 130 DAP). Hybrids with intervals in the bottom 15 are shown in blue and hybrids with intervals in the top 15 are in red.

10. Root biomass (dry)

10.1 GGE biplot

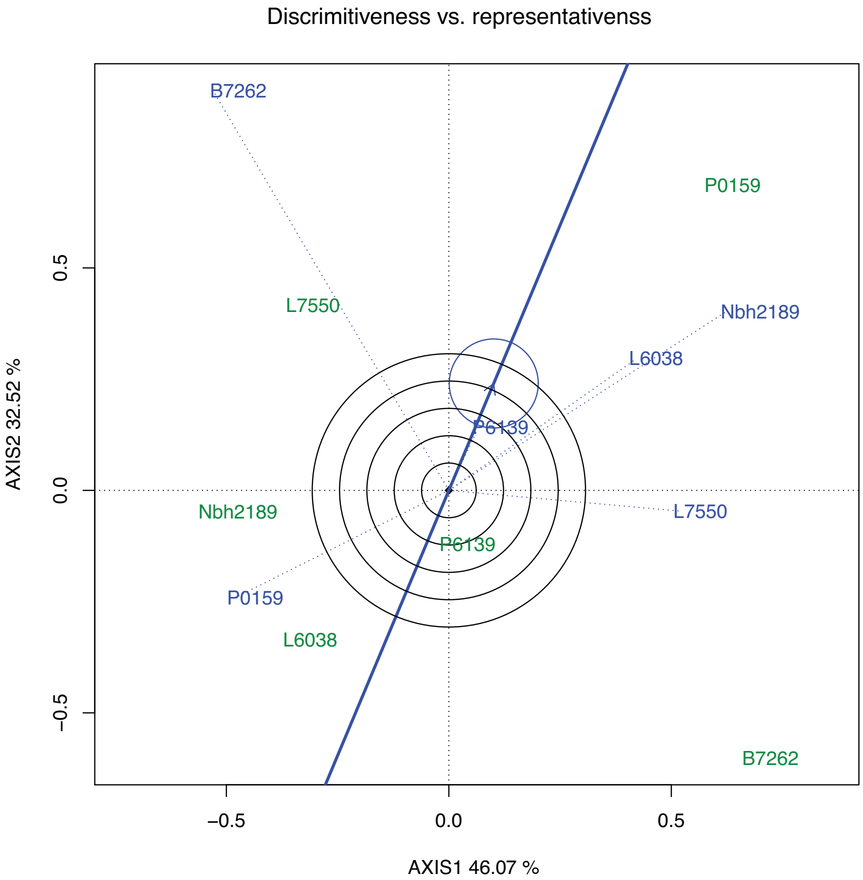

Figure S49: Discriminativeness vs. representativeness view of a biplot for dry root biomass (measured 130 days after planting). Parents treated as testers are shown in blue and as entries in green. The blue line indicates the average tester coordinate (ATC) abscissa, with the blue circle indicating the average tester score. The arrow in the circle indicates the direction of increasing GCA for the entries. Parent Nbh2189 was both discriminating (had the longest vector) and was moderately representative of other parental lines (vector was close to the ATC abscissa), although parent P6139 was the most representative.

10.2 Observed and predicted values

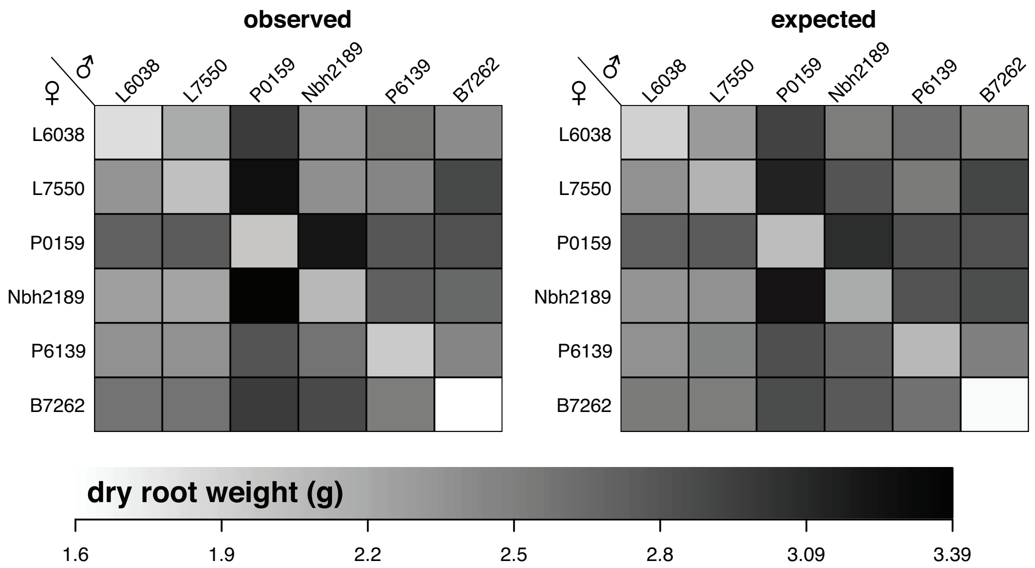

Figure S50: Observed (left) and predicted (right) means for dry root biomass (g). Posterior predicted means on the right are the result of fitting the model in BayesDiallel to the data on the left.

10.3 HPD intervals for inheritance classes

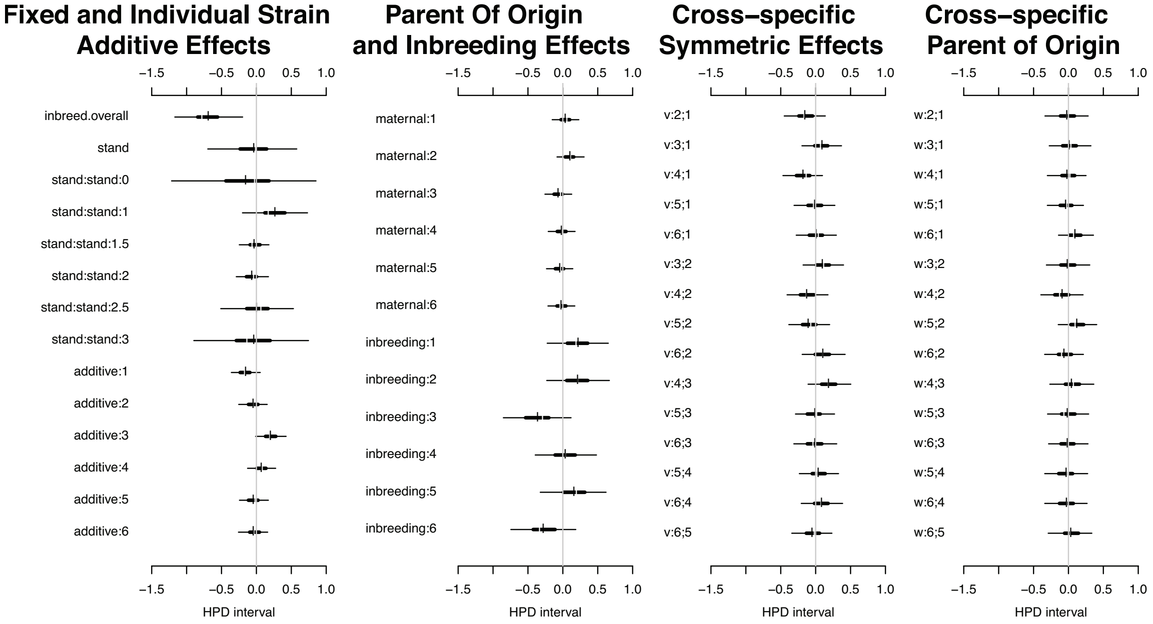

Figure S51: HPD intervals for dry root biomass (g).

10.4 HPD intervals for fixed and random effects

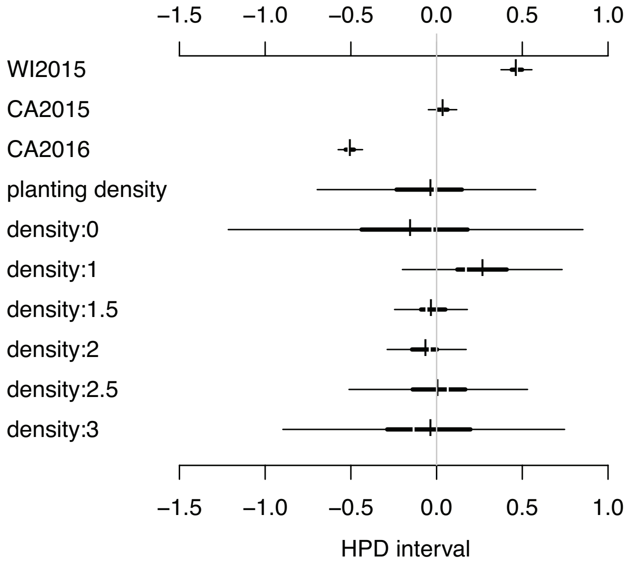

Figure S52: HPD intervals of fixed and random effects for dry root biomass (g). Planting density ranged from 0 (low) to 3 (high).

10.5 Degree of dominance

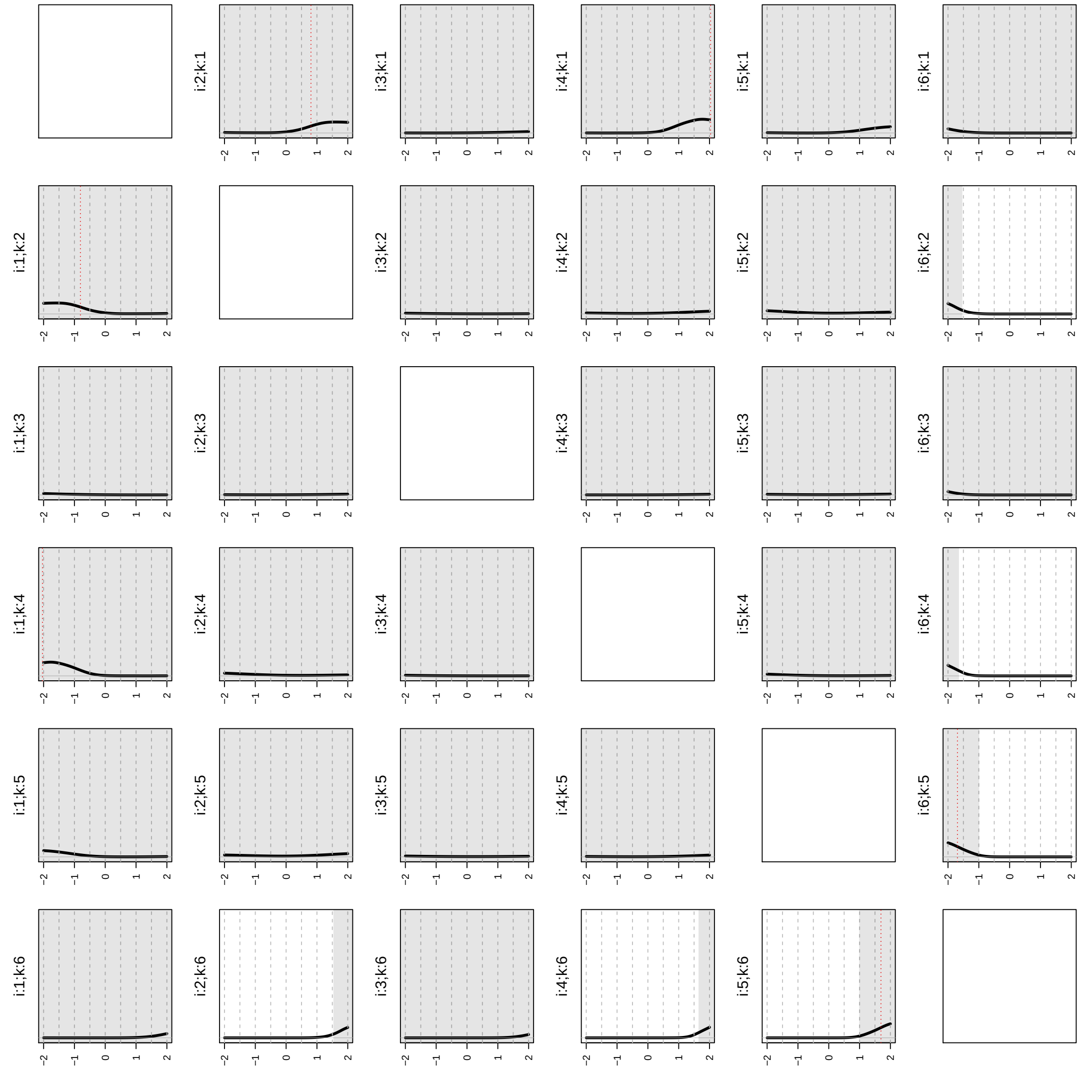

Figure S53: Posterior distributions of the degree of dominance for dry root biomass. Posterior means within the interval are indicated by red dotted lines and medians by blue solid lines, with 95% central quantiles shown in grey. A signal was considered strong if the mean, median, and the majority of the posterior distribution fell within the specified ranges: pseudo-underrecessive (-∞,-1.5), recessive (-1.5,-0.5), additive (-0.5,0.5), dominant (0.5,1.5), pseudo-overdominant (1.5,∞). Key for parental lines: 1=L6038, 2=L7550, 3=P0159, 4=Nbh2189, 5=P6139, 6=B7262.

10.6 GxE

10.6.1 Rankings by environment

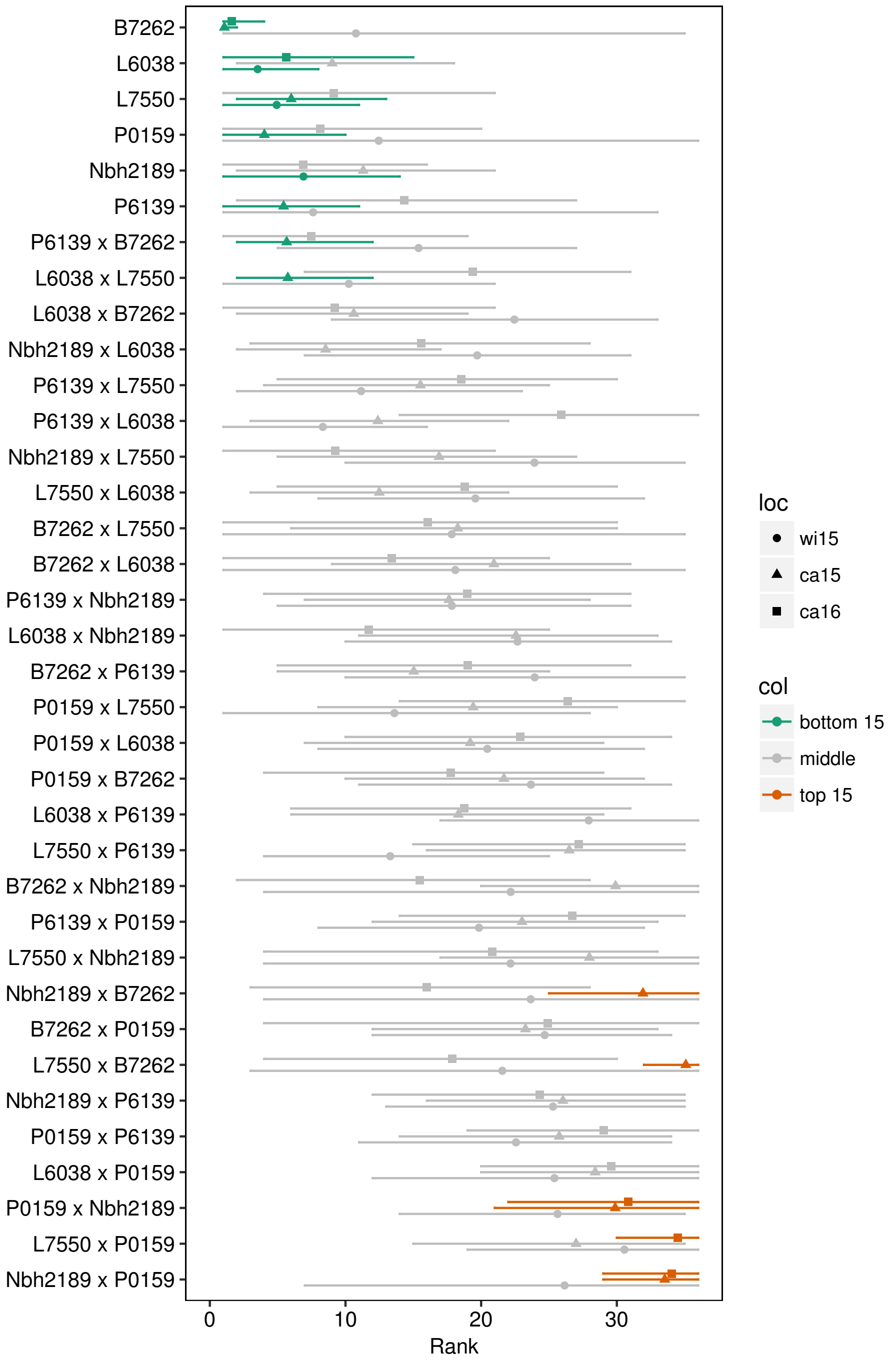

Figure S54: Mean values and 95% highest posterior density (HPD) intervals of hybrid rankings for dry root biomass (g; 130 DAP). Hybrids with intervals in the bottom 15 are shown in blue and hybrids with intervals in the top 15 are shown in red.

11. Shoot:root ratio

11.1 GGE biplot

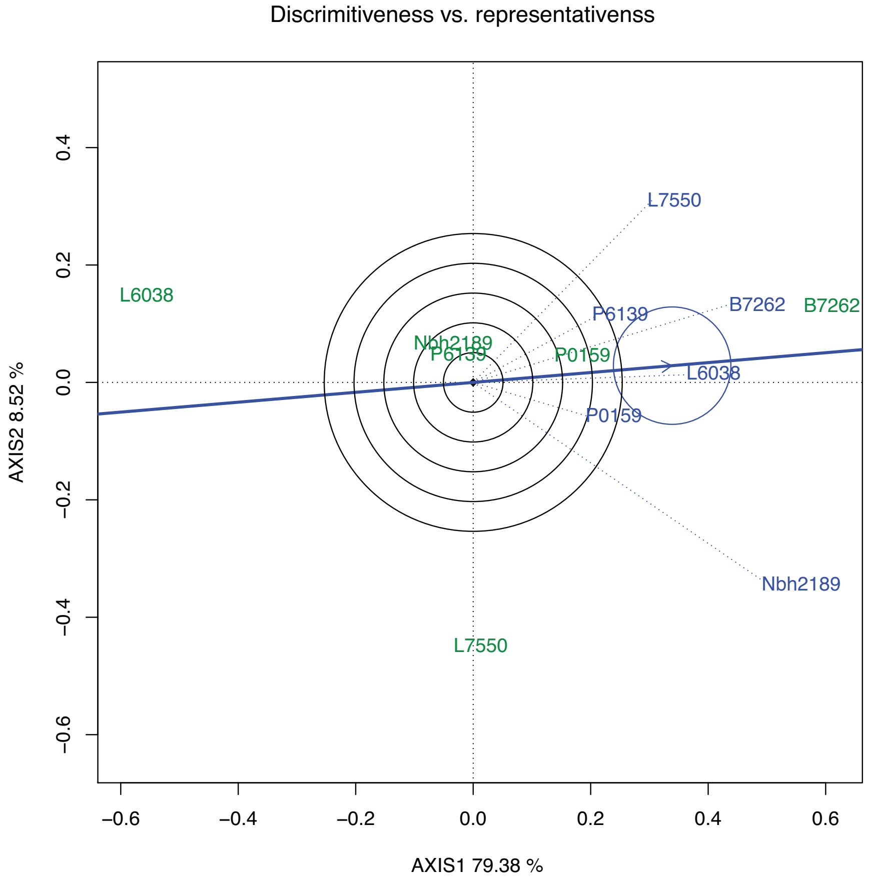

Figure S55: Discriminativeness vs. representativeness view of a biplot for shoot:root ratio (measured 130 days after planting). Parents treated as testers are shown in blue and as entries in green. The blue line indicates the average tester coordinate (ATC) abscissa, with the blue circle indicating the average tester score. The arrow in the circle indicates the direction of increasing GCA for the entries. Parent B7262 was the most discriminating, but parent L6038 was similarly discriminating and also the most representative of other parental lines (vector was close to the ATC abscissa).

11.2 Observed and predicted values

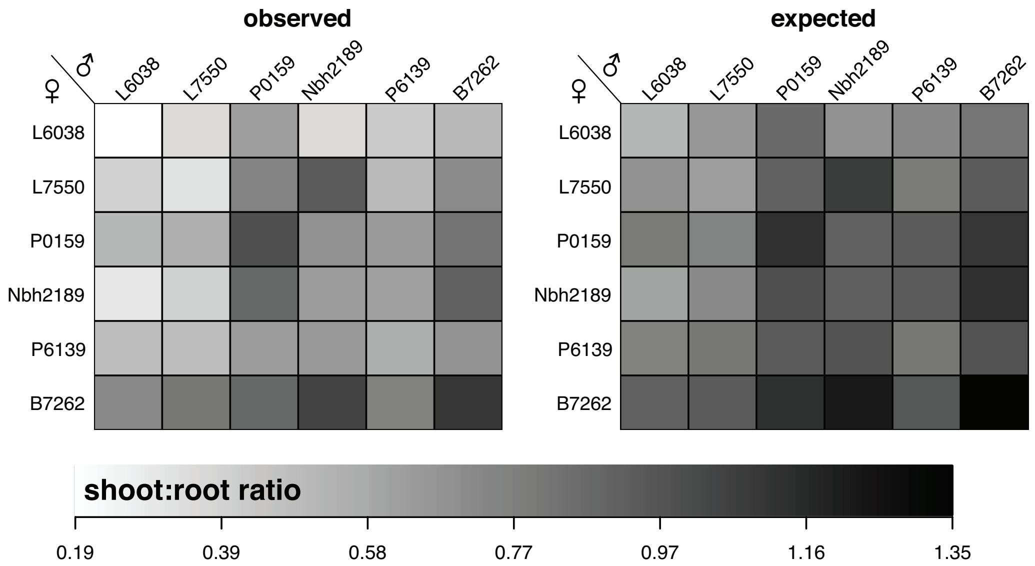

Figure S56: Observed (left) and predicted (right) means for shoot:root ratio. Posterior predicted means on the right are the result of fitting the model in BayesDiallel to the data on the left.

11.3 HPD intervals for inheritance classes

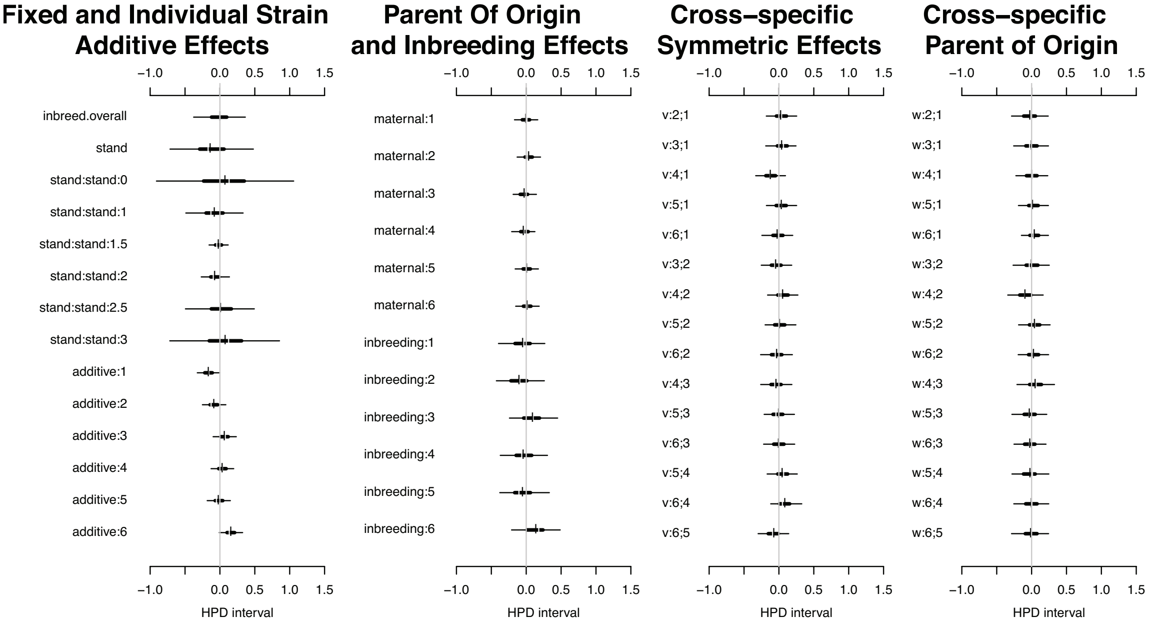

Figure S57: HPD intervals for shoot:root ratio.

11.4 HPD intervals for fixed and random effects

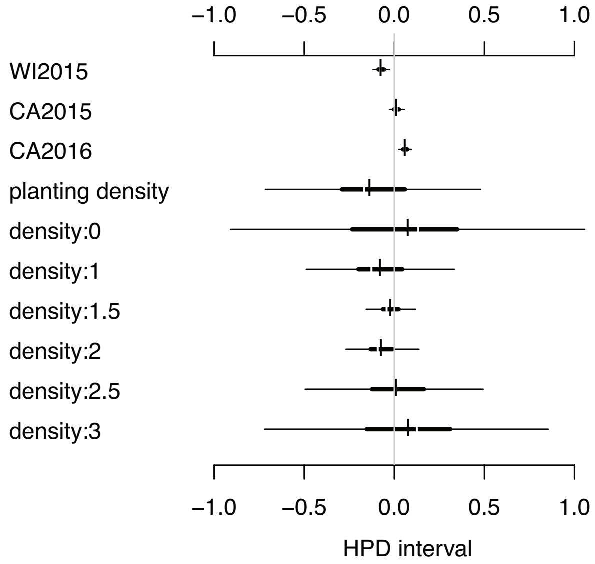

Figure S58: HPD intervals of fixed and random effects for shoot:root ratio. Planting density ranged from 0 (low) to 3 (high).

11.5 Degree of dominance

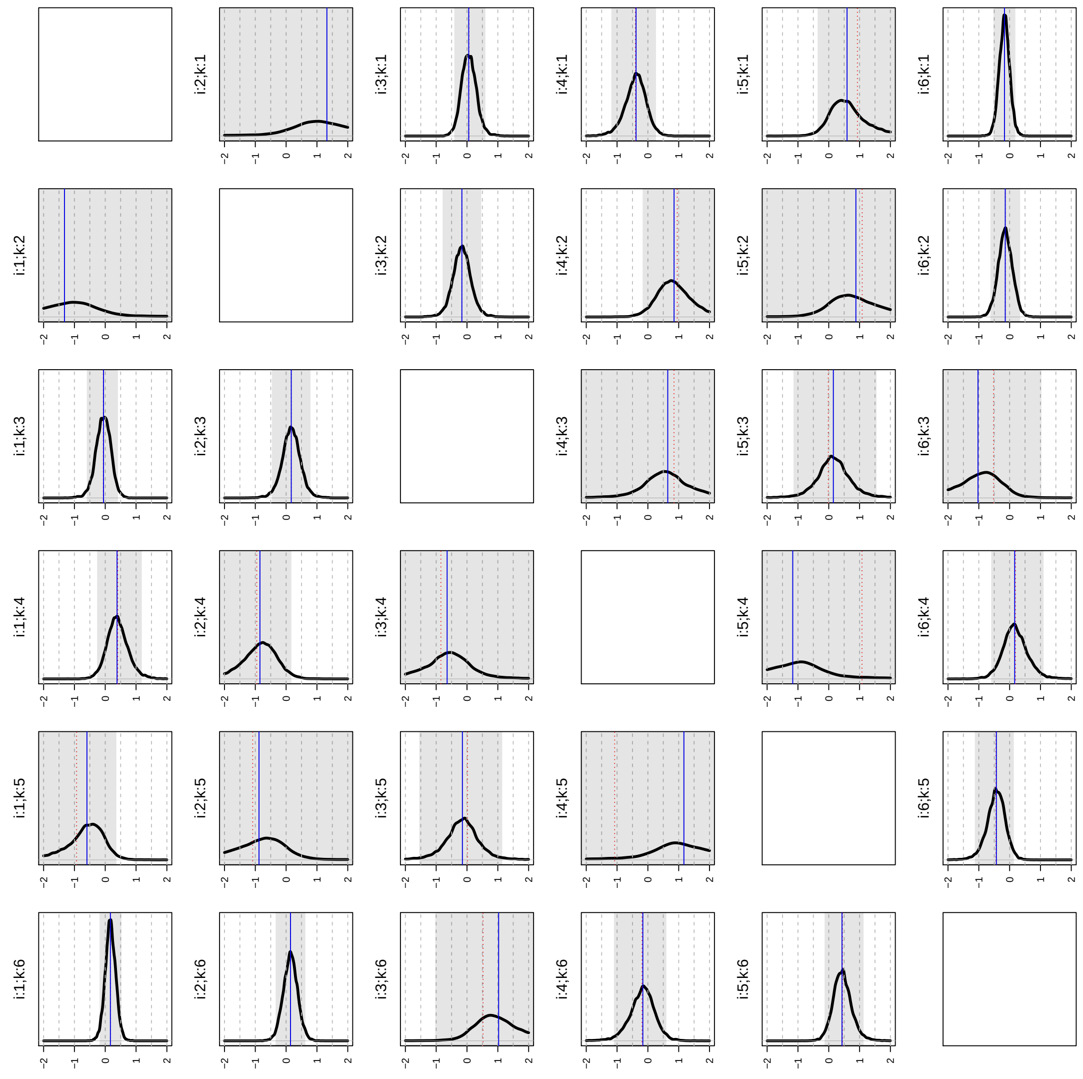

Figure S59: Posterior distributions of the degree of dominance for shoot:root ratio. Posterior means within the interval are indicated by red dotted lines and medians by blue solid lines, with 95% central quantiles shown in grey. A signal was considered strong if the mean, median, and the majority of the posterior distribution fell within the specified ranges: pseudo-underrecessive (-∞,-1.5), recessive (-1.5,-0.5), additive (-0.5,0.5), dominant (0.5,1.5), pseudo-overdominant (1.5,∞). Key for parental lines: 1=L6038, 2=L7550, 3=P0159, 4=Nbh2189, 5=P6139, 6=B7262.

11.6 GxE

11.6.1 GCA x environment interactions

Table S20: GCA for shoot:root ratio in each growing environment.

Parent	WI2015	CA2015	CA2016
L6038	-0.12**	-0.19***	-0.26***
L7550	-0.05	-0.03	-0.16**
P0159	0.1*	0.08*	0.1*
Nbh2189	-0.02	0.04	0.06
P6139	-0.04	-0.06	0.01
B7262	0.14**	0.16***	0.25***

***P≤0.001, **P≤0.01, *P≤0.05

Table S21: Nonparametric correlations (Spearman's rho) among environments for GCA of shoot:root ratio.

Environment	Spearman's rho
WI2015-CA2015	0.94**
WI2015-CA2016	1***
CA2015-CA2016	0.94**

***P≤0.001, **P≤0.01, *P≤0.05

11.6.2 SCA x environment interactions

Table S22: SCA for shoot:root ratio (g) in each growing environment.

F1 hybrids	WI2015	CA2015	CA2016
L6038 x L7550	0.14*	0	0.03
L6038 x P0159	0	0.04	0.1
L6038 x Nbh2189	0.13	-0.15*	-0.28***
L6038 x P6139	0.07	0.04	0.07
L6038 x B7262	-0.05	-0.02	-0.05
L7550 x P0159	0.01	-0.06	0.01
L7550 x Nbh2189	0.14	0.1	0.03
L7550 x P6139	-0.1	0.04	0.09
L7550 x B7262	-0.02	0.03	0
P0159 x Nbh2189	-0.01	-0.04	-0.09
P0159 x P6139	-0.09	0.01	-0.08
P0159 x B7262	-0.06	-0.02	-0.15
Nbh2189 x P6139	0.11	-0.02	0.05
Nbh2189 x B7262	0.09	0.16*	-0.02
P6139 x B7262	-0.07	-0.13	-0.08
P6139 x B7262	-0.19	-0.37	-0.03
Parental selfs			
L6038	-0.27**	0.09	0.08
L7550	-0.09	-0.11	-0.05
P0159	0.03	0.07	0.2*
Nbh2189	-0.18	-0.04	0.27**
P6139	0.02	0.05	-0.09
B7262	0.07	-0.02	0.11

***P≤0.001, **P≤0.01, *P≤0.05

Table S23: Nonparametric correlations (Spearman's rho) among environments for SCA of shoot:root ratio.

Environment	Spearman's rho
WI2015-CA2015	0.19
WI2015-CA2016	-0.03
CA2015-CA2016	0.37

***P≤0.001, **P≤0.01, *P≤0.05

11.6.3 Rankings by environment

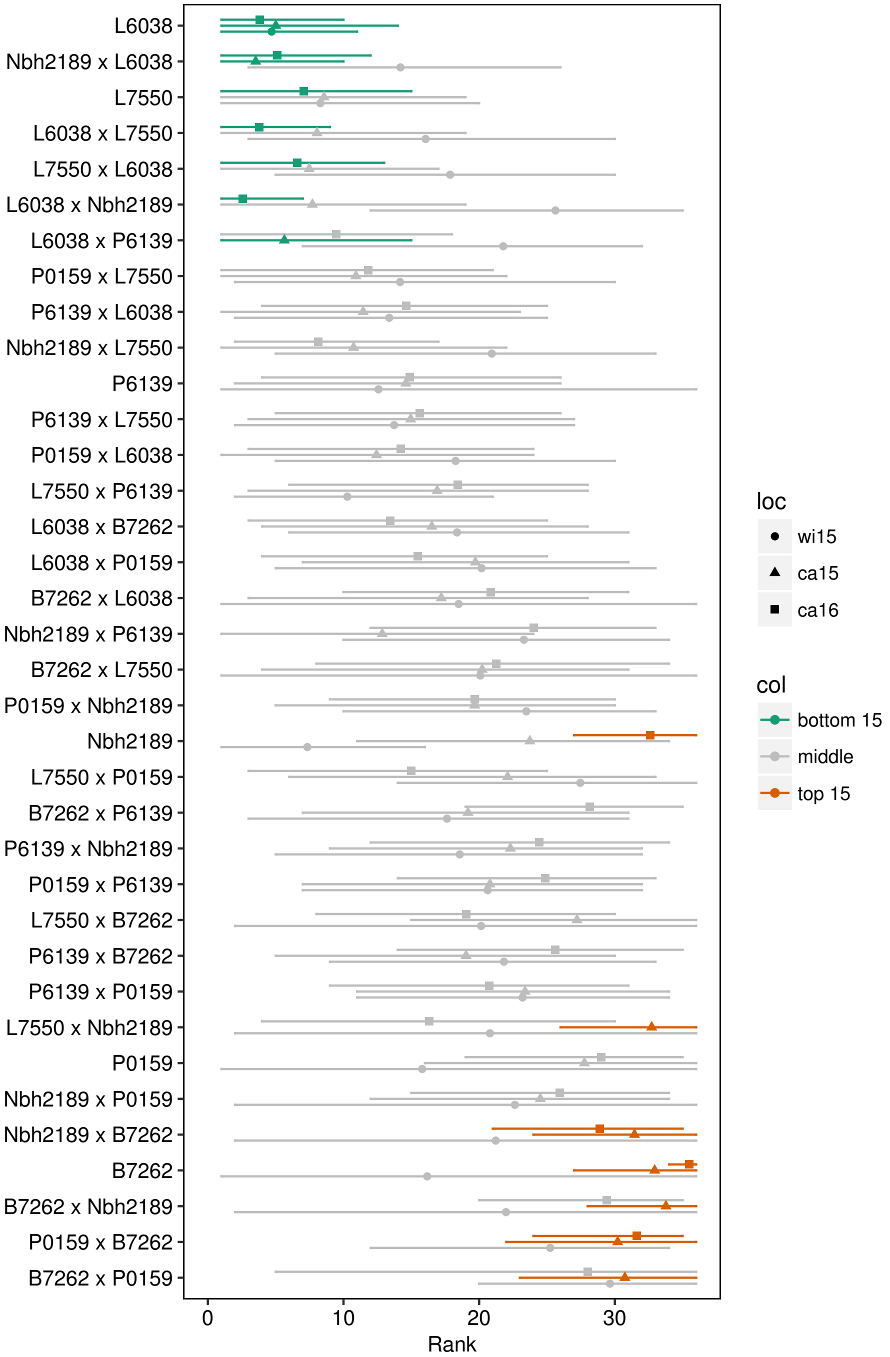

Figure S60: Mean values and 95% highest posterior density (HPD) intervals of hybrid rankings for shoot:root ratio (130 DAP). Hybrids with intervals in the bottom 15 are shown in blue and hybrids with intervals in the top 15 are shown in red.
